# Electron‐Rich Phenothiazine Congeners and Beyond: Synthesis and Electronic Properties of Isomeric Dithieno[1,4]thiazines

**DOI:** 10.1002/chem.202000137

**Published:** 2020-08-31

**Authors:** Lars May, Thomas J. J. Müller

**Affiliations:** ^1^ Institut für Organische Chemie und Makromolekulare Chemie Heinrich-Heine-Universität Düsseldorf Universitätsstrasse 1 40225 Düsseldorf Germany

**Keywords:** cyclic voltammetry, density functional calculations, structure–property relationships, thiazine, thiophene

## Abstract

A series of isomeric dithieno[1,4]thiazines is accessible through an intermolecular–intramolecular Buchwald–Hartwig amination starting from dihalodithienyl sulfides. The electronic properties of dithieno[1,4]thiazine isomers differ conspicuously over a broad range depending on the thiophene–thiazine anellation: a large cathodic (340 mV) or an anodic shift (130 mV) of the redox potentials relative to corresponding phenothiazines is possible. Structure–property relationships of the dithieno[1,4]thiazine constitution derived from DFT calculations and cyclic voltammetry not only reveal increased electron density but also different delocalization of the radical cations that determines the electrochemical properties significantly. In addition, photophysical properties (absorption and emission) qualify dithieno[1,4]thiazines as promising substitutes of phenothiazine and beyond due to increased tunable electron richness.

## Graphical abstract

**Structure–property relationships**: Four isomeric dithieno[1,4]thiazines, thieno congeners of phenothiazines, are synthesized by inter‐intra‐molecular Buchwald–Hartwig coupling. Their electronic properties open new avenues as donor systems for molecular electronics.

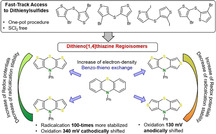

In the search of new and optimized applications of electroactive materials, such as in organic light‐emitting diodes,^1^ organic field‐effect transistors^2^ or photovoltaics,^3^ new structural motifs are increasingly demanded. Especially electron‐rich heterocyclic organic π‐systems like phenothiazines often favorably fulfill the requirements of organic electronics due to their inherently reversible oxidation potentials and favorable charge carrier transporting properties.^4^ Therefore, they have found entry in molecular electronics for diverse applications.^1^, ^5^ Phenothiazines’ promising electroactive properties are predominantly based on extraordinarily stable phenothiazinyl radical cations and low oxidation potentials.^6^, ^7^ Besides phenothiazine, an even more important building block in organic electronics is thiophene, due to its chemical stability and outstanding intrinsic charge carrier transport properties.^8^ Hence combining heterocyclic moieties in novel polyheterocyclic systems should establish a new class of electron‐rich electroactive donor compounds with promising properties. Consequently, dithieno[1,4]thiazines **2** with increased electron density and polarizability are derived from the phenothiazine moiety **1** by a topological benzo–thieno exchange. The redox potentials of the 4*H*‐dithieno[2,3‐b:3′,2′‐e][1,4]thiazines (**2 a**) are shifted almost 300 mV cathodically against the corresponding phenothiazines.^9^, ^10^ However, in theory there are six constitutional dithieno[1,4]thiazine isomers **2** conceivable from the three possible thiophene‐thiazine anellation modes: 2,3‐*b*‐anellation (*syn*), 3,2‐*b*‐anellation (*anti*), or 3,4‐*b*‐anellation (*exo*) (Scheme 1).

**Scheme 1:**
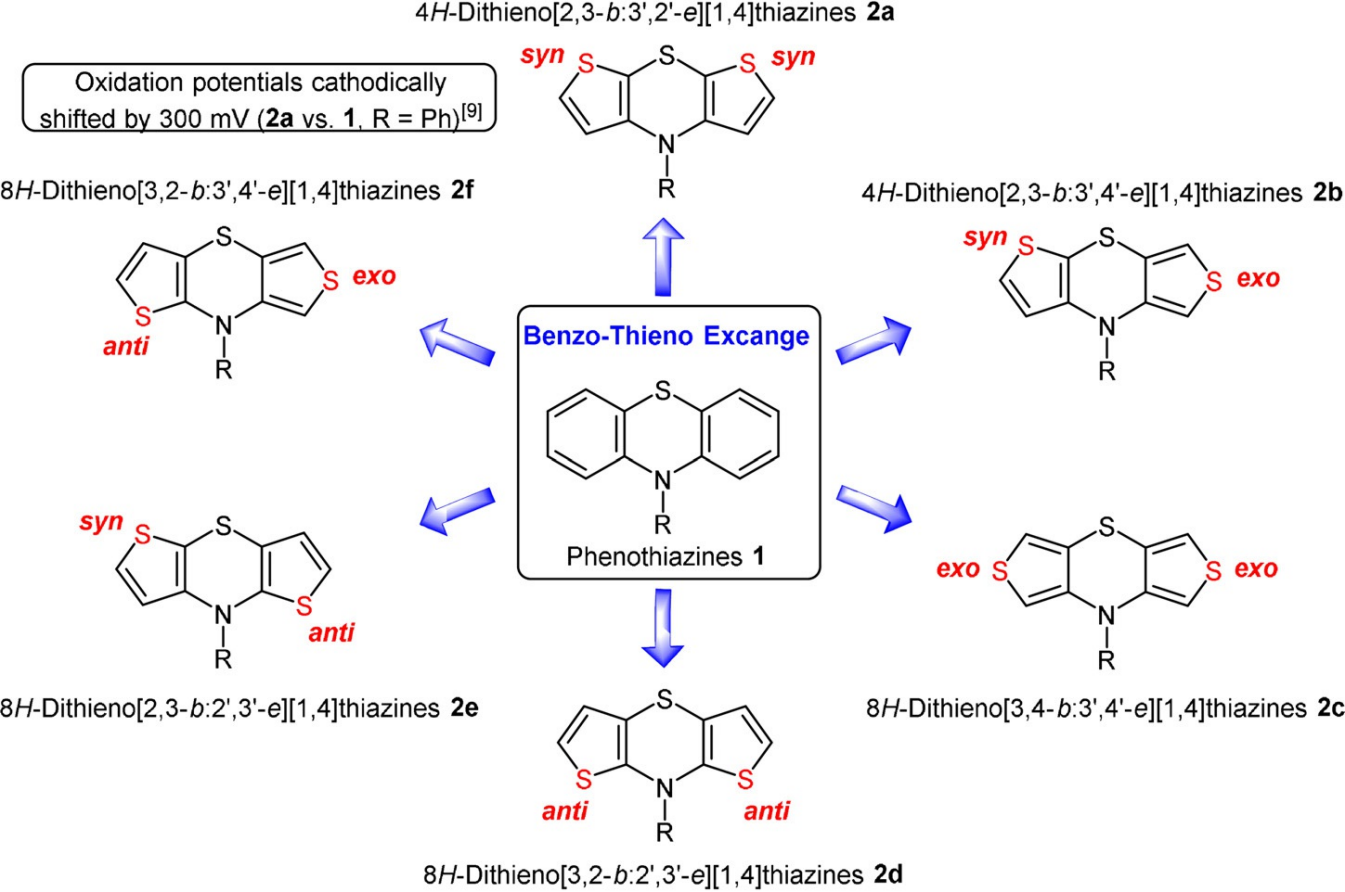
The six constitutional N‐substituted dithieno[1,4]thiazine isomers **2** — heterocyclic‐topological phenothiazine analogues.

In previous studies, we could show that photophysical and electrochemical properties of *syn‐syn* dithieno[1,4]thiazines **2 a** can be fine‐tuned by substitution^11^ or by benzoanellation.^12^ Constitutional modification of the established *syn*–*syn* dithieno[1,4]thiazine system **2 a** can prove to be favorable for the tuning of electronic properties and setting the stage for new applications. Herein, we communicate first syntheses, ground and excited state electronic properties and electronic structures of three novel dithieno[1,4]thiazine constitutional isomers (*syn*‐*exo* **2 b**, *exo*‐*exo* **2 c** and *anti*‐*anti* **2 d**, all with R=Ph) in comparison to the *syn‐syn* dithieno[1,4]thiazine isomer **2 a** (R=Ph) and the analogue phenothiazine **1** (R=Ph).

The known synthesis of the *syn*‐*syn*‐dithieno[1,4]thiazines **2 a** requires the brominated dithienyl sulfide **3 d** as a starting material, which can be accessed by a disadvantageous three‐step procedure involving the synthesis of sulfur dichloride using chlorine gas.^9^ Since one‐pot processes have been successfully established in many syntheses of functional *π*‐systems,^14^ we transferred a previously reported one‐pot synthesis of dithienothiophenes^13^ to a one‐pot synthesis of dithienyl sulfides **3** (Scheme 2). By modification of the reaction conditions four dithienyl sulfides **3** were synthesized in good to very good yields through this consecutive three‐component process. Besides the high yields, which are comparable to the overall yields of the single‐step transformations,^15^ both unsymmetrical (**3 a**) and symmetrical dithienyl sulfides (**3 b**–**d**) could be accessed. Moreover, as the synthesis of unsymmetrical dithienyl sulfides would have required additional synthetic steps,^15^ this divergent one‐pot strategy is particularly advantageous.

**Scheme 2:**
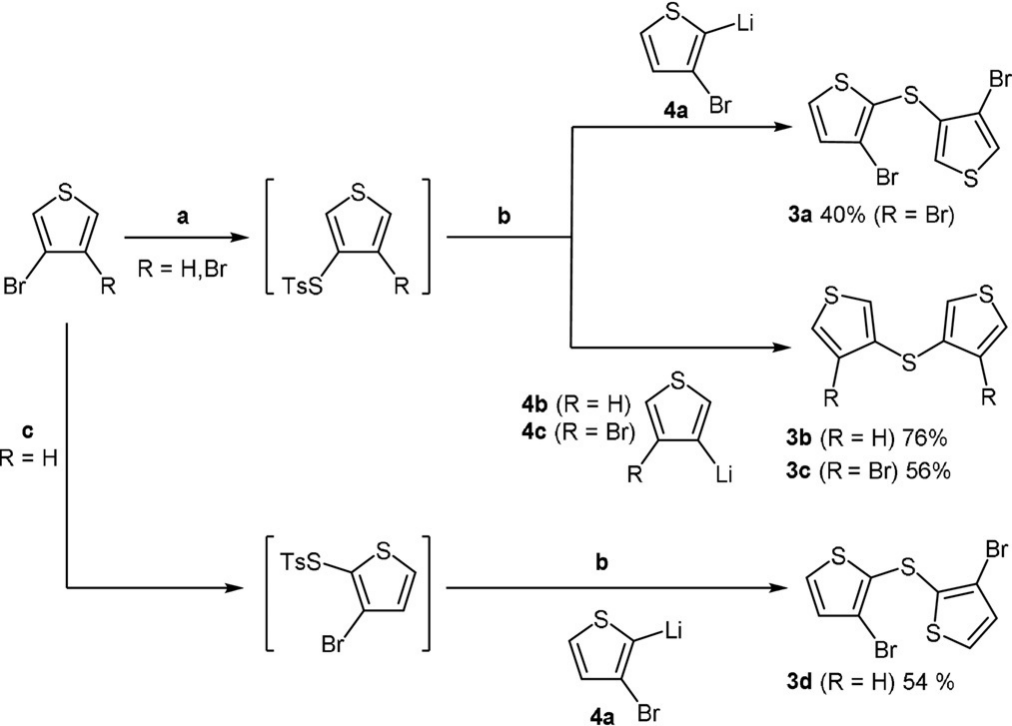
One‐pot synthesis of dithienyl sulfides **3** via consecutive three‐component sequences: a) *n*BuLi (1.0 equiv), Et_2_O, −78 °C; then: S_8_ (1.0 equiv), −78 to 0 °C; then: *p*TsCl (1.0 equiv), 0 to 40 °C; b) thienyl lithium **4** (1.2 equivs), −78 °C; c) LDA (1.0 equiv), Et_2_O, −78 °C; then: S_8_ (1.0 equiv), −78 to 0 °C; then: *p*TsCl (1.0 equiv), 0 to 40 °C.

For the synthesis of the novel *anti*‐*anti*‐dithieno[1,4]thiazine isomer **2 d** through a Buchwald–Hartwig amination (Table 1, entry 3), dithienyl sulfide **3 b** was first iodinated by using *N*‐iodosuccinimide (Scheme 3).

**Table 1:** Synthesis of three novel dithieno[1,4]thiazine isomers **2** via twofold Buchwald–Hartwig amination.

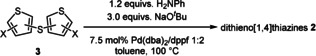		
Entry	Dithienyl sulfide **3**	Dithieno[1,4]thiazine **2** (yield [%])^[a]^
1	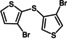	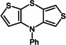
	**3 a**	**2 b** (71)
2	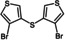	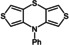
	**3 c**	**2 c** (22)
3	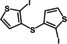	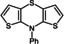
	**3 e**	**2 d** (38)

[a] Yields after column chromatography.

**Scheme 3:**
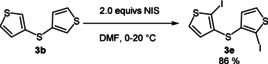
Iodination of dithienyl sulfide **3 b**.

Finally, the novel dithieno[1,4]thiazine isomers (*syn*‐*exo* **2 b** and *exo‐exo* **2 c**) were synthesized by twofold intermolecular–intramolecular Buchwald–Hartwig amination from the brominated dithienyl sulfides **3 a** and **3 c** and aniline, bearing phenyl as an electronically neutral *N*‐aryl substituent, with moderate to high yields adapting the literature known procedure (Table 1, entries 1, 2), which was established for the synthesis of *syn*‐*syn*‐dithieno[1,4]thiazine **2 a**.^9^

The electronic properties of the dithieno[1,4]thiazines **2 b**, **2 c**, and **2 d** were experimentally assessed by cyclic voltammetry, as well as by absorption and emission spectroscopy (Table 2) and were compared to the corresponding *syn*‐*syn* isomer **2 a** and *N*‐phenylphenothiazine (**1**).^9^ A deeper insight into photophysical and electrochemical properties, as well as electronic structures was gained by the accompanying DFT calculations. The ground‐state geometries were optimized using the B3LYP or the uB3LYP functional^16^ and the 6–311G* basis set^17^ as implemented in the Gaussian 09 program package.^18^ Excitation energies and the excited state geometry (S_1_) of *anti*‐*anti*‐dithieno[1,4]thiazine **2 d** were calculated with TD‐DFT^19^ methods as implemented in the Gaussian 09 program package using the same functional and basis set as for the ground state optimizations. All optimized geometries were confirmed as minima by analytical frequency analyses. The polarizable continuum model (PCM) or the SMD solvation model with dichloromethane as a solvent was applied for the calculations as indicated, since all measurements were performed in dichloromethane solutions.^20^

**Table 2:** Selected photophysical and electrochemical properties of dithieno[1,4]thiazines **2** compared to phenothiazine **1**.

Entry	*E*_0/+1_ [mV]^[a]^	*E*_+1/+2_ [mV]^[a]^	*K*_SEM_^[b]^	*λ*_max,abs_ [nm]	*λ*_max,em_ [nm]^[d]^	*E*_0‐0_ [eV]^[e]^	Δ$\tilde{\nu }$ [cm^−1^]^[f]^
				(*ϵ* [L mol^−1^⋅cm^−1^])^[c]^			
**2 a** 9	390	1260	5.57×10^14^	385 (380)	438	2.95	3140
				319 (5650)			
				248 (19 400)			
**2 b**	580	1430	2.55×10^14^	336 (5310)	405	4.40	5070
				253 (18930)			
**2 c**	850	–	–	317 (20160)	374	3.61	4810
				247 (25480)	354		
**2 d**	370	1290	3.92×10^15^	386 (730)	516	2.69	6940
				300 (1540)			
				242 (8250)			
**1** 9	720	1520	3.63×10^13^	323 (4550)	447	3.14	8590
				258 (84000)

[a] Half‐wave potentials, 0.1 m [Bu_4_N][PF_6_], *v*=100 mV s^−1^, Pt‐working, Ag/AgCl‐reference and Pt‐counter electrode, [Me_10_Fc]/[Me_10_Fc]^+^ as an internal standard. [b] *K*_SEM_=10^(*E*(+1/+2)‐*E*(0/+1))/59^ ^mV^; [c] Recorded in CH_2_Cl_2_ at *T=*298 K, *c*(**2**/**1**)=10^−5^ m. [d] Recorded in CH_2_Cl_2_ at *T=*298 K, *c*(**2**/**1**)=10^−6^ m. [e] *E*_0‐0_ was determined from the cross‐section of absorption and emission spectra. [f] Δ$\tilde{\nu }$ =1/*λ*_max,abs_ −1/*λ*_max,em_.

The ground‐state properties, examined by cyclic voltammetry, revealed that, with exception of *exo*‐*exo*‐dithieno[1,4]thiazine **2 c**, dithieno[1,4]thiazines **2** possess two clearly separated electrochemically reversible one‐electron oxidations (Figure 1).

**Figure 1:**
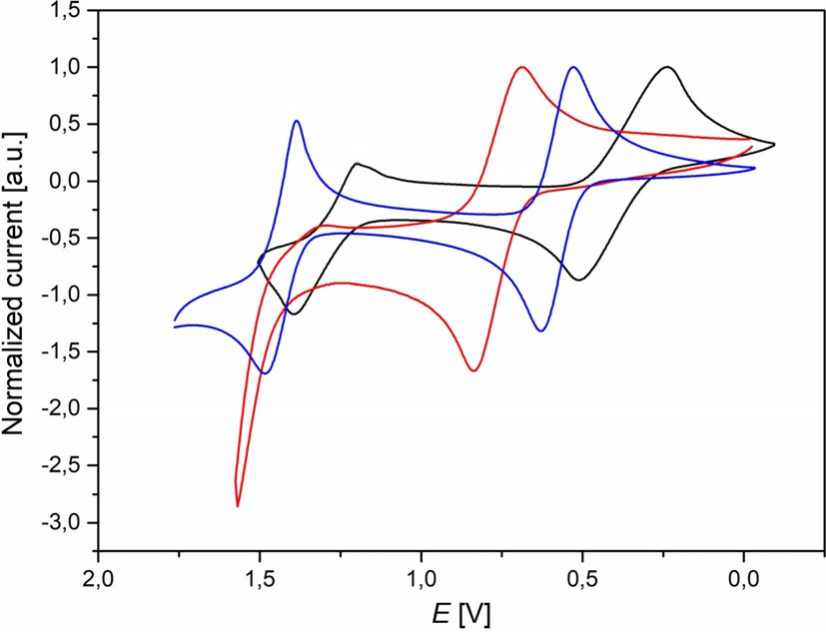
Cyclic voltammograms of the dithieno[1,4]thiazines **2 b** (blue), **2 c** (red) and **2 d** (black), (0.1 m [Bu_4_N][PF_6_], *v*=100 mV s^−1^, Pt‐working, Ag/AgCl‐reference and Pt‐counter electrode, [Me_10_Fc]/[Me_10_Fc]^+^ as an internal standard; Me_10_Fc=decamethylferrocene).

As a consequence, they can be described as Weitz‐type redox systems.^7^ Interestingly, the oxidation potentials of the isomers **2** range from 370 to 850 mV, which already accounts for wide potential tunability in comparison to phenothiazine **1** (*E*_0/+1_ *=*770 mV).^9^ The anellation of thiazine and thiophene determines the redox potentials of the corresponding dithieno[1,4]thiazines significantly (Table 2). Whereas the redox potential of *exo*‐*exo*‐dithieno[1,4]thiazine **2 c** is shifted about 130 mV anodically against phenothiazine **1**, the redox potential of *anti*‐*anti*‐dithieno[1,4]thiazine **2 d** is shifted about 340 mV cathodically against **1**. *syn*‐*syn*‐Dithieno[1,4]thiazine **2 a** is slightly shifted anodically against *anti*‐*anti*‐dithieno[1,4]thiazine **2 d**. The redox potential of *syn*‐*exo*‐dithieno[1,4]thiazine **2 b** is about the mean of the redox potentials of **2 a** and **2 c**, which is in line with the thiophene anellation originating partially from **2 a** and **2 c**. The second oxidation potentials appear in the range of 1300 to 1400 mV and are shifted cathodically relative to phenothiazine **1** by about 100 to 250 mV. Calculation of semiquinone constants *K*_SEM_ 21 from the redox potentials elucidate the relative stabilities of the radical cations of dithieno[1,4]thiazines (Table 2). Again, the mode of anellation largely affects the stability of the radical cations. While *K*_SEM_ values of dithieno[1,4]thiazines **2 a** and **2 b** surmount that of phenothiazine **1** by one order of magnitude, the *K*_SEM_ value of dithieno[1,4]thiazine **2 d** is even about 100 times higher than that of phenothiazine **1**.

All DFT‐calculated ground‐state geometries reveal a folding along the *S,N*‐axis forming a butterfly structure (*ϑ*(**2 a**,**2 d**)*=*144°; *ϑ*(**2 b**)*=*155°; *ϑ*(**2 c**)*=*158°; *ϑ*(**1**)*=*148°) and tend to planarize upon oxidation as known for phenothiazines (Figure 2).^22^ According to DFT calculations, the *intra* conformer of **2 a** is slightly preferred over its *extra* conformer similar to phenothiazine **1**, which however exhibits a larger difference of free energies (ΔΔ*G*(**2 a**)*=*1.01 kcal mol^−1^, ΔΔ*G*(**1**)*=*1.89 kcal mol^−1^, B3LYP/6–311G* PCM CH_2_Cl_2_), suggesting an *extra*‐*intra* Boltzmann distribution in solution. The calculations on the isomers **2 b**–**d** also suggest the latter (see Supporting Information Chapter 6.1).

**Figure 2:**
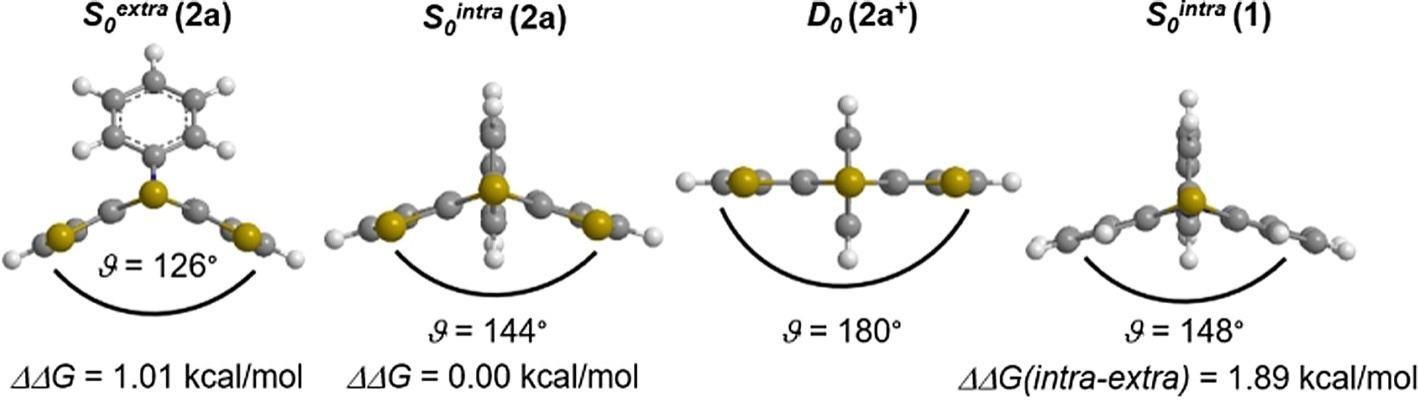
Exemplary *S*,*N*‐folding angles *ϑ* and optimized ground state geometries of *extra* and *intra* conformers (*S*_0_^*extra*^ and *S*_0_^*intra*^) and of the radical cation (*D*_0_) of *syn*‐*syn*‐dithieno[1,4]thiazine **2 a** as well as ground state geometry of *intra*‐phenothiazine **1** *S*_0_^*intra*^ (B3LYP/6–311G*, PCM CH_2_Cl_2_; uB3LYP for D_0_).

However, the experimental oxidation potentials cannot be correlated with the HOMO energy levels (B3LYP/6–311G* PCM CH_2_Cl_2_) reflecting the electron richness of the *extra* conformers, whereas the *intra* conformations might dominate the redox chemistry of dithieno[1,4]thiazines (see Supporting Information Chapter 6.2).

The hierarchy of the redox potentials qualitatively follows the HOMO energy levels of the *intra* conformers (Figure 3; for the correlation, see the Supporting Information Chapter 4), underlining the dependence of the oxidizability on the thiophene anellation, as well as the ease of oxidizability upon topological benzene–thiophene exchange. Surprisingly, the HOMO energy levels of the *exo*‐dithieno[1,4]thiazines **2 b** and **2 c** did not fit with the trend of the experimentally determined redox potentials (Table 2). Therefore, we calculated the redox potentials of the compounds **2** and **1** according to a DFT‐based literature procedure^23^ giving rise to an almost perfect correlation (*r* ^2^ *=*0.9996) of experimental and calculated data (Figure 4). Even the calculated gas‐phase redox potentials are perfectly in line with cyclic voltammetry, excluding a large solvation effect on the potentials (see the Supporting Information Chapter 6).

**Figure 3:**
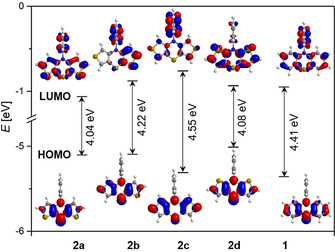
DFT‐computed Kohn–Sham FMOs of the compounds **2** and **1** (B3LYP/6–311G*, PCM CH_2_Cl_2_, isosurface value at 0.04 a.u.).

**Figure 4:**
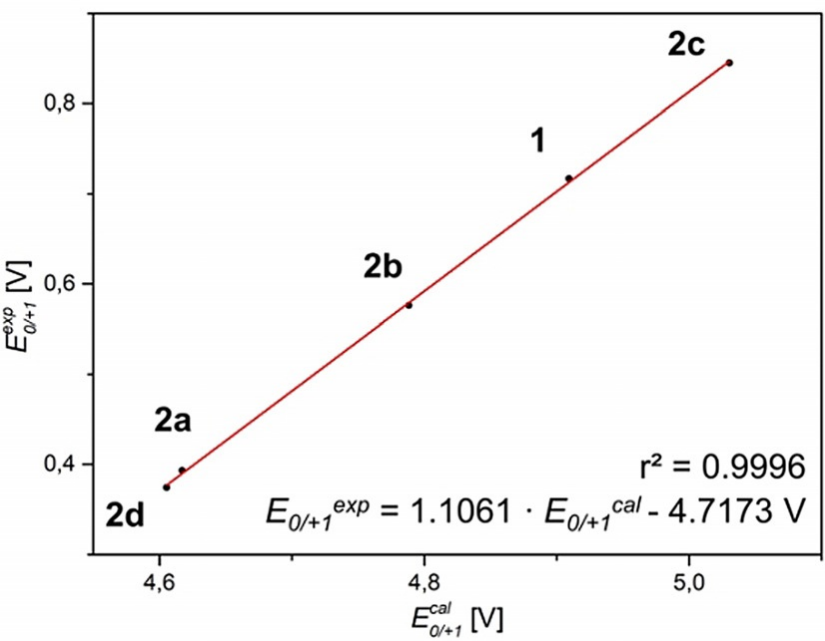
Linear correlation of the measured first oxidation potentials *E*_0/+1_^*exp*^ (0.1 m [Bu_4_N][PF_6_], *v*=100 mV s^−1^, Pt‐working, Ag/AgCl‐reference and Pt‐counter electrode, [Me_10_Fc]/[Me_10_Fc]^+^ as an internal standard) and the DFT‐calculated first oxidation potentials *E*_0/+1_^*cal*^ (vs. vacuum, uB3LYP/6–311G*, SMD CH_2_Cl_2_) of the compounds **2** and **1**.

As a consequence, the correlation of redox potentials cannot exclusively be explained by the electron richness indicated by the HOMO energy levels. Expectedly, relative stabilities of the radical cations are significantly influenced by conjugation effects, which can be visualized by analyzing Wiberg bond orders (Figure 5).

**Figure 5:**
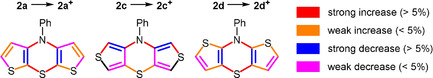
Relative change of the Wiberg bond‐orders from neutral ground state to the oxidized species (radical cation) in the dithieno[1,4]thiazine core of compounds **2 a^+^**, **2 c^+^** and **2 d^+^** (uB3LYP/6–311G*).^24^ For a detailed list of bond orders including phenothiazine radical cation **1^+^**, see Supporting Information Chapter 6.

The changes of Wiberg bond orders in the dithieno[1,4]thiazine core comparing neutral ground states and the radical cations allow conclusions with respect to the radical delocalization. For example, the relatively strong increase of the thiazine‐*CS* and *CN*‐bond orders (about 7 %) as well as the strong decrease of the thiazine *CC*‐bond orders (about 8 %) indicate, that in oxidized *syn*‐*syn* dithieno[1,4]thiazine **2 a^+^** and *anti*‐*anti*‐dithieno[1,4]thiazine **2 d^+^** the radical is strongly delocalized on the thiazine ring. In addition, for **2 d^+^** the radical should be also delocalized on the anellated thiophenes, more noticeably yielding a more phenothiazine‐like delocalization than in **2 a^+^** as indicated by a stronger equalization of the thiophene‐bond orders. In contrast the delocalization in *exo*‐*exo* dithieno[1,4]thiazine **2 c^+^** focusses mainly on the formal (2,2’‐dithio)divinylamine system and the interaction via the thiazine‐ring is weaker. This might explain the unexpected low stability of the radical cations of the *exo*‐isomers **2 b^+^** and **2 c^+^**. Furthermore, the radical delocalization illustrated by the spin‐density plots is in good agreement with the bond order analysis (Figure 6). As already implied by the redox potentials (Table 2), the radical cation of dithieno[1,4]thiazine **2 b^+^** clearly presents itself as a superposition of **2 a^+^** and **2 c^+^**.

**Figure 6:**
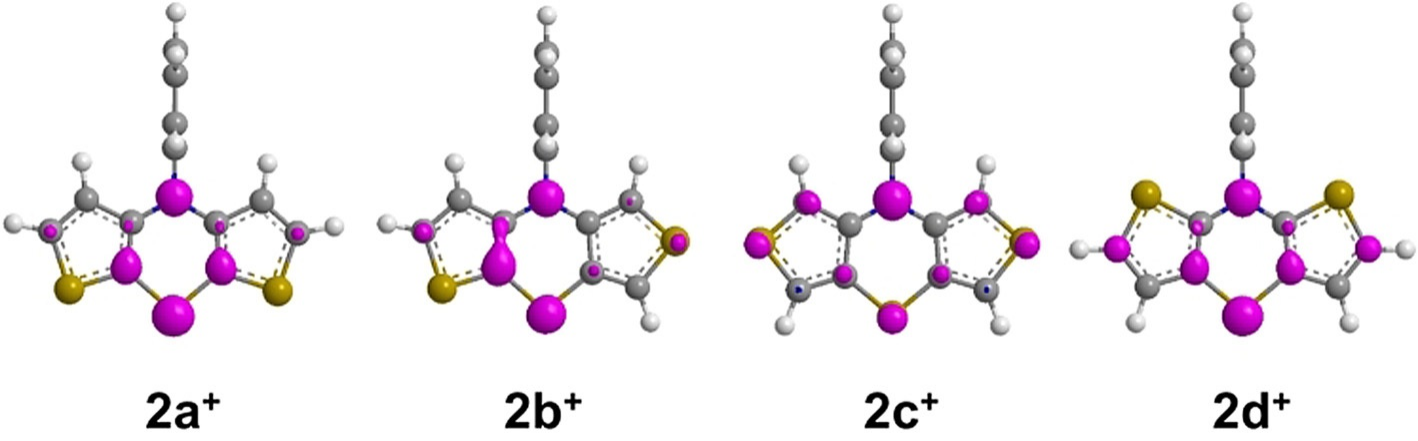
Spin‐density plots of the radical cations of dithieno[1,4]thiazines **2^+^** (uB3LYP/6‐311G*).

The very exact correlation of DFT‐calculated redox potentials and cyclic voltammetry allows predicting the redox potentials of the dithieno[1,4]thiazine isomers **2 e** and **2 f** (Figure 7) yet to be synthesized. Surprisingly, *syn*‐*anti*‐dithieno[1,4]thiazine **2 e** turns out to be the isomer with the lowest oxidation potential, yet with only a slight cathodic shift compared to *anti*‐*anti*‐isomer **2 d**.

**Figure 7:**
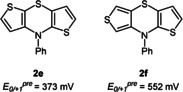
Predicted redox potentials *E*_0/+1_^*pre*^ of the isomers **2 e** and **2 f**.

The excited‐state properties, examined by absorption and emission spectroscopy in dichloromethane solutions (Table 2), revealed that the absorption behavior is as qualitatively well in line with the thiophene anellation mode. TDDFT calculations indicate that the longest wavelength absorption maxima mostly originate from HOMO–LUMO transitions (Table 3) and that the HOMO–LUMO gap increases with decreasing HOMO energy level (Figure 3). Furthermore, the calculated longest wavelength absorption bands are experimentally not detected in every case, presumably to superposition by the neighboring more intense shorter wavelength band. As already seen for the electrochemical properties, the UV/Vis absorption spectra cannot be satisfactorily reproduced exclusively from *extra* conformers, supporting that *intra* conformers strongly affect the photophysical properties as well. For instance, the TDDFT calculated absorption spectrum of **2 d** in its *extra* conformation does not fit the experimental spectrum with respect to the transition energies and exhibits low oscillator strengths similar to the *intra* conformer (see Supporting Information Chapter 6.1.1).

**Table 3:** TDDFT calculations on the UV/Vis absorption maxima of compounds **1** and **2** (B3LYP/6–311G*, PCM CH_2_Cl_2_).

	*λ*_max,exp_ [nm]^[a]^	*λ*_max,calcd_	Oscillator	Most dominant
	(*ϵ* [L mol^−1^⋅cm^−1^])	[nm]	strength *f*	contributions
**2 a**	385 (380)	395	0.0044	HOMO→LUMO (96 %)
	319 (5650)	324	0.0771	HOMO→LUMO+2 (97 %)
	248 (19400)	254	0.1784	HOMO−1→LUMO (77 %)
**2 b**	–^[b]^	352	0.0225	HOMO→LUMO (83 %)
	336 (5310)	326	0.0893	HOMO→LUMO+2 (76 %)
	253 (18930)	264	0.1263	HOMO−1→LUMO (79 %)
**2 c**	–^[b]^	328	0.0125	HOMO→LUMO (72 %)
	317 (20160)	316	0.0537	HOMO→LUMO+1 (71 %)
	247 (25480)	294	0.2299	HOMO→LUMO+3 (90 %)
**2 d**	386 (730)	383	0.0609	HOMO→LUMO (96 %)
	300 (1540)	315	0.0209	HOMO→LUMO+3 (90 %)
	242 (8250)	253	0.1317	HOMO−1→LUMO (87 %)
**1**	323 (4550)	346	0.0020	HOMO→LUMO (95 %)
	258 (84000)	255	0.4817	HOMO−1→LUMO (81 %)

[a] Recorded in CH_2_Cl_2_, *c*(**1**/**2**)=10^−5^ m, *T=*293 K. [b] Not observed due to superposition.

All dithieno[1,4]thiazines **2** as well as phenothiazine **1** fluoresce weakly in dichloromethane solutions (Table 2; for absorption and emission spectra plots, see the Supporting Information Chapter 5). For illustration, the fluorescence quantum yield *Φ*_f_ of *anti*‐*anti* dithieno[1,4]thiazine **2 d** in dichloromethane is 0.03,^25^, ^26^ which matches with analogous phenothiazines.^27^ In accordance with phenothiazines,^28^ **2 d** planarizes after photoexcitation to the vibrationally relaxed excited S_1_ state, which rationalizes the relatively large Stokes shift (Figure 8).

**Figure 8:**
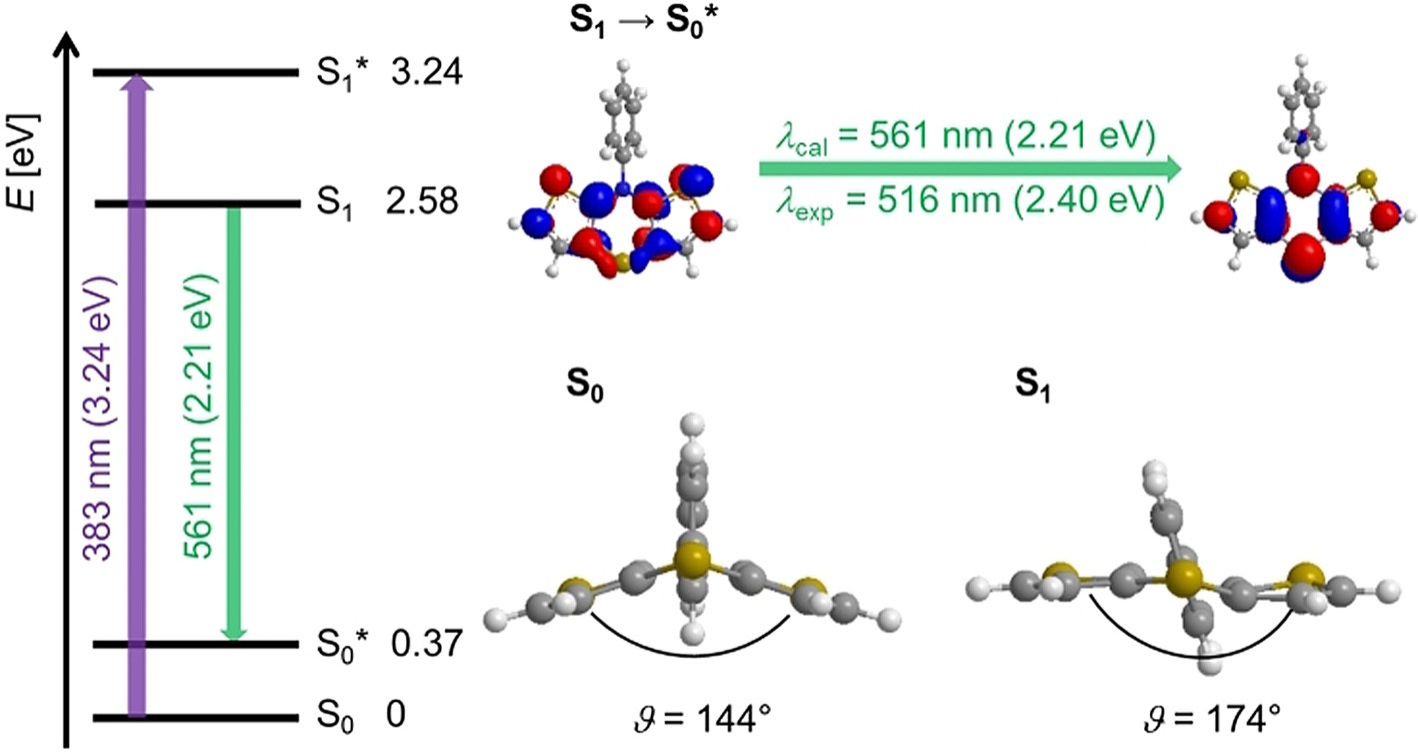
DFT‐computed Jablonski diagram, Kohn–Sham FMOs corresponding to the S_1_–S_0_*‐transition (fluorescence) and optimized geometries of S_0_ and S_1_ with *S*,*N*‐folding angles *ϑ* of *anti*‐*anti*‐dithieno[1,4]thiazine **2 d** (B3LYP/6–311G*, PCM CH_2_Cl_2_, isosurface value at 0.04 a.u.).

This holds true for all dithieno[1,4]thiazine isomers **2** taking into account the similar Stokes shifts. Hence, the Stokes shifts of the *exo* isomers **2 b** and **2 c** are smaller as a consequence of their less folded S_0_‐geometries according to DFT calculations. Hence, smaller structural changes upon photoexcitation can be expected. Therefore, the relatively small Stokes shift of *syn*‐*syn*‐dithieno[1,4]thiazine **2 a** might indicate emission from a higher excited state.

Three novel dithieno[1,4]thiazine isomers **2 b**, **2 c**, and **2 d** were rapidly synthesized by intermolecular–intramolecular Buchwald–Hartwig [5+1] anellation. For the efficient preparation of the dithienyl sulfide precursors **3**, a one‐pot synthesis has been developed. The dithieno[1,4]thiazine isomers present themselves as electron‐rich heterocyclic‐topological phenothiazine congeners, yet, with an electronic structure strongly depending on the mode of thiophene anellation. On the one hand, the redox potential can be cathodically shifted up to 340 mV compared the corresponding phenothiazine **1**, but on the other hand, just by changing the thiophene‐thiazine connectivity, the potential can be anodically shifted by about 130 mV. The dithieno[1,4]thiazines’ semiquinone formation constants *K*_SEM_ imply that their radical cations are even more stabilized than phenothiazine radical cations. Ultimately, the radical cation *anti*‐*anti*‐dithieno[1,4]thiazine **2 d^+^** is 10‐times more stable relative to the radical cation *syn*‐*syn* **2 a^+^** and 100‐times more stable relative to phenothiazine radical cation **1^+^**. DFT calculations on the radical cations (spin densities and bond orders) and the redox potentials elucidate different radical delocalization in the isomers. The photophysical properties of the dithieno[1,4]thiazines **2** are in line with the corresponding phenothiazine. As for the oxidation potentials absorption maxima can also be tuned by the mode of anellation.

In summary *anti*‐*anti*‐dithieno[1,4]thiazine **2 d** possesses the most extended delocalization, thus the most stabilized radical cation as well as the lowest oxidation potential in the series. All this renders this isomer as a promising phenothiazine substitute for applications in materials sciences.^5^, ^13^ As a consequence of the experimentally and computationally established structure‐property correlations, the redox potentials of the two remaining dithieno[1,4]thiazine isomers can be predicted. Especially, the *syn*‐*anti*‐isomer **2 e** should have the lowest oxidation potential of all isomers and appears to be another promising candidate for materials science applications. Further studies on syntheses, properties and applications of dithieno[1,4]thiazines are currently underway.

## Experimental Section

### Synthesis of 8‐phenyl‐8*H*‐dithieno[3,2‐b:2′,3′‐e][1,4]thiazine (2 d) by Buchwald–Hartwig amination

In a flame‐dried Schlenk vessel under nitrogen atmosphere were placed bis(2‐iodothiophen‐3‐yl)sulfane (**3 e**) (1427 mg, 3.170 mmol, 1.000 equiv), aniline (354.0 mg, 3.800 mmol, 1.200 equiv), bis(dibenzylideneacetone)palladium(0) (137.0 mg, 238.0 μmol, 7.5 mol %), 1,1’‐bis(diphenylphosphano)ferrocene (263.0 mg, 476.0 μmol, 15 mol %), sodium *tert*‐butoxide (914.0 mg, 9.510 mmol, 3.000 equiv) and dry toluene (19.0 mL). After degassing with nitrogen for 5 min, the reaction solution was stirred at 100 °C for 19 h. The volatiles were removed by evaporation and the crude product was purified by chromatography on silica gel (*n*‐hexane) and by recrystallization from ethanol to give compound **2 d** (352 mg, 38 %) as a yellow solid. M.p.: 97–99 °C. *R*_f_ (*n*‐hexane)=0.35. ^1^H NMR (300 MHz, [D_6_]acetone): *δ*)6.59 (d, ^*3*^ *J_HH_*=5.62 Hz, 2 H), 6.93 (d, ^*3*^ *J_HH_*=5.62 Hz, 2 H), 7.31–7.34 (m, 1 H), 7.54–7.57 (m, 4 H); ^13^C NMR (125 MHz, [D_8_]THF): *δ*=112.6 (C_quat_), 117.6 (CH), 125.2 (CH), 127.2 (CH), 128.4 (CH), 130.9 (CH), 144.0 (C_quat_), 146.4 (C_quat_); MS(MALDI‐TOF): *m*/*z*: 287.414 ([*M*]^+^); IR: $\tilde{\nu }$ =1722 (w), 1582 (m), 1537 (w), 1512 (w), 1458 (m), 1429 (s), 1358 (w), 1292 (w), 1281 (w), 1252 (m), 1238 (m), 1213 (m), 1180 (m), 1163 (m), 1090 (w), 1067 (m), 1024 (m), 1001 (w), 968 (w), 951 (w), 868 (m), 845 (w), 808 (m), 741 (s), 702 (s), 689 (s), 665 (w), 644 (m), 619 cm^−1^ (m); elemental analysis calcd (%) for C_14_H_9_NS_3_: C 58.51, H 3.16, N 4.87, S 33.46; found: C 58.44, H 2.99, N 4.80, S 33.07.

## Conflict of interest

The authors declare no conflict of interest.

## Supporting information

As a service to our authors and readers, this journal provides supporting information supplied by the authors. Such materials are peer reviewed and may be re‐organized for online delivery, but are not copy‐edited or typeset. Technical support issues arising from supporting information (other than missing files) should be addressed to the authors.

Supplementary

## References

[1a] N. Thejo Kalyani , S. J. Dhoble , Renewable Sustainable Energy Rev. 2012, 16, 2696–2723;

[1b] Y. Park , B. Kim , C. Lee , A. Hyun , S. Jang , J.-H. Lee , Y.-S. Gal , T. H. Kim , K.-S. Kim , J. Park , J. Phys. Chem. C 2011, 115, 4843–4850;

[1c] J. K. Salunke , F. L. Wong , K. Feron , S. Manzhos , M. F. Lo , D. Shinde , A. Patil , C. S. Lee , V. A. L. Roy , P. Sonar , P. P. Wadgaonkar , J. Mater. Chem. C 2016, 4, 1009–1018;

[1d] C. Poriel , J. Rault-Berthelot , S. Thiery , C. Quinton , O. Jeannin , U. Biapo , D. Tondelier , B. Geffroy , Chem. Eur. J. 2016, 22, 17930–17935. 27643709 10.1002/chem.201603659

[2a] W. Wu , Y. Liu , D. Zhu , Chem. Soc. Rev. 2010, 39, 1489–1502; 20419204 10.1039/b813123f

[2b] L. Torsi , M. Magliulo , L. Manoli , G. Palazzo , Chem. Soc. Rev. 2013, 42, 8612–8628. 24018860 10.1039/c3cs60127g

[3a] S. Feng , Q.-S. Li , T. A. Niehaus , Z.-S. Li , Org. Electron. 2017, 42, 234–243;

[3b] A. Mishra , M. K. Fischer , P. Bäuerle , Angew. Chem. Int. Ed. 2009, 48, 2474–2499; 10.1002/anie.200804709 19294671

[4] X. Zhao , X. Zhan , Chem. Soc. Rev. 2011, 40, 3728–3743. 21409196 10.1039/c0cs00194e

[5a] M. Hauck , M. Stolte , J. Schönhaber , H.-G. Kuball , T. J. J. Müller , Chem. Eur. J. 2011, 17, 9984–9998; 21780199 10.1002/chem.201100592

[5b] A. W. Franz , S. Stoycheva , M. Himmelhaus , T. J. J. Müller , Beilstein J. Org. Chem. 2010, 6, 72; 20703376 10.3762/bjoc.6.72 PMC2919263

[5c] M. Sailer , A. W. Franz , T. J. J. Müller , Chem. Eur. J. 2008, 14, 2602–2614; 18213672 10.1002/chem.200701341

[5d] H. Tian , X. Yang , R. Chen , Y. Pan , L. Li , A. Hagfeldt , L. Sun , Chem. Commun. 2007, 3741–3743. 10.1039/b707485a 17851613

[6] I. S. Pereţeanu , T. J. J. Müller , Org. Biomol. Chem. 2013, 11, 5127–5135. 23812160 10.1039/c3ob40815a

[7] K. Deuchert , S. Hünig , Angew. Chem. Int. Ed. Engl. 1978, 17, 875–886;

[8] K. Schaper , T. J. J. Müller , Top. Curr. Chem. 2018, 376, 38. 10.1007/s41061-018-0216-1 30221315

[9] For the synthesis, absorption spectra, and cyclic voltammograms of 4-phenyl-4*H*-dithieno[2,3-b:3′,2′-e][1,4]thiazine (**2 a**) and 10-phenyl-10*H*-phenothiazine (**1**), see: C. Dostert , C. Wansrath , W. Frank , T. J. J. Müller , Chem. Commun. 2012, 48, 7271–7273. 10.1039/c2cc32731g 22699424

[10] A. Schneeweis , A. Neidlinger , G. J. Reiss , W. Frank , K. Heinze , T. J. J. Müller , Org. Chem. Front. 2017, 4, 839–846.

[11a] C. Dostert , D. Czajkowski , T. J. J. Müller , Synlett 2014, 25, 371–374;

[11b] C. Dostert , T. J. J. Müller , Org. Chem. Front. 2015, 2, 481–491.

[12] A. P. Schneeweis , S. T. Hauer , G. Reiss , T. J. J. Müller , Chem. Eur. J. 2019, 25, 3582–3590. 30557458 10.1002/chem.201805085

[13] M.-C. Chen , Y.-J. Chiang , C. Kim , Y.-J. Guo , S.-Y. Chen , Y.-J. Liang , Y.-W. Huang , T.-S. Hu , G.-H. Lee , A. Facchetti , T. J. Marks , Chem. Commun. 2009, 1846–1848. 10.1039/b820621j 19319421

[14] L. Levi , T. J. J. Müller , Chem. Soc. Rev. 2016, 45, 2825–2846. 26898222 10.1039/c5cs00805k

[15] F. De Jong , M. J. Janssen , J. Org. Chem. 1971, 36, 1998.

[16a] A. D. Becke , J. Chem. Phys. 1993, 98, 5648–5652;

[16b] A. D. Becke , J. Chem. Phys. 1993, 98, 1372–1377.

[17a] R. Krishnan , J. S. Binkley , R. Seeger , J. A. Pople , J. Chem. Phys. 1980, 72, 650–654;

[17b] A. D. McLean , G. S. Chandler , Chem. Phys. 1980, 72, 5639–5648.

[18] Gaussian 09, Revision A.02, M. J. Frisch, G. W. Trucks, H. B. Schlegel, G. E. Scuseria, M. A. Robb, J. R. Cheeseman, G. Scalmani, V. Barone, B. Mennucci, G. A. Petersson, H. Nakatsuji, M. Caricato, X. Li, H. P. Hratchian, A. F. Izmaylov, J. Bloino, G. Zheng, J. L. Sonnenberg, M. Hada, M. Ehara, K. Toyota, R. Fukuda, J. Hasegawa, M. Ishida, T. Nakajima, Y. Honda, O. Kitao, H. Nakai, T. Vreven, J. A. Montgomery, Jr., J. E. Peralta, F. Ogliaro, M. Bearpark, J. J. Heyd, E. Brothers, K. N. Kudin, V. N. Staroverov, R. Kobayashi, J. Normand, K. Raghavachari, A. Rendell, J. C. Burant, S. S. Iyengar, J. Tomasi, M. Cossi, N. Rega, J. M. Millam, M. Klene, J. E. Knox, J. B. Cross, V. Bakken, C. Adamo, J. Jaramillo, R. Gomperts, R. E. Stratmann, O. Yazyev, A. J. Austin, R. Cammi, C. Pomelli, J. W. Ochterski, R. L. Martin, K. Morokuma, V. G. Zakrzewski, G. A. Voth, P. Salvador, J. J. Dannenberg, S. Dapprich, A. D. Daniels, O. Farkas, J. B. Foresman, J. V. Ortiz, J. Cioslowski, D. J. Fox, Gaussian, Inc., Wallingford CT, **2009**.

[19a] R. Bauernschmitt , R. Ahlrichs , Chem. Phys. Lett. 1996, 256, 454–464;

[19b] M. E. Casida , C. Jamorski , K. C. Casida , D. R. Salahub , J. Chem. Phys. 1998, 108, 4439–4449;

[19c] R. E. Stratmann , G. E. Scuseria , M. J. Frisch , J. Chem. Phys. 1998, 109, 8218–8224.

[20a] G. Scalmani , M. J. Frisch , J. Chem. Phys. 2010, 132, 114110–114115; 20331284 10.1063/1.3359469

[20b] A. V. Marenich , C. J. Cramer , D. G. Truhlar , J. Phys. Chem. B 2009, 113, 6378–6396. 19366259 10.1021/jp810292n

[21] L. Michaelis , Chem. Rev. 1935, 16, 243–286.

[22] P. Borowicz , J. Herbich , A. Kapturkiewicz , R. Anulewicz-Ostrowska , J. Nowacki , G. Grampp , Phys. Chem. Chem. Phys. 2000, 2, 4275–4280.

[23] J. Li , C. L. Fischer , J. L. Chen , D. Bashford , Inorg. Chem. 1996, 35, 4694–4702.

[24] Bond orders were extracted from the Gaussian 09 calculation outputs by the help of the Multiwfn software: T. Lu , F. Chen , J. Comput. Chem. 2012, 33, 580–592. 22162017 10.1002/jcc.22885

[25] Determined relative to Coumarine 153 in ethanol (*Φ* _f_=0.38): G. Jones , W. R. Jackson , C. Y. Choi , W. R. Bergmark , J. Phys. Chem. 1985, 89, 294–300.

[26] S. Fery-Forgues , D. Lavabre , J. Chem. Educ. 1999, 76, 1260–1264.

[27] A. Bejan , S. Shova , M.-D. Damaceanu , B. C. Simionescu , L. Marin , Cryst. Growth Des. 2016, 16, 3716–3730.

[28] L. Yang , J.-K. Feng , A.-M. Ren , J. Org. Chem. 2005, 70, 5987–5996. 16018695 10.1021/jo050665p

