# Supplementary material for: Electron‐Rich Phenothiazine Congeners and Beyond: Synthesis and Electronic Properties of Isomeric Dithieno[1,4]thiazines
Source: Chemistry. 2020 Aug 31;26(53):12111–8. doi: 10.1002/chem.202000137 (PMC7540341; doi:10.1002/chem.202000137)
Supplement: Supplementary file 1 — Supplementary [file CHEM-26-12111-s001.pdf]

Chemistry–A European Journal

Supporting Information

Electron-Rich Phenothiazine Congeners and Beyond: Synthesis and Electronic Properties of Isomeric Dithieno[1,4]thiazines

Lars May and Thomas J. J. Müller*[a]

Table of Contents

1 General Considerations....1

2 Syntheses....2

2.1 One-pot synthesis of bis(4-bromothiophen-3-yl)sulfane (**3a**)....2

2.2 General procedure 1 (**GP1**) for the one-pot synthesis of dithienyl sulfides **3b** and **3c** ...3

2.2.1 Di(thiophen-3-yl)sulfane (**3b**)....4

2.2.2 Bis(4-bromothiophen-3-yl)sulfane (**3c**)....5

2.3 One-pot synthesis of bis(3-bromothiophen-2-yl)sulfane (**3d**)....5

2.4 Bis(2-iodothiophen-3-yl)sulfane (**3e**)....6

2.5 General procedure 2 (**GP2**) for the preparation of dithieno[1,4]thiazines **2**....7

2.5.1 4-Phenyl-4*H*-dithieno[2,3-b:3',4'-e][1,4]thiazine (**2b**)....8

2.5.2 8-Phenyl-8*H*-dithieno[3,4-b:3',4'-e][1,4]thiazine (**2c**)....8

2.5.3 8-Phenyl-8*H*-dithieno[3,2-b:2',3'-e][1,4]thiazine (**2d**)....9

3 ^{1}H and ^{13}C NMR spectra....10

3.1 Bis(4-bromothiophen-3-yl)sulfane (**3a**)....10

3.2 Di(thiophen-3-yl)sulfane (**3b**)....11

3.3 Bis(4-bromothiophen-3-yl)sulfane (**3c**)....12

3.4 Bis(3-bromothiophen-2-yl)sulfane (**3d**)....13

3.5 Bis(2-iodothiophen-3-yl)sulfane (**3e**)....14

3.6 4-Phenyl-4*H*-dithieno[2,3-b:3',4'-e][1,4]thiazine (**2b**)....15

3.7 8-Phenyl-8*H*-dithieno[3,4-b:3',4'-e][1,4]thiazine (**2c**)....16

3.8 8-Phenyl-8*H*-dithieno[3,2-b:2',3'-e][1,4]thiazine (**2d**)....17

4 Cyclovoltammetric Data....18

5 Absorption and Emission Spectra of Compounds **2**....19

6 Data of Quantum Chemical Calculations....21

6.1 Computed xyz-coordinates, excitations of compounds **1** and **2** and selected properties derived from the DFT calculations....21

6.1.1.1 Computed xyz coordinates of compound **2a** (B3LYP/6-311G* PCM CH_2Cl_2)....23

6.1.1.2 Computed excitations of compound **2a** (B3LYP/6-311G* PCM CH_2Cl_2) 24

6.1.1.3 Computed xyz coordinates of extra conformation of compound **2a** (B3LYP/6-311G* PCM CH_2Cl_2) 25

6.1.1.4 Computed xyz coordinates of radical cation **2a**$^{+}$ of compound **2a** (uB3LYP/6-311G* PCM CH_2Cl_2) 27

6.1.1.5 Reoptimization of compound **2a** (uB3LYP/6-311G*) 28

6.1.2.1 Computed xyz coordinates of compound **2b** (B3LYP/6-311G* PCM CH_2Cl_2) 29

6.1.2.2 Computed excitations of compound **2b** (B3LYP/6-311G* PCM CH_2Cl_2) 31

6.1.2.3 Computed xyz coordinates of extra conformation of compound **2b** (B3LYP/6-311G* PCM CH_2Cl_2) 32

6.1.2.4 Computed xyz coordinates of radical cation **2b**$^{+}$ of compound **2b** (uB3LYP/6-311G* PCM CH_2Cl_2) 34

6.1.2.5 Reoptimization of compound **2b** (uB3LYP/6-311G*) 35

6.1.3.1 Computed xyz coordinates of compound **2c** (B3LYP/6-311G* PCM CH_2Cl_2) 36

6.1.3.2 Computed excitations of compound **2c** (B3LYP/6-311G* PCM CH_2Cl_2) 38

6.1.3.3 Computed xyz coordinates of extra conformation of compound **2c** (B3LYP/6-311G* PCM CH_2Cl_2) 39

6.1.3.4 Computed xyz coordinates of radical cation **2c**$^{+}$ of compound **2c** (uB3LYP/6-311G* PCM CH_2Cl_2) 41

6.1.3.5 Reoptimization of compound **2c** (uB3LYP/6-311G*) 42

6.1.4.1 Computed xyz coordinates of compound **2d** (B3LYP/6-311G* PCM CH_2Cl_2) 43

6.1.4.2 Computed excitations of compound **2d** (B3LYP/6-311G* PCM CH_2Cl_2) 45

6.1.4.3 Computed xyz coordinates of S_1 of compound **2d** (B3LYP/6-311G* PCM CH_2Cl_2)46

6.1.4.4 Computed excitations of S_1 (emission of S_1) of compound **2d** (B3LYP/6-311G* PCM CH_2Cl_2) 48

6.1.4.5 Computed xyz coordinates of extra conformation of compound **2d** (B3LYP/6-311G* PCM CH_2Cl_2) 48

6.1.4.6 Computed excitations of extra conformation of compound **2d** (B3LYP/6-311G* PCM CH_2Cl_2) 49

6.1.4.7 Computed xyz coordinates of radical cation **2d**$^{+}$ of compound **2d** (uB3LYP/6-311G* PCM CH_2Cl_2) 51

6.1.4.8 Reoptimization of compound **2d** (uB3LYP/6-311G*) 53

6.1.5.1 Computed xyz coordinates of compound **1** (B3LYP/6-311G* PCM CH_2Cl_2) 54

6.1.5.2 Computed excitations of compound **1** (B3LYP/6-311G* PCM CH_2Cl_2) 55

6.1.5.3 Computed xyz coordinates of extra conformation of compound **1** (B3LYP/6-311G* PCM CH_2Cl_2) 57

6.1.5.4 Computed xyz coordinates of radical cation **1+** of compound **1** (uB3LYP/6-311G* PCM CH2Cl2) 59

6.1.5.5 Reoptimization of compound **1** (uB3LYP/6-311G*) 61

6.1.6.1 Computed xyz coordinates of compound **2e** (B3LYP/6-311G* PCM CH_2Cl_2) 62

6.1.6.2 Computed xyz coordinates of radical cation **2e**$^+$ of compound **2e** (uB3LYP/6-311G* PCM CH_2Cl_2) 63

6.1.6.3 Reoptimization of compound **2e** (uB3LYP/6-311G*) 65

6.1.7.1 Computed xyz coordinates of compound **2f** (B3LYP/6-311G* PCM CH_2Cl_2) 66

6.1.7.2 Computed xyz coordinates of radical cation **2f**$^+$ of compound **2f** (uB3LYP/6-311G* PCM CH_2Cl_2) 67

6.1.7.3 Reoptimization of compound **2f** (uB3LYP/6-311G*) 68

6.2 DFT calculation of the redox potentials of compounds **1** and **2** 70

7 References 73

1 General Considerations

All reactions were carried out in flame-dried Schlenk tubes by using syringes under nitrogen atmosphere. Dry solvents for reactions and analytics were directly used from a MB-SPS 800 solvent drying system (MBraun) except of toluene, which was refluxed under nitrogen atmosphere over sodium, distilled and stored in a Schlenk flask over molecular sieve 4 Å under nitrogen atmosphere. 10-Phenyl-10*H*-phenothiazine (**1**)[1] and 4-phenyl-4*H*-dithieno[2,3-b:3',2'-e][1,4]thiazine (**2a**)[2] and were synthesized according to the literature procedures as indicated. Commercial grade reagents were purchased from Sigma Aldrich, Alfa Aesar, ABCR, Fluorochem and ACROS and used as supplied without further purification. Crude mixtures were adsorbed on Celite® 545 (0.02-0.20 mm) from Carl Roth GmbH Co.KG. The purification of products was performed on silica gel 60 M (0.04–0.063 mm) from Macherey-Nagel by using the flash technique under a pressure of 2 bar. For TLC silica gel coated aluminium plates (60, F_{254}) from Merck were employed and analyzed with UV light at 254 or 365 nm.

^{1}H, ^{13}C, and 135-DEPT NMR spectra were recorded at 293 K on 300 MHz (Bruker AVIII 300), 500 MHz (Bruker Avance DRX 500) and the resonances of the residues of non-deuterated $CDCl_3$ (^{1}H δ = 7.26 ppm, ^{13}C δ = 77.00 ppm), acetone-d_6 (^{1}H δ = 2.05 ppm, ^{13}C δ = 29.84 ppm) or THF-d_8 (^{1}H δ = 3.58 ppm, ^{13}C δ = 67.57 ppm) were locked as internal standards. The multiplicities of signals are abbreviated as follows: d = doublet, dd = doublet of doublets and m = multiplet. The assignments of C_{quat} and CH nuclei are based on DEPT spectra.

IR spectra were recorded on a Shimadzu IR Affinity-1 with ATR technique. The intensities of IR signals are abbreviated as s (strong), m (medium) and w (weak).

EI mass spectra were recorded on Triple-Quadrupole mass spectrometer TSQ 7000 (Finnigan MAT). MALDI-TOF mass spectra were measured on an UltrafleXtreme apparatus (Bruker Daltonics).

The elemental analyses were carried out on a Perkin Elmer Series II Analyser 2400 at the Institute for Pharmaceutical and Medicinal Chemistry at Heinrich-Heine-University Düsseldorf.

Melting points (uncorrected) were measured with a Büchi B545 apparatus.

Absorption spectra were recorded in dichloromethane high performance liquid chromato-graphy (HPLC) grade at 293 K on Perkin Elmer UV/vis/NIR Lambda 19 spectrometer. For the determination of the extinction coefficients ε absorption measurements at five different concentrations were carried out. Emission spectra were recorded in dichloromethane HPLC grade at 293 K on a Perkin Elmer LS55 spectrometer.

Quantum chemical calculations were carried out utilizing the HPC-Cluster Ivybridge of the

Zentrum for Informations- und Medientechnologie (ZIM) at the Heinrich-Heine-University Düsseldorf.

Cyclic voltammetry experiments (EG&G Princeton Applied Research Model 263A potentiostat) were performed under argon atmosphere in dry and degassed dichloromethane at 293 K using *n*-Bu_4NPF_6 (0.1 M) as electrolyte and at scan rates v of 100, 250, 500 and 1000 mVs^{-1}. The three-electrode array consists of a working electrode with a 2 mm platinum disk, a platinum wire counter electrode, and an Ag/AgCl (3.0 M NaCl) reference electrode. The potentials were corrected by adding the internal standard decamethylferrocene to each measurement. Decamethylferrocene was referenced to the internal redox standard ferrocene ($E_0^{0/+1}$(deca-methylferrocene) = -95 mV vs. $E_0^{0/+1}$(ferrocene) = 450 mV).[3] Therefore the outlined potentials are indirectly referenced to ferrocene.

2 Syntheses

2.1 One-pot synthesis of bis(4-bromothiophen-3-yl)sulfane (3a)[4]

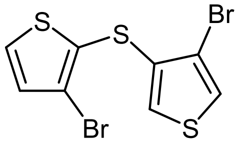

In a flame-dried Schlenk vessel under nitrogen atmosphere were filled 829 mg (3.43 mmol, 1.00 equiv) 3,4-dibromothiophene (**5b**) and 4 mL dry diethyl ether and were cooled down to -78 °C (isopropanol/dry ice bath). Then, 2.14 mL (3.43 mmol, 1.00 equiv, 1.6 M in hexane) *n*-butyllithium was added dropwise slowly and the reaction solution was stirred for 15 min at -78 °C. The temperature was raised to 0 °C (water/ice bath) and the volatiles were removed in vacuo carefully (approx. 1 h). The remaining colorless solid was dissolved in 10 mL dry diethyl ether and was cooled down to -78 °C again. Next, 110 mg (3.43 mmol, 1.00 equiv) fine mortared sulfur was added and the stirring was continued for 15 min at -78 °C. Afterwards, while the temperature was raised to 0 °C, the stirring was continued for another 15 min. In the next step, 654 mg (3.43 mmol, 1.00 equiv) *p*-toluenesulfonyl chloride was added slowly. It was stirred vigorously for 10 min at 0 °C and then for 2 h at 40 °C. Simultaneously, 580 µL (4.12 mmol, 1.20 equivs) diisopropyl amine and 4 mL dry diethyl ether were charged into another flame-dried Schlenk vessel under nitrogen atmosphere and were cooled down to 0 °C. Then, 2.58 mL (4.12 mmol, 1.20 equivs, 1.6 M in hexane) *n*-butyllithium was added dropwise slowly to the diisopropyl amine solution. It was stirred for 10 min at 0 °C and for 10 min at ambient temperature after that. In the meantime, 671 mg (4.12 mmol, 1.20 equivs) 3-bromothiophene (**5a**) and 4 mL dry diethyl were filled into a third flame-dried Schlenk vessel under nitrogen atmosphere and were cooled down to -78 °C. The previously prepared lithium diisopropylamide solution was dropped into the 3-bromo-

thiophene solution and it was stirred at -78 °C for 30 min. This lithiated 3-bromothiophene was added to the vigorously stirred reaction solution, which had been cooled down to -78 °C before. Then, the reaction solution was stirred at -78 °C for 1.5 h. Finally, the reaction was quenched by the addition of 10 mL water. The organic layer was separated, the aqueous layer was extracted with diethyl ether three times and the combined organic layers were died with dry magnesium sulfate. The volatiles were removed by evaporation and the crude product was purified chromatographically on silica gel (*n*-hexane) to give 488 mg (1.37 mmol, 40%) of **3a** in form of a light-yellow oil.

R_f (*n*-hexane) = 0.50. ^{1}H NMR (300 MHz, acetone-d$_6$): δ 7.18 (d, $^{3}J_{HH}$ = 5.56 Hz, 1H), 7.39 (d, $^{5}J_{HH}$ = 3.44 Hz, 1H), 7.70 (d, $^{5}J_{HH}$ = 3.44 Hz, 1H), 7.75 (d, $^{3}J_{HH}$ = 5.59 Hz, 1H). ^{13}C NMR (75 MHz, Acetone-d$_6$): δ 113.1 (C_{quat}), 118.6 (C_{quat}), 126.3 (CH), 128.1 (CH), 131.8 (C_{quat}), 131.95 (CH), 132.03 (CH), 132.2 (C_{quat}). MS(EI) *m*/*z* (%): 358 ([^{81}Br^{81}Br-M]$^+$, 16), 356 ([^{79}Br^{81}Br-M]$^+$, 27), 354 ([^{79}Br ^{79}Br-M]$^+$,15), 198 (14), 197 (13), 196 ([M – 2 Br]$^+$, 100), 152 (10), 114 (14), 98 (12), 82 (22), 81 (13), 69 (27), 45 (12). IR: $\tilde{\nu}$ [cm^{-1}] = 3103 (w), 2922 (w), 2851 (w), 1699 (w), 1485 (m), 1472 (m), 1385 (w), 1342 (m), 1319 (m), 1246 (w), 1152 (m), 1113 (w), 1086 (w), 1024 (w), 1011 (w), 920 (m), 858 (s), 791 (s), 777 (s), 716 (s), 667 (m), 635 (w).

2.2 General procedure 1 (GP1) for the one-pot synthesis of dithienyl sulfides 3b and 3c

In a flame-dried Schlenk vessel under nitrogen atmosphere were filled 1.00 equiv of a bromo thiophene **5** and 0.4 mL/mmol dry diethyl ether. The reaction solution was cooled down to -78 °C (isopropanol/dry ice bath). Then, 1.00 equiv *n*-butyllithium (1.6 M in hexane) was added dropwise slowly and the reaction solution was stirred for 30 min at -78 °C. The temperature was raised to 0 °C (water/ice bath) and the volatiles were removed in vacuo carefully (approx. 1 h). The remaining colorless to light yellow solid was dissolved in 0.4 mL/mmol dry diethyl ether and cooled down to -78 °C again. Next, 1.00 equiv fine mortared sulfur was added. The reaction solution had been stirred for 30 min at -78 °C and for 30 min at 0 °C afterwards. To the reaction solution was added 1.00 equivs *p*-toluenesulfonyl chloride slowly. It was stirred for 30 min at 0 °C and then for 3 h at 40 °C. Simultaneously, another portion of a bromo thiophene **5** was lithiated: In a flame-dried Schlenk vessel under nitrogen atmosphere were filled 1.20 equivs of bromo thiophene **5** and 0.4 mL/mmol dry diethyl ether. The reaction solution was cooled down to -78 °C. Then, 1.20 equivs *n*-butyllithium (1.6 M in hexane) were added dropwise slowly and the reaction solution was stirred for 30 min at -78 °C. Likewise, the volatiles were removed and the remaining colorless to light yellow solid was dissolved in 0.4 mL/mmol dry diethyl ether and

cooled down to -78 °C again. The resulting second portion of thienyl lithium was dropped to the reaction solution, which had been cooled down to -78 °C after the completion of the tosylation. The reaction solution was stirred overnight and was allowed to come to ambient temperature slowly meanwhile. The reaction was quenched by the addition of 50 mL water. The organic layer was separated, the aqueous layer was extracted with diethyl ether three times and the combined organic layers were died with dry magnesium sulfate. The volatiles were removed by evaporation and the crude product was purified by column chromatography. For experimental details see table 1.

Table 1. Experimental details **GP 1**.

Bromo thiophene **5** [g] (mmol)	*n*-BuLi [c] [mL] (mmol)	S_8 [g] (mmol)	*p*TsCl [g] (mmol)	Yield **3** [g] (%)
3-Bromo thiophene (**5a**)				**3b**
3.261 (20.00) [a]	12.50 (20.00) [a]	0.641 (20.00)	3.813 (20.00)	3.018 (76)
3.913 (24.00) [b]	15.00 (24.00) [b]			
3,4-Dibromo thiophene (**5b**)				**3c**
2.42 (10.0) [a]	6.26 (10.0) [a]	0.321 (10.0)	1.90 (10.0)	5.61 (56)
2.95 (12.2) [b]	7.62 (12.2) [b]			

a: 1st portion; b: 2nd portion; c: 1.6 M in hexane).

2.2.1 Di(thiophen-3-yl)sulfane (3b)[4]

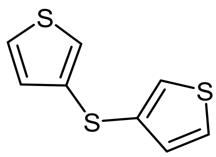

The crude product was synthesized following **GP1** and purified chromatographically on silica gel (*n*-hexane) to give 3.018 g (15.22 mmol, 76 %) of **3b** in form of a light-yellow oil.

R_f (*n*-hexane) = 0.44. ^{1}H NMR (300 MHz, $CDCl_3$): δ 7.00 (dd, $^3J_{HH}$ = 5.03 Hz, $^5J_{HH}$ = 1.28 Hz, 2H), 7.19 (dd, $^4J_{HH}$ = 3.05 Hz, $^5J_{HH}$ = 1.30 Hz, 2H), 7.32 (dd, $^3J_{HH}$ = 5.01 Hz, $^4J_{HH}$ = 3.04 Hz, 2H). ^{13}C NMR (75 MHz, $CDCl_3$): δ 124.9 (CH), 126.6 (CH), 129.9 (CH), 131.6 (C_{quat}). MS (EI) *m/z* (%): 199 ($[M + H]^+$, 13), 198 ($[M]^+$, 100), 167 (13), 166 (10), 165 (13), 153 (48), 134 (10), 121 (19), 115 ($[M – C_4H_3S]^+$, 7), 71 (14). IR: $\tilde{\nu}$ [cm^{-1}] = 1350 (m), 1196 (m), 1096 (m), 891 (m), 851 (m), 758 (s), 689 (m), 610 (m).

2.2.2 Bis(4-bromothiophen-3-yl)sulfane (3c)[4]

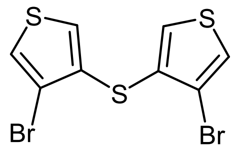

The crude product was synthesized following **GP1** and purified chromatographically on silica gel (*n*-hexane) to give 1.998 g (5.610 mmol, 56 %) of **3c** in form of a colorless oil.

R_f (*n*-hexane) = 0.43. ^{1}H NMR (300 MHz, acetone-d$_6$): δ 7.41 (d, $^5J_{HH}$ = 3.43 Hz, 2H), 7.71 (d, $^5J_{HH}$ = 3.45 Hz, 2H). ^{13}C NMR (75 MHz, acetone-d$_6$): δ 114.0 (C_{quat}), 126.2 (CH), 128.7 (CH), 131.7 (C_{quat}). MS (EI) *m/z* (%): 358 ([2^{81}Br-M]$^+$, 9), 356 ([^{79}Br^{81}Br-M]$^+$, 16), 354 ([2^{79}Br-M]$^+$, 8), 277 ([^{81}Br-M – Br]$^+$, 6), 275 ([^{79}Br-M – Br]$^+$, 5), 198 (13), 197 (15), 196 ([M – 2 Br]$^+$, 100), 98 (13), 82 (16), 81 (11), 69 (20). IR: $\tilde{\nu}$ [cm^{-1}] = 3103 (w), 1472 (m), 1454 (w), 1315 (s), 1260 (w), 1229 (w), 1198 (w), 1155 (w), 1111 (w), 1092 (w), 1064 (w), 1040 (w), 1015 (w), 920 (m), 880 (w), 849 (m), 791 (s), 773 (s), 746 (w), 719 (w), 698 (w), 658 (m), 635 (w).

2.3 One-pot synthesis of bis(3-bromothiophen-2-yl)sulfane (3d)[4]

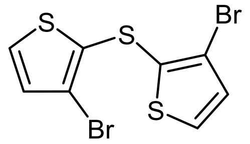

In a flame-dried Schlenk vessel under nitrogen atmosphere were filled 2.30 mL (16.3 mmol, 1.00 equiv) diisopropyl amine and 8 mL dry diethyl ether and cooled down to 0 °C (water/ice bath). Then, 10.2 mL (16.3 mmol, 1.00 equiv, 1.6 M in hexane) *n*-butyllithium was added dropwise slowly and the reaction solution was stirred for 10 min at 0 °C and for 10 min at ambient temperature after that. In the meantime, 2.65 g (16.3 mmol, 1.00 equiv) 3-bromothiophene (**5a**) and 16 mL dry diethyl ether were charged into another flame-dried Schlenk vessel under nitrogen atmosphere and cooled down to -78 °C (isopropanol/dry ice bath). The previously prepared lithium diisopropylamide solution was dropped into the 3-bromothiophene solution. The reaction solution was stirred at -78 °C for 1 h. Next, 521 mg (16.3 mmol, 1.00 equiv) fine mortared sulfur was added and the stirring was continued for 15 min at -78 °C. While the temperature was raised to 0 °C, the stirring was continued for 1 h. In the next step, 3.10 g (16.3 mmol, 1.00 equiv) *p*-toluenesulfonyl chloride was added slowly. It was stirred vigorously for 15 min at 0 °C and then for 2 h at 40 °C. Simultaneously, another portion of 3-bromothiophene (**5a**) was lithiated as before: In a third flame-dried Schlenk vessel under nitrogen atmosphere were filled 2.70 mL (19.1 mmol, 1.00 equiv)

diisopropyl amine and 9 mL dry diethyl ether and cooled down to 0 °C. Then, 11.9 mL (19.0 mmol, 1.00 equiv, 1.6 M in hexane) *n*-butyllithium was added dropwise slowly. It was stirred for 10 min at 0 °C and for 10 min at ambient temperature after that. In the meantime, 3.12 g (19.1 mmol, 1.20 equivs) 3-bromothiophene (**5a**) and 19 mL dry diethyl ether were charged into a fourth flame-dried Schlenk vessel under nitrogen atmosphere and cooled down to -78 °C. The lithium diisopropylamide solution was dropped into the 3-bromo-thiophene solution and it was stirred at -78 °C for 1 h. This second portion of lithiated 3-bromothiophene was added to the vigorously stirred reaction solution, which had been cooled down to -78 °C before. The reaction solution was stirred at -78 °C for 2 h. Finally, the reaction was quenched by the addition of 25 mL water. The organic layer was separated, the aqueous layer was extracted with diethyl ether three times and the combined organic layers were died with dry magnesium sulfate. The volatiles were removed by evaporation and the crude product was purified chromatographically on silica gel (*n*-hexane) and by recrystallization from *n*-hexane to give 2.56 g (8.30 mmol, 51%) of **3d** in form of a colorless solid.

Mp.: 55–56 °C, R_f (*n*-hexane) = 0.30. ^{1}H NMR (300 MHz, $CDCl_3$): δ 7.00 (d, $^3J_{HH}$ = 5.54 Hz, 2H), 7.34 (d, $^3J_{HH}$ = 5.55 Hz, 2H). ^{13}C NMR (75 MHz, $CDCl_3$): δ 118.1 (C_{quat}), 129.99 (CH), 130.0 (C_{quat}), 131.0 (CH). MS(EI) *m/z* (%): 358 ([^{81}Br^{81}Br-M]$^+$, 14), 356 ([^{79}Br^{81}Br-M]$^+$, 24), 354 ([^{79}Br^{79}Br-M]$^+$,11), 198 (14), 196 ([M – 2 Br]$^+$, 100). IR: $\tilde{\nu}$ [cm^{-1}] = 3094 (w), 3080 (w), 2922 (w), 1776 (w), 1732 (w), 1607 (w), 1582 (w), 1562 (w), 1512 (w), 1479 (w), 1445 (w), 1379 (m), 1341 (m), 1238 (w), 1175 (w), 1153 (m), 1084 (m), 1024 (w), 986 (w), 883 (w), 856 (s), 812 (w), 799 (w), 758 (w), 733 (s), 716 (s), 694 (m), 673 (w), 664 (w), 648 (w).

2.4 Bis(2-iodothiophen-3-yl)sulfane (3e)

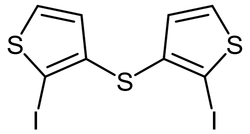

In a Schlenk vessel under nitrogen atmosphere were charged 191 mg (0.960 mmol, 1.00 equiv) di(thiophen-3-yl)sulfane (**5b**) and 6 mL dry DMF and cooled down to 0 °C (water/ice bath). Then, 432 mg (1.92 mmol, 2.00 equivs) *N*-iodosuccinimide was added in one portion. The reaction solution was stirred for 46 h, while the temperature was raised to ambient temperature slowly by thawing. The reaction was quenched by the addition of 10 mL of a saturated sodium sulfite solution. The organic layer was separated, the aqueous layer was extracted with diethyl ether three times and the combined organic layers were died with dry magnesium sulfate. The volatiles were removed by evaporation and the crude product

was purified chromatographically on silica gel (*n*-hexane) to give 371 mg (0.820 mmol, 86%) of **3e** in form of a light-yellow oil.

R_f (*n*-hexane) = 0.46. ^{1}H NMR (300 MHz, $CDCl_3$): δ 6.71 (d, $^3J_{HH}$ = 5.51 Hz, 2H), 7.40 (d, $^3J_{HH}$ = 5.52 Hz, 2H). ^{13}C NMR (75 MHz, $CDCl_3$): δ 80.4 (C_{quat}), 130.3 (CH), 131.5 (CH), 137.6 (C_{quat}). MS (EI) *m/z* (%): 450 ($[M]^+$, 17), 323 ($[M - I]^+$, 1), 298 (11), 270 (100), 242 (29), 241 ($[M - C_4HIS]^+$, 11), 198 (10), 197 (19), 196 ($[M - 2\ I]^+$, 55), 153 (10), 128 (17), 116 (19), 115 (61), 114 ($[M - C_4H_2I_2S]^+$, 18), 82 ($[M - C_4H_2I_2S_2]^+$, 5), 71 (14), 69 (12), 45 (10). IR: $\tilde{\nu}$ [cm^{-1}] = 1485 (m), 1369 (m), 1333 (m), 1150 (m), 868 (s), 804 (m), 702 (s), 648 (m), 615 (m). Anal. calcd. for $C_8H_4I_2S_3$: C 21.35., H 0.90, S 21.37; Found: C 21.48, H 0.76, S 21.55.

2.5 General procedure 2 (GP2) for the preparation of dithieno[1,4]thiazines 2

In a flame-dried Schlenk vessel under nitrogen atmosphere were charged 1.00 equiv dithienyl sulfide **3**, 1.20 equivs aniline (**6**), 3.00 equivs sodium *tert*-butoxide, 7.5 mol% bis(dibenzylideneacetone)palladium(0), 15 mol% 1,1′-bis(diphenylphosphino)ferrocene and 6 mL/mmol dry toluene. After degassing with nitrogen for 5 min, the reaction solution was stirred at 100 °C until full conversion of the dithienyl sulfide **3** was observed via TLC. The volatiles were removed by evaporation and the crude product was purified by column chromatography and by recrystallization. For experimental details see table 2.

Table 2. Experimental details **GP2**.

**3** [mg] (mmol)	Aniline (**6**) [mg] (mmol)	$Pd(dba)_2$/dppf [mg] (µmol)	NaO^{t}Bu [mg] (mmol)	*t* [h]	Dithienothiazine **3** yield [mg] (%)
**3a** 429 (1.20)	135 (1.45)	52.0 (90.0) / 100 (180)	346 (3.60)	19	**2b** 245 (71)
**3c** 331 (0.930)	354 (3.80)	40.0 (70.0) / 78.0 (140)	268 (2.79)	20	**2c** 58.0 (22)
**3a** 1427 (3.17)	354 (3.80)	137 (238) / 263 (476)	914 (9.51)	18	**2d** 352 (38)

2.5.1 4-Phenyl-4*H*-dithieno[2,3-b:3',4'-e][1,4]thiazine (2b)

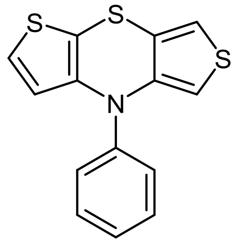

The crude product was synthesized following **GP2** and purified chromatographically on silica gel (*n*-hexane with 1% triethyl amine) and by recrystallization from *n*-hexane to give 245 mg (0.850 mmol, 71%) of **2b** in form of a beige solid.

Mp.: 116–119 °C. R_f (*n*-hexane) = 0.35. ^{1}H NMR (300 MHz, acetone-d$_6$): δ 5.66 (d, $^5J_{HH}$ = 3.27 Hz, 1H), 6.01 (d, $^3J_{HH}$ = 5.50 Hz, 1H), 6.99 (d, $^5J_{HH}$ = 3.28 Hz, 1H), 7.14 (d, $^3J_{HH}$ = 5.50 Hz, 1H), 7.42 - 7.47 (m, 2H), 7.47 - 7.53 (m, 1H), 7.58 - 7.66 (m, 2H). ^{13}C NMR (125 MHz, acetone-d$_6$): δ 101.5 (CH), 117.7 (CH), 117.9 (C$_{quat}$), 119.9 (CH), 123.3 (CH), 129.2 (CH), 129.9 (C$_{quat}$), 130.0 (CH), 131.5 (CH), 142.3 (C$_{quat}$), 143.7 (C$_{quat}$), 144.0 (C$_{quat}$). MS(MALDI-TOF) *m*/*z*: 287.678 ([M]$^+$). IR: $\tilde{\nu}$ [cm^{-1}] = 3107 (w), 1595 (w), 1553 (m), 1533 (s), 1493 (m), 1439 (m), 1391 (m), 1379 (m), 1366 (m), 1307 (w), 1277 (m), 1265 (m), 1099 (m), 1070 (w), 1051 (w), 1030 (m), 1020 (w), 1001 (w), 880 (w), 856 (m), 829 (w), 799 (m), 760 (s), 738 (s), 685 (s), 644 (m). Anal. calcd. for $C_{14}H_9NS_3$: C 58.51., H 3.16, N 4.87, S 33.46; Found: C 58.58, H 3.07, N 4.83, S 33.17.

2.5.2 8-Phenyl-8*H*-dithieno[3,4-b:3',4'-e][1,4]thiazine (2c)

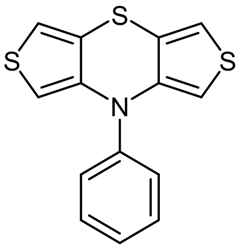

The crude product was synthesized following **GP2** and purified chromatographically on silica gel (*n*-hexane) and by recrystallization from *n*-hexane to give 58 mg (0.20 mmol, 22%) of **2c** in form of a colorless needles.

Mp.: 136-138 °C. R_f (*n*-hexane) = 0.31. ^{1}H NMR (300 MHz, acetone-d$_6$): δ 5.60 (d, $^5J_{HH}$ = 3.35 Hz, 2H), 7.04 (d, $^5J_{HH}$ = 3.32 Hz, 2H), 7.46 - 7.51 (m, 2H), 7.51 - 7.57 (m, 1H), 7.62 - 7.70 (m, 2H). ^{13}C NMR (75 MHz, acetone-d$_6$): δ 100.3 (CH), 117.3 (CH), 117.6 (C$_{quat}$), 129.5 (CH), 130.1 (CH), 131.7 (CH), 142.6 (C$_{quat}$), 143.4 (C$_{quat}$). MS(MALDI-TOF) *m*/*z*: 286.924

([M]$^+$). IR: $\tilde{\nu}$ [cm^{-1}] = 3098 (w), 2972 (w), 2886 (w), 1595 (w), 1522 (m), 1514 (m), 1489 (m), 1449 (m), 1425 (s), 1379 (m), 1369 (m), 1342 (s), 1273 (m), 1250 (m), 1179 (m), 1161 (m), 1152 (w), 1067 (m), 1038 (m), 1001 (w), 928 (w), 895 (m), 881 (w), 858 m, 835 (w), 822 (m), 748 (s), 698 (s), 691 (m), 677 (m), 622 (m), 610 (s). Anal. calcd. for $C_{14}H_9NS_3$: C 58.51, H 3.16, N 4.87, S 33.46; Found.: C 58.62, H 3.08, N 4.76, S 33.23.

2.5.3 8-Phenyl-8*H*-dithieno[3,2-b:2',3'-e][1,4]thiazine (2d)

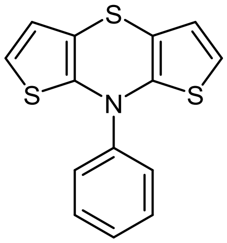

The crude product was synthesized following **GP2** and purified chromatographically on silica gel (*n*-hexane with 1% triethyl amine) and by recrystallization from ethanol to give 352 mg (1.22 mmol, 38%) of **2d** in form of a yellow solid.

Mp.: 97–99 °C. R_f (*n*-hexane) = 0.35. ^{1}H NMR (300 MHz, acetone-d$_6$): δ 6.59 (d, $^3J_{HH}$ = 5.62 Hz, 2H), 6.93 (d, $^3J_{HH}$ = 5.62 Hz, 2H), 7.31 - 7.34 (m, 1H), 7.54 - 7.57 (m, 4H). ^{13}C NMR (125 MHz, THF-d$_8$): δ 112.6 (C_{quat}), 117.6 (CH), 125.2 (CH), 127.2 (CH), 128.4 (CH), 130.9 (CH), 144.0 (C_{quat}), 146.4 (C_{quat}). MS(MALDI-TOF) *m*/*z*: 287.414 ([M]$^+$). IR: $\tilde{\nu}$ [cm^{-1}] = 1722 (w), 1582 (m), 1537 (w), 1512 (w), 1458 (m), 1429 (s), 1358 (w), 1292 (w), 1281 (w), 1252 (m), 1238 (m), 1213 (m), 1180 (m), 1163 (m), 1090 (w), 1067 (m), 1024 (m), 1001 (w), 968 (w), 951 (w), 868 (m), 845 (w), 808 (m), 741 (s), 702 (s), 689 (s), 665 (w), 644 (m), 619 (m). Anal. calcd. for $C_{14}H_9NS_3$: C 58.51., H 3.16, N 4.87, S 33.46; Found: C 58.44, H 2.99, N 4.80, S 33.07.

3 ^{1}H and ^{13}C NMR spectra

3.1 Bis(4-bromothiophen-3-yl)sulfane (3a)

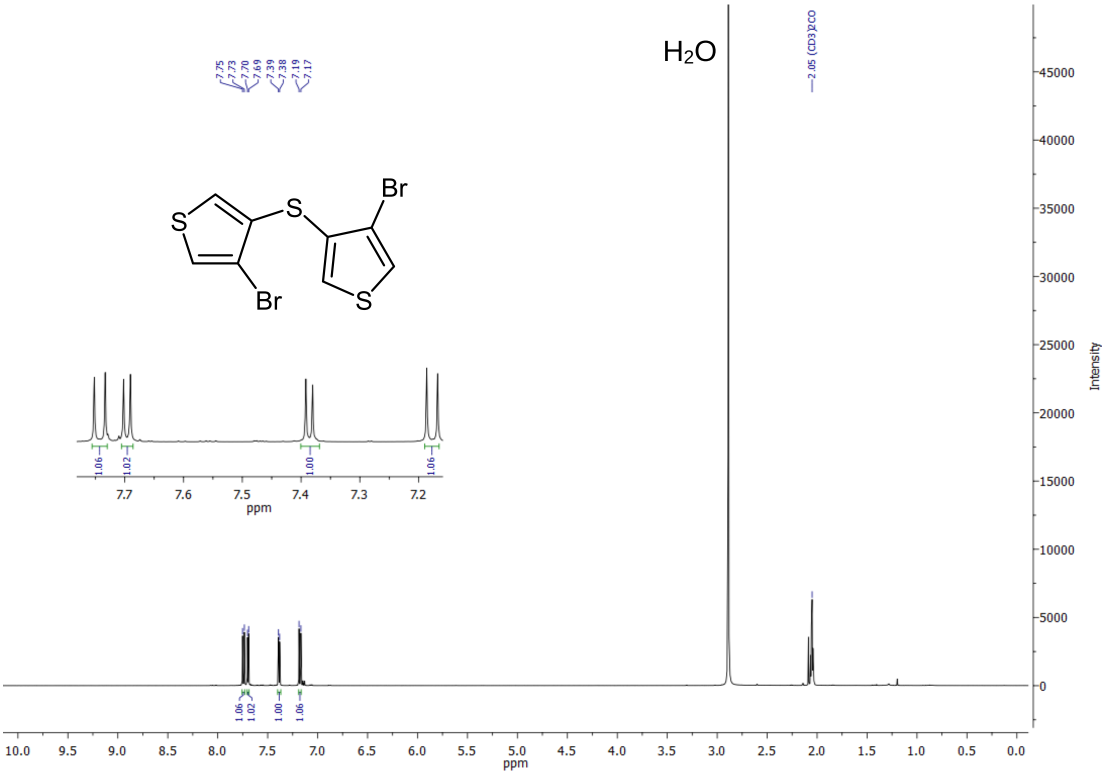

Figure 1. ^{1}H NMR spectrum of **3a** (acetone-d_6, 293K, 300MHz).

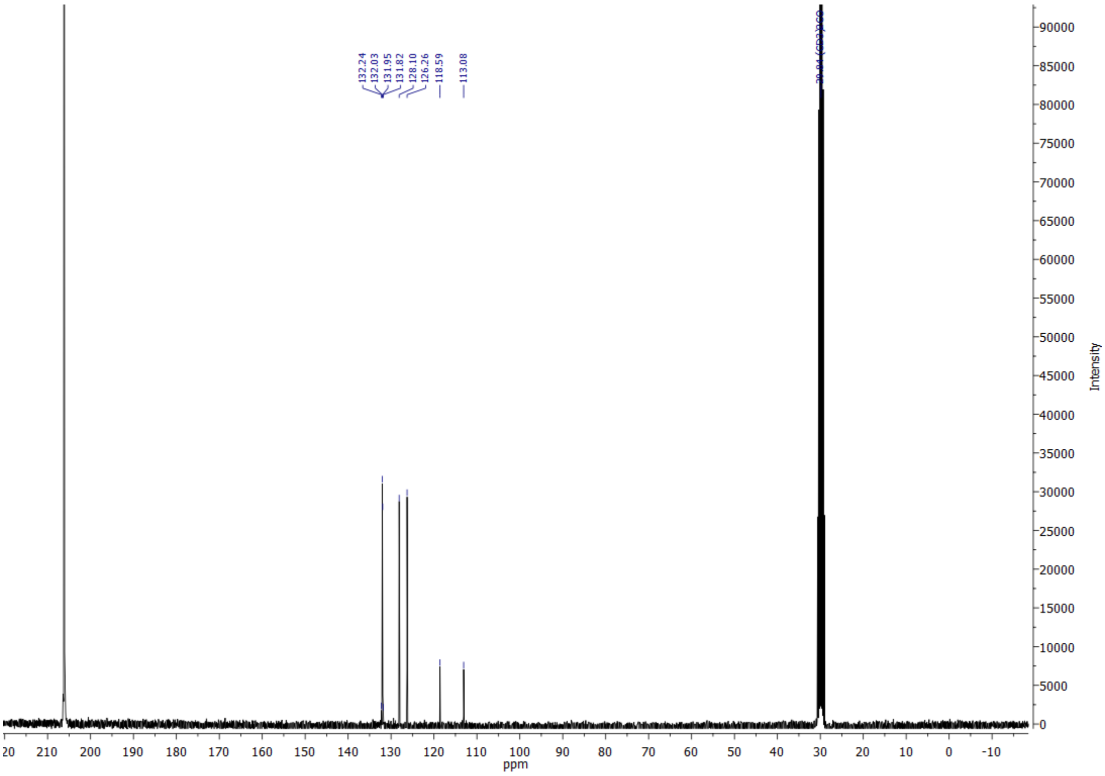

Figure 2. ^{13}C NMR spectrum of **3a** (acetone-d_6, 293K, 75MHz).

3.2 Di(thiophen-3-yl)sulfane (3b)

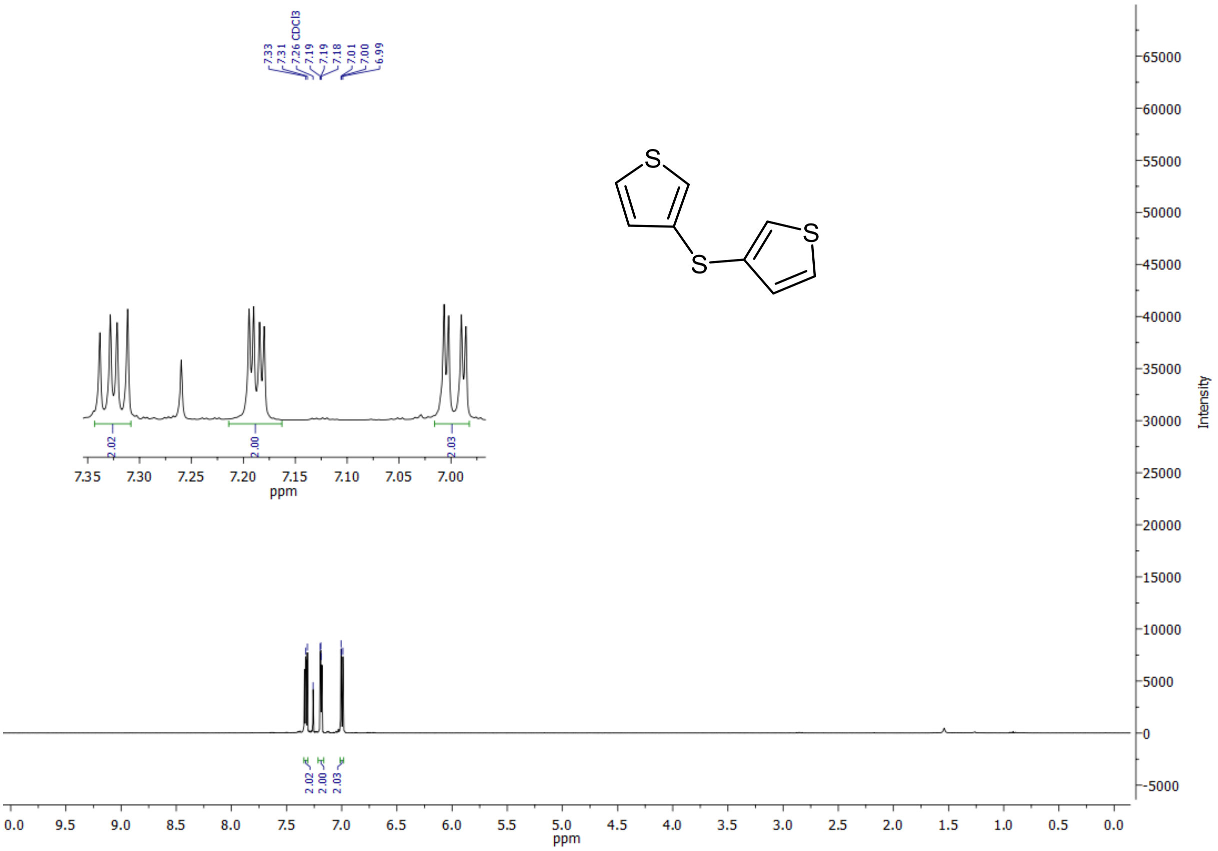

Figure 3. ^{1}H NMR spectrum of **3b** ($CDCl_3$, 293K, 300MHz).

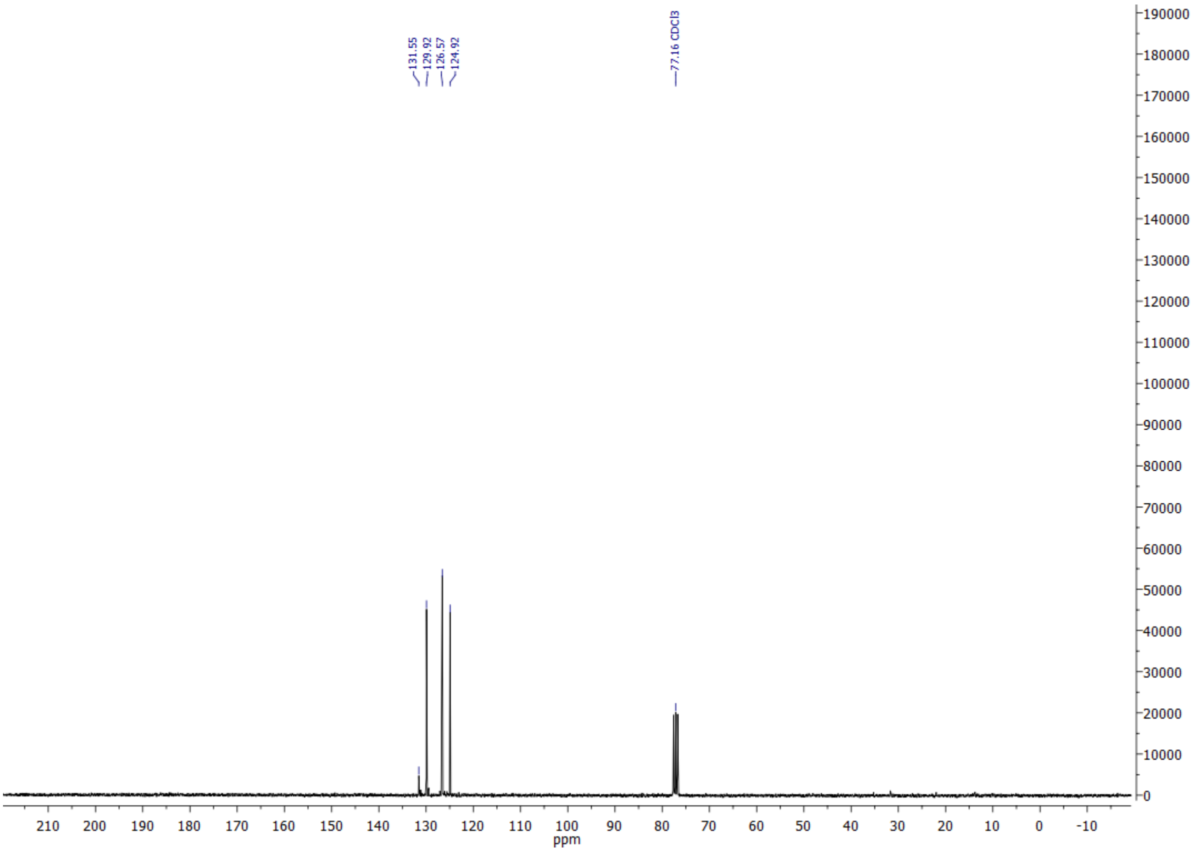

Figure 4. ^{13}C NMR spectrum of **3b** ($CDCl_3$, 293K, 75MHz).

3.3 Bis(4-bromothiophen-3-yl)sulfane (3c)

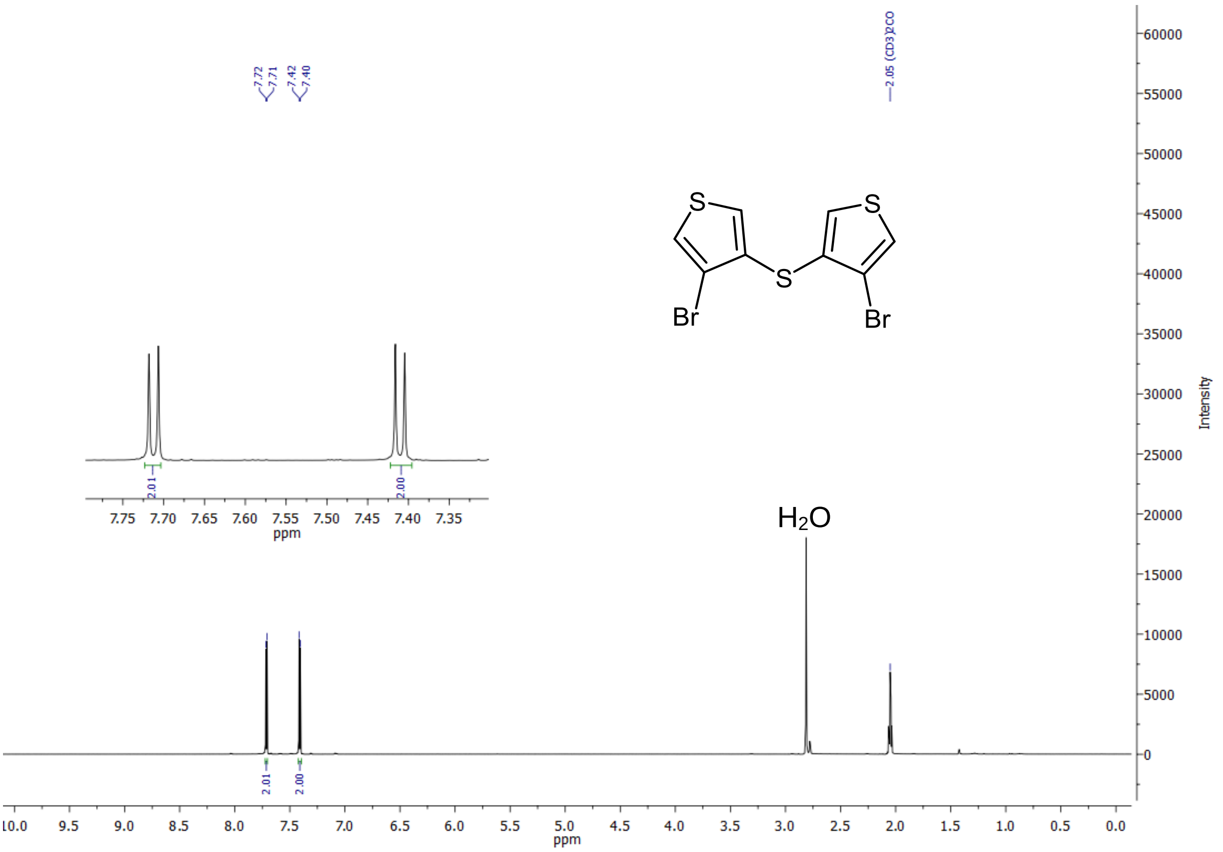

Figure 5. ^{1}H NMR spectrum of **3c** (acetone-d_6, 293K, 300MHz).

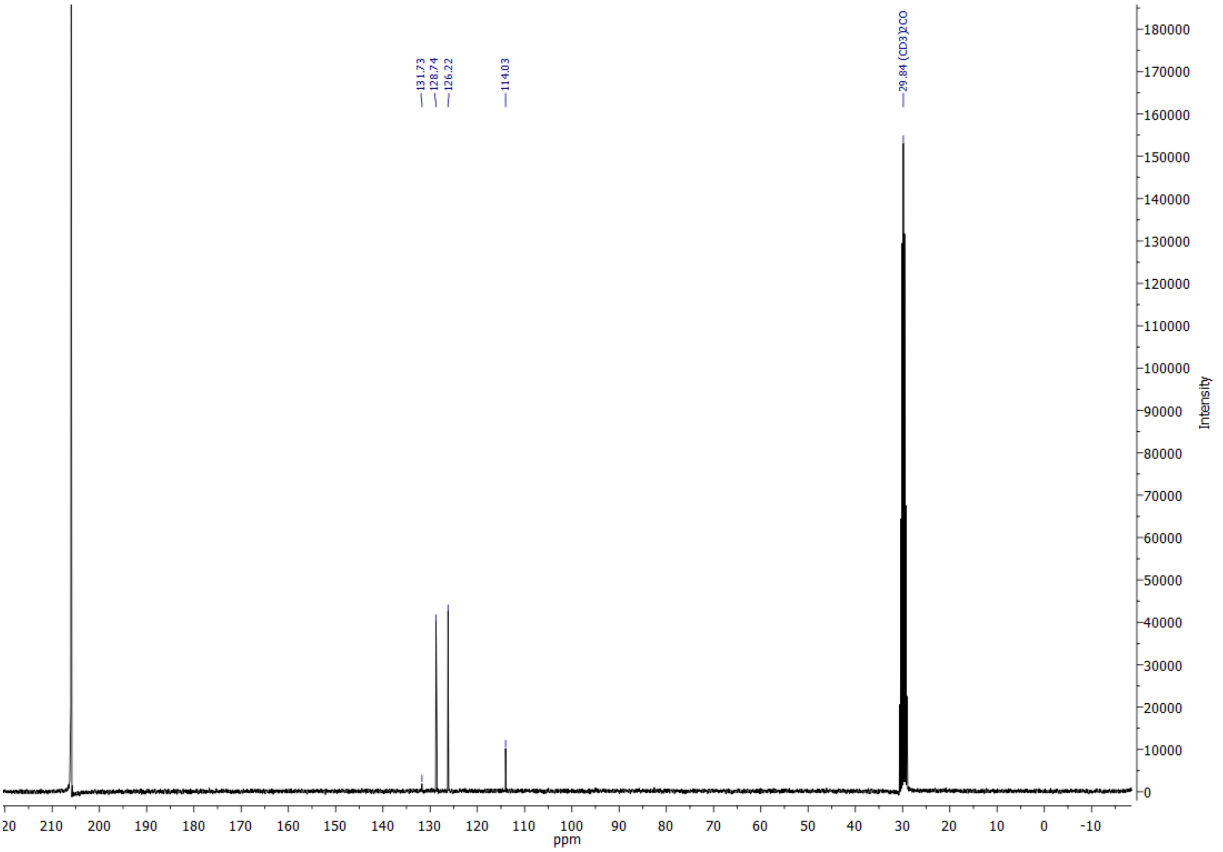

Figure 6. ^{13}C NMR spectrum of **3c** (acetone-d_6, 293K, 75MHz).

3.4 Bis(3-bromothiophen-2-yl)sulfane (3d)

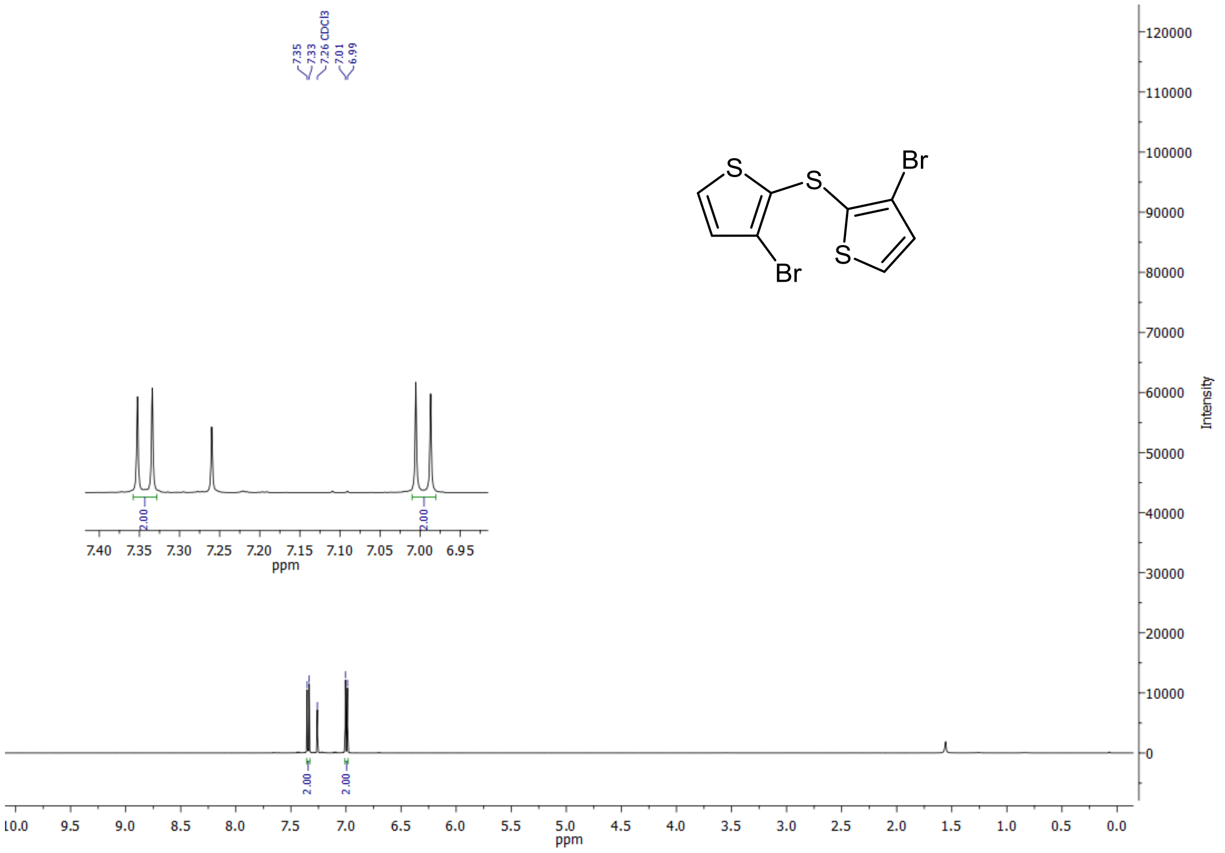

Figure 7. ^{1}H NMR spectrum of **3d** ($CDCl_3$, 293K, 300MHz).

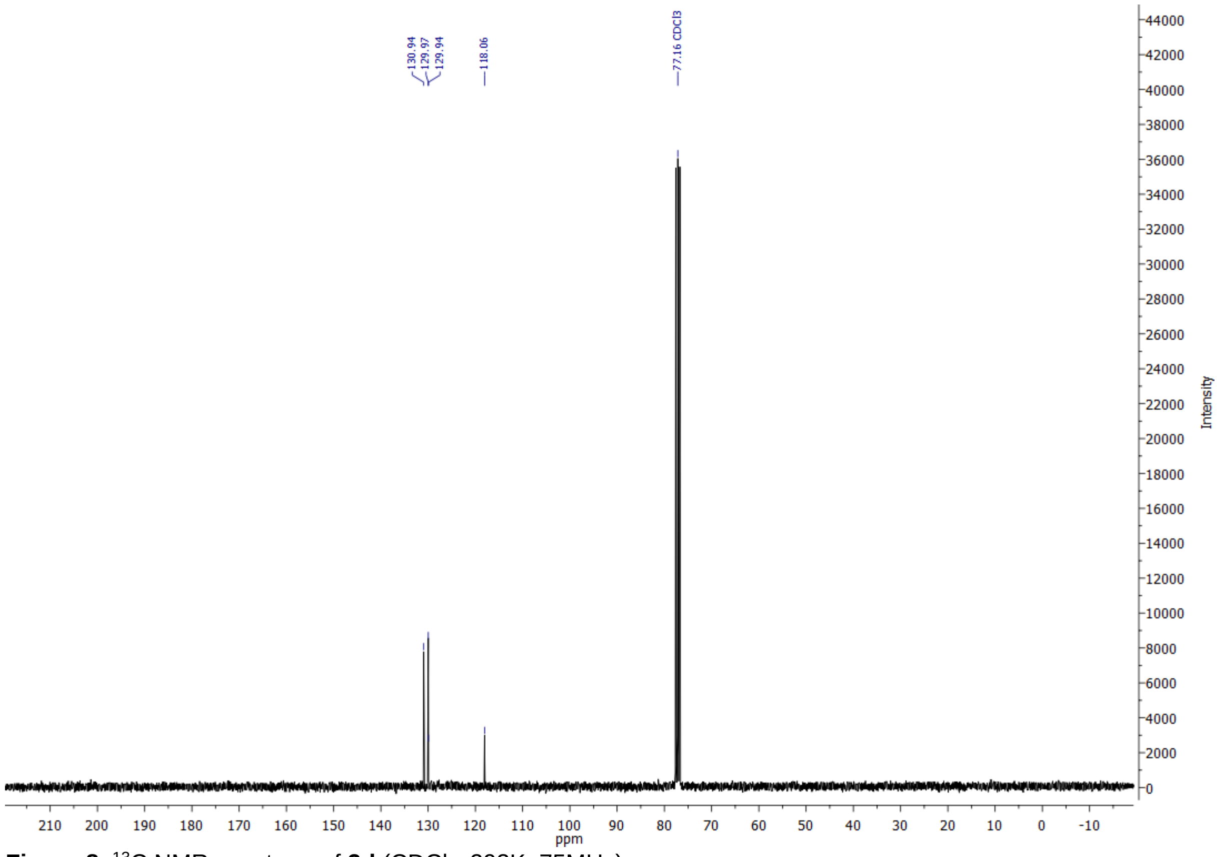

Figure 8. ^{13}C NMR spectrum of **3d** ($CDCl_3$, 293K, 75MHz).

3.5 Bis(2-iodothiophen-3-yl)sulfane (3e)

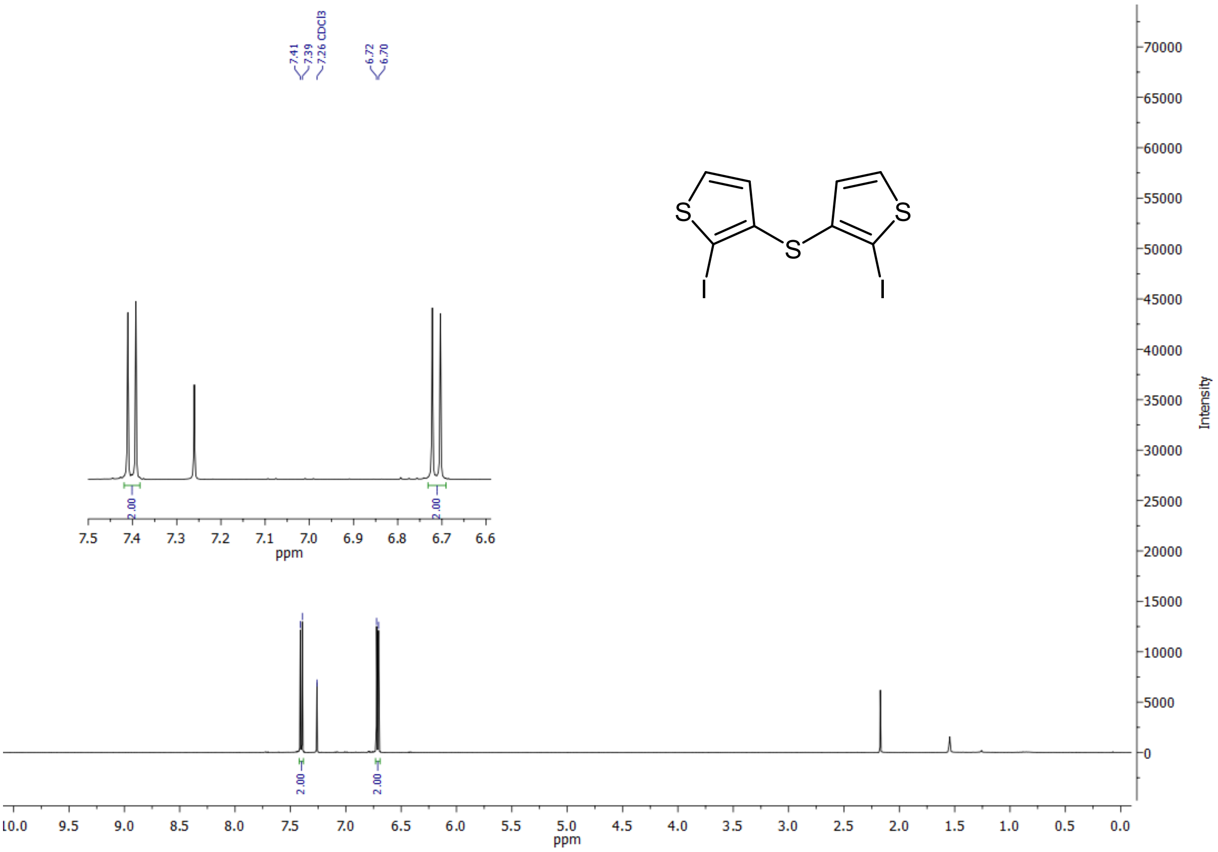

Figure 9. ^{1}H NMR spectrum of **3e** ($CDCl_3$, 293K, 300MHz).

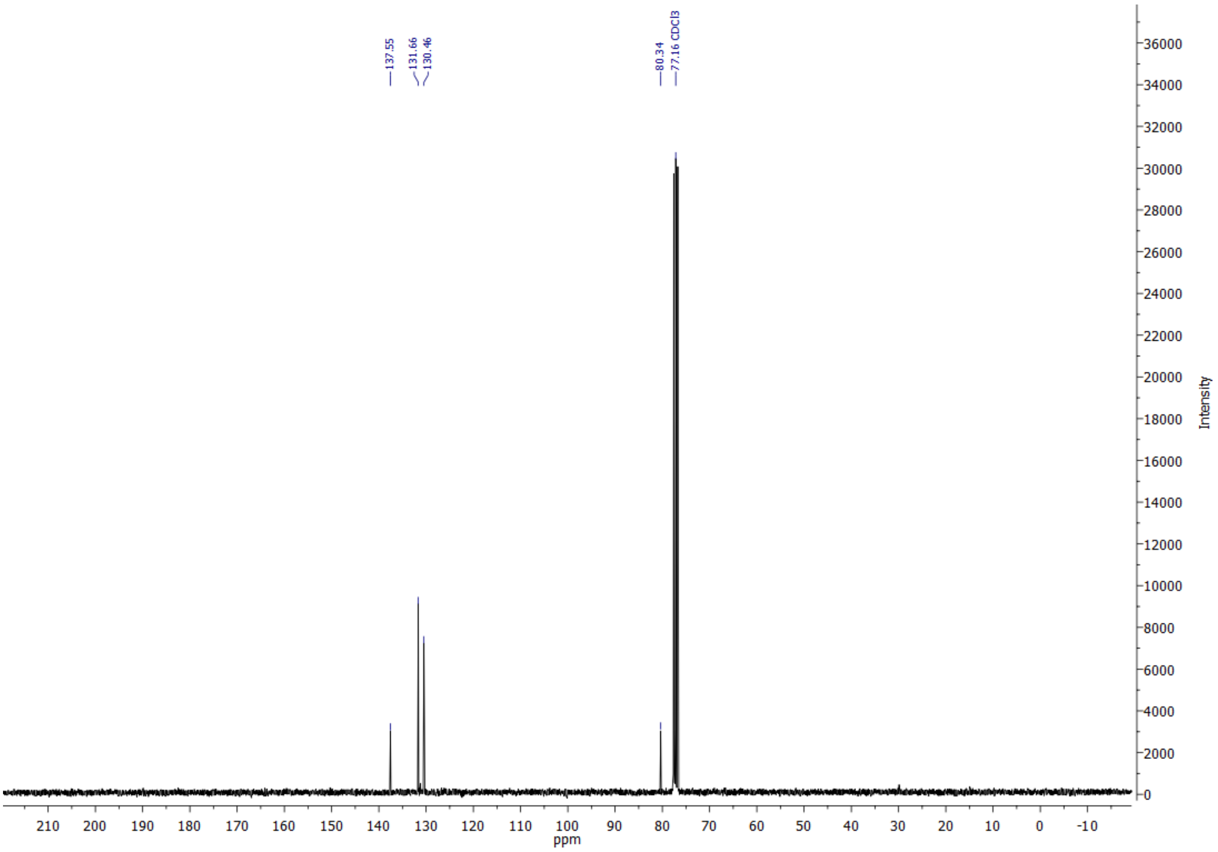

Figure 10. ^{13}C NMR spectrum of **3e** ($CDCl_3$, 293K, 75MHz).

3.6 4-Phenyl-4*H*-dithieno[2,3-b:3',4'-e][1,4]thiazine (2b)

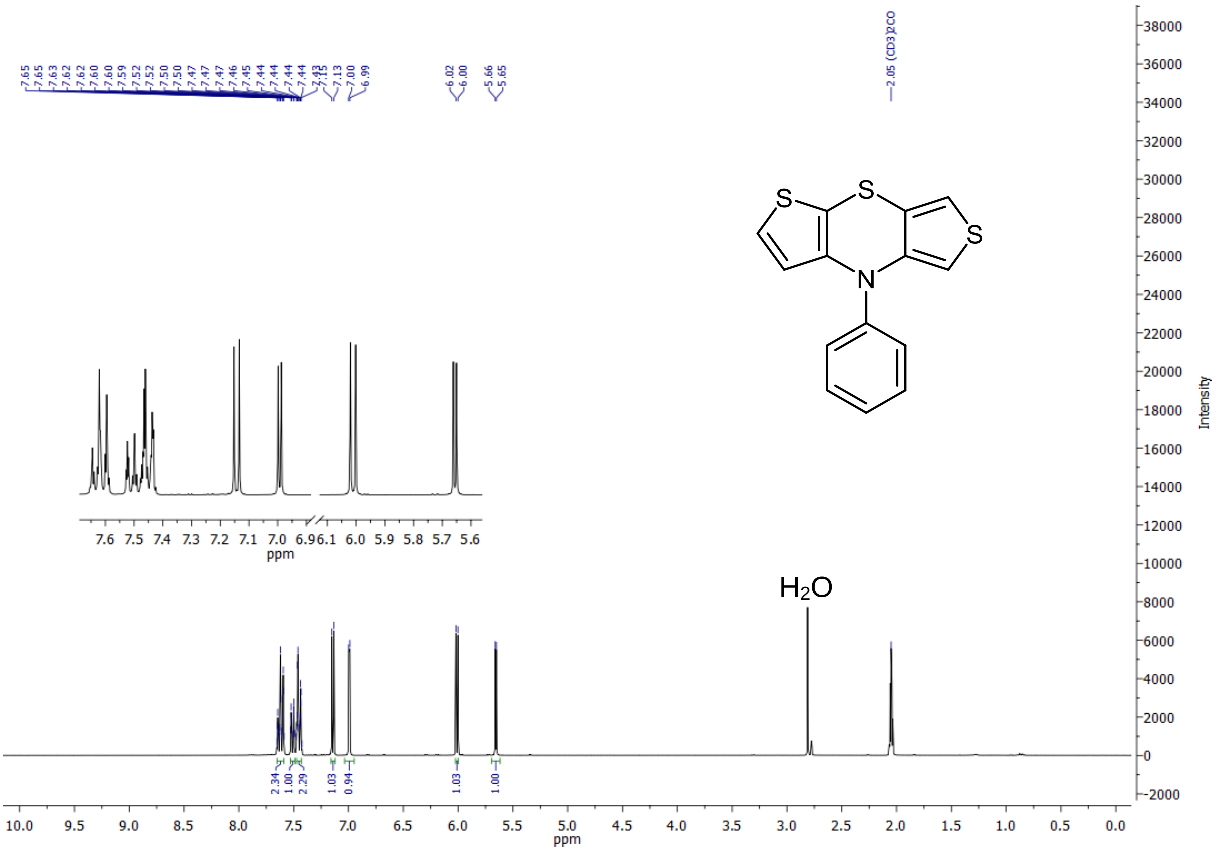

Figure 11. ^{1}H NMR spectrum of **2b** (acetone-d$_6$, 293K, 300MHz).

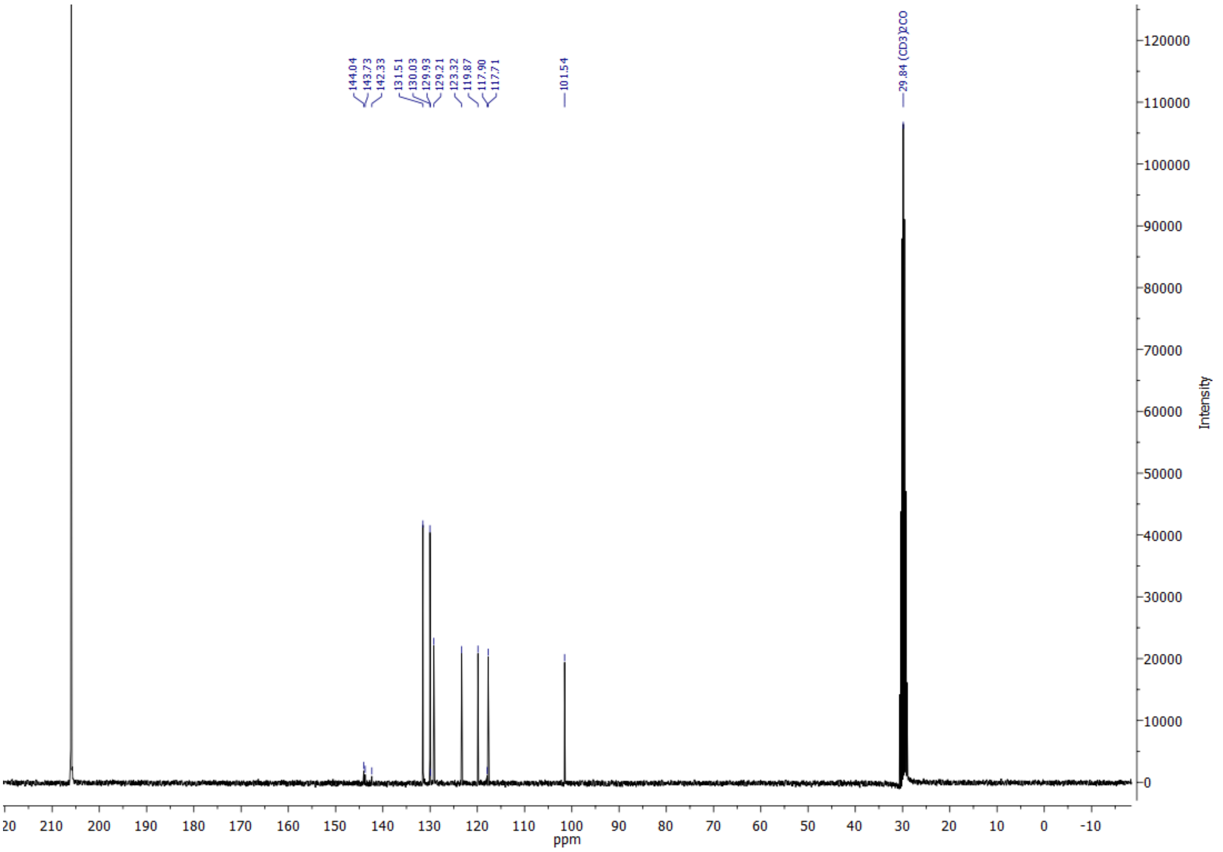

Figure 12. ^{13}C NMR spectrum of **2b** (acetone-d$_6$, 293K, 75MHz).

3.7 8-Phenyl-8*H*-dithieno[3,4-b:3',4'-e][1,4]thiazine (2c)

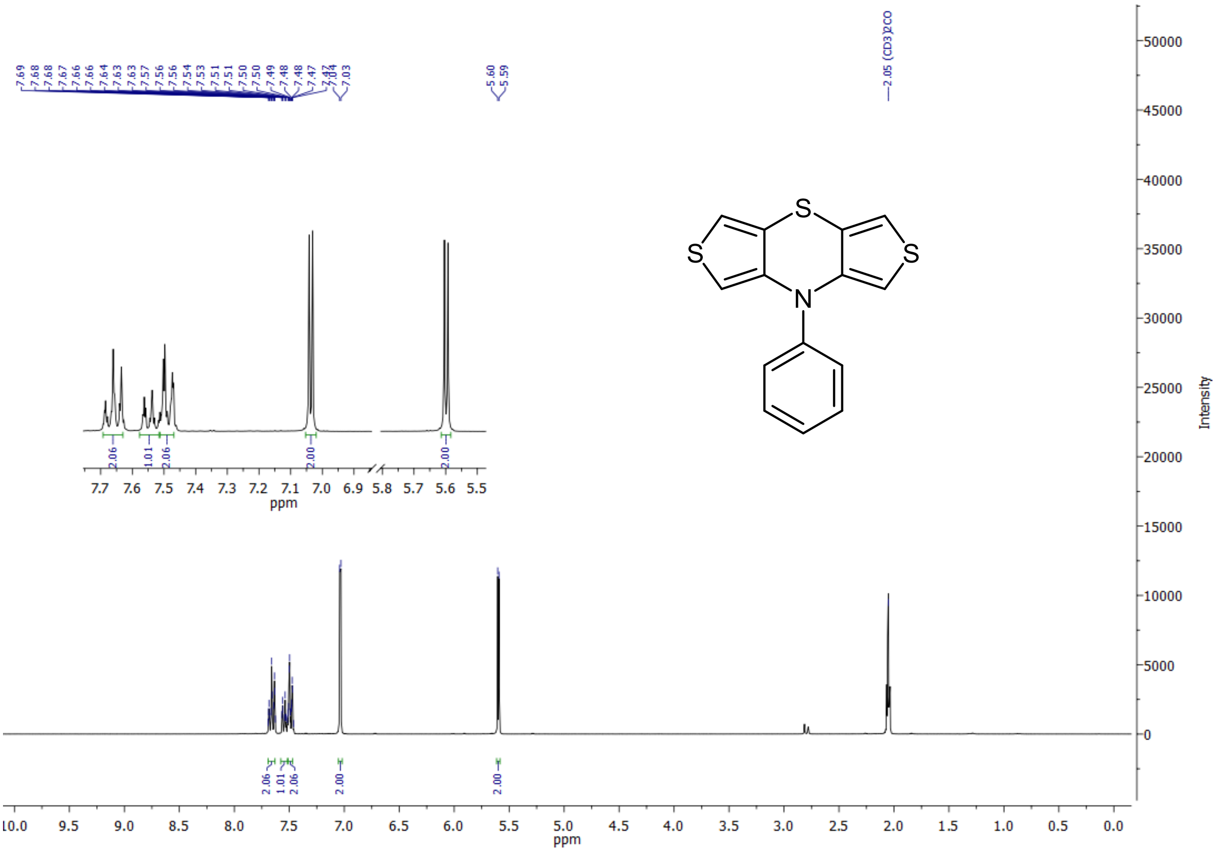

Figure 13. ^{1}H NMR spectrum of **2c** (acetone-d$_6$, 293K, 300MHz).

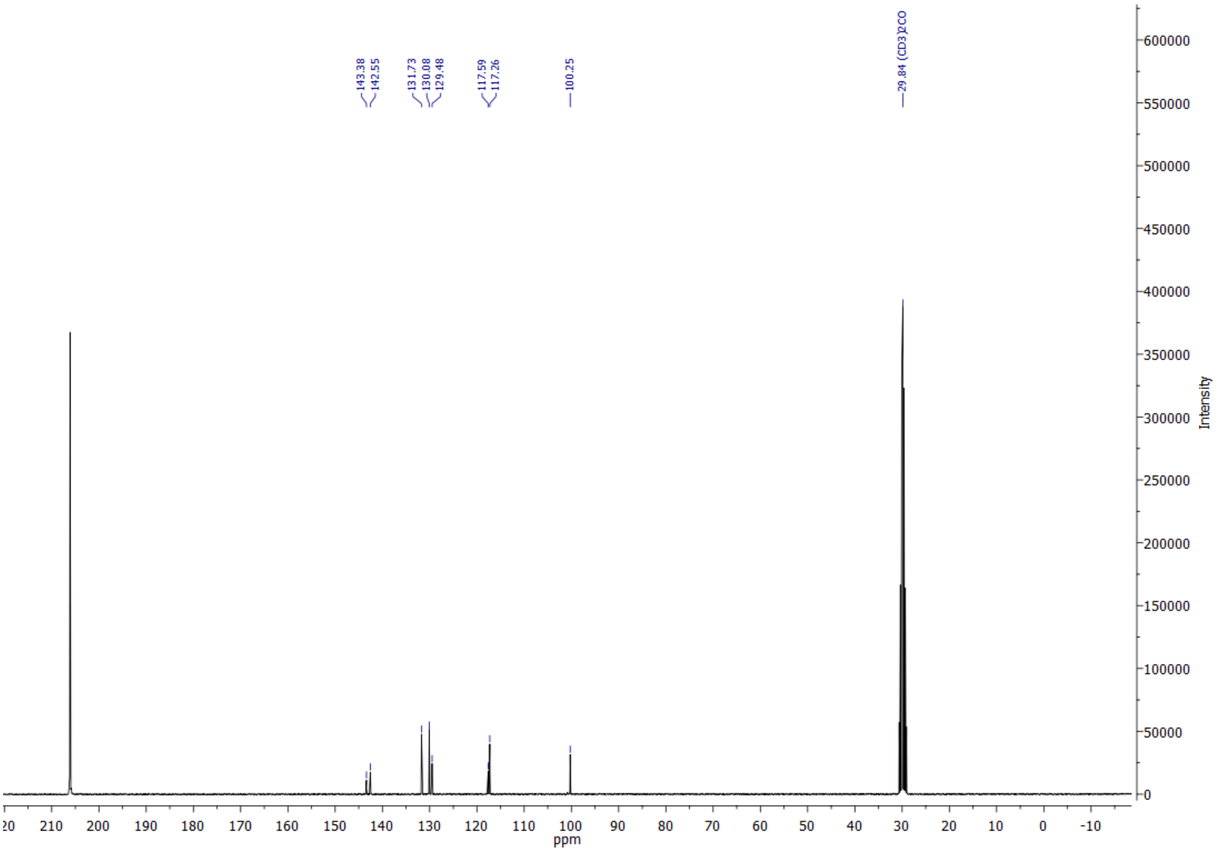

Figure 14. ^{13}C NMR spectrum of **2c** (acetone-d$_6$, 293K, 75MHz).

3.8 8-Phenyl-8*H*-dithieno[3,2-b:2',3'-e][1,4]thiazine (2d)

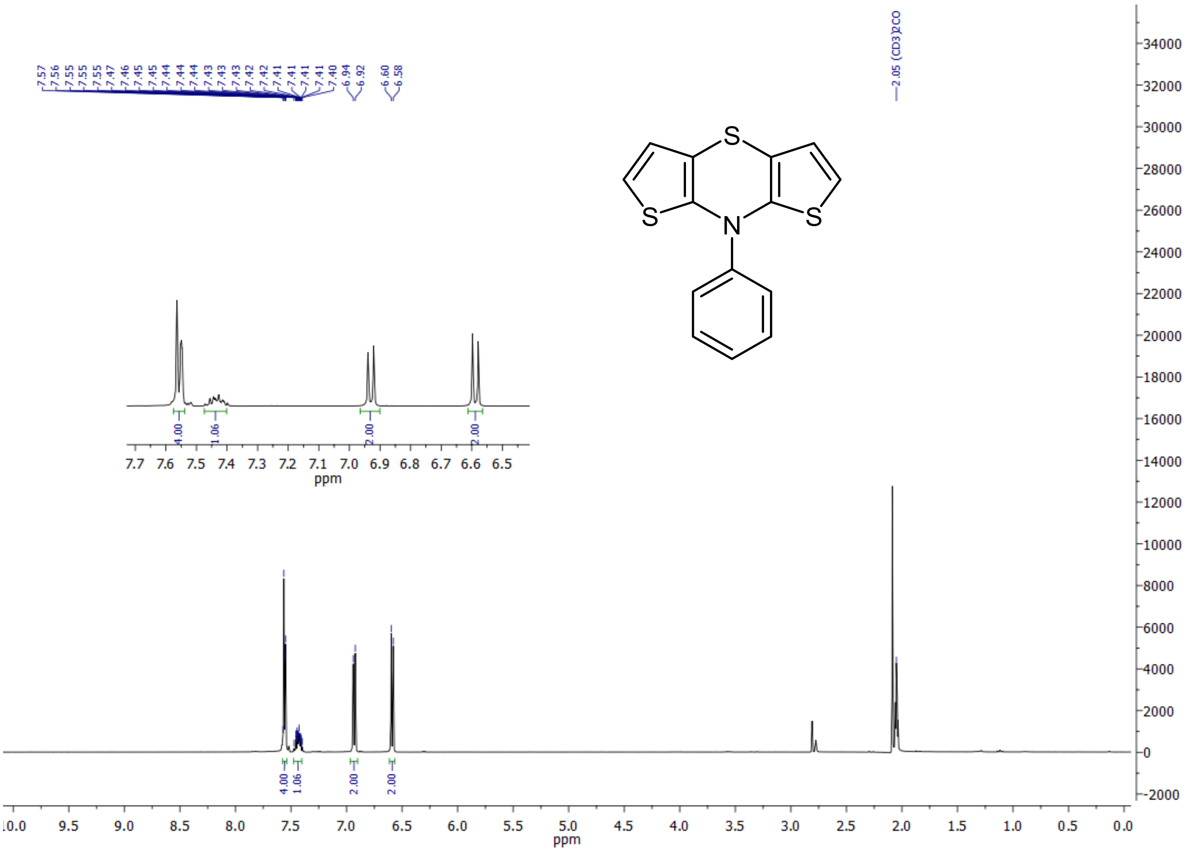

Figure 15. ^{1}H NMR spectrum of **2d** (aceton-d_6, 293K, 300MHz).

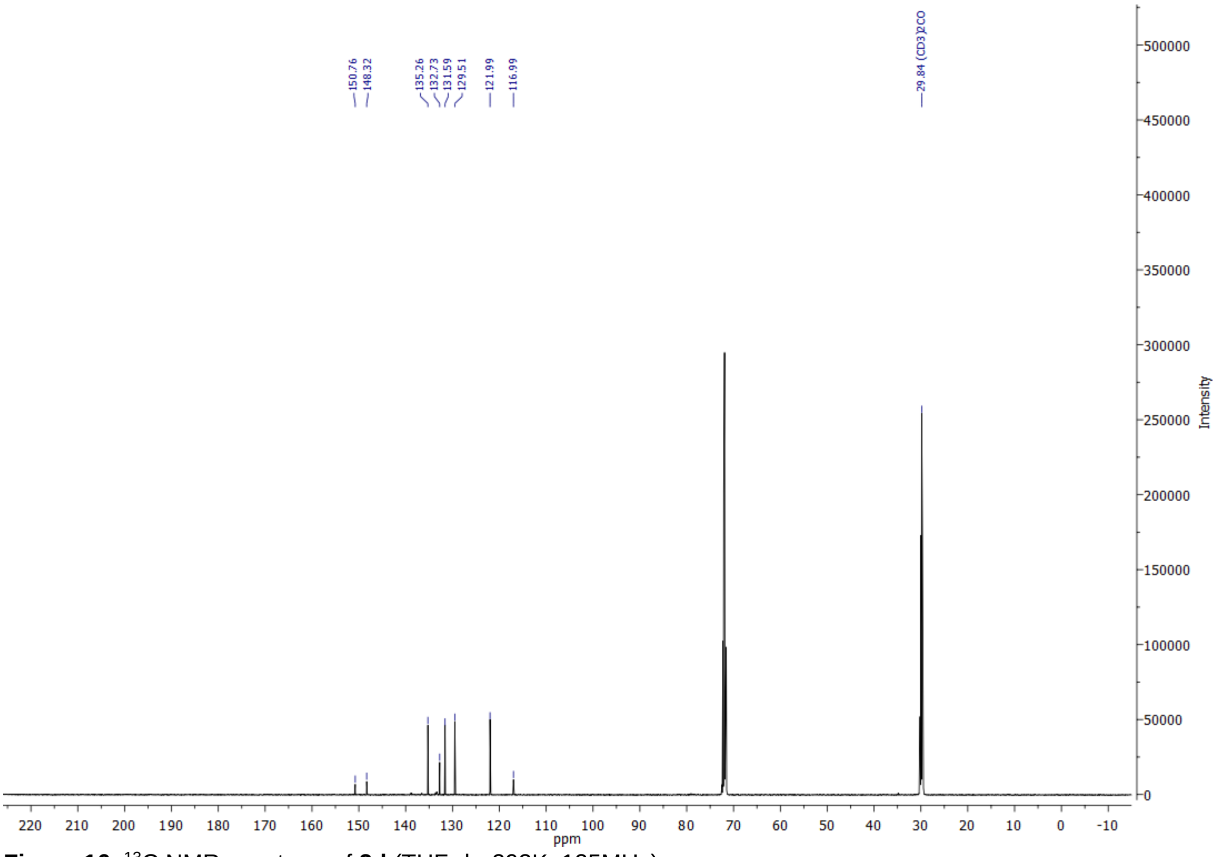

Figure 16. ^{13}C NMR spectrum of **2d** (THF-d_8, 293K, 125MHz).

4 Cyclovoltammetric Data

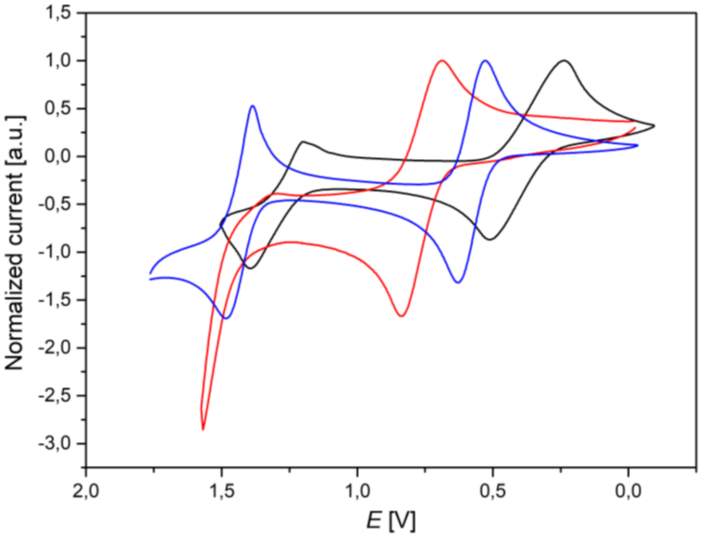

Figure 17. Cyclic voltammograms of the dithienothiazines **2b** (blue), **2c** (red) and **2d** (black), (0.1 M $[Bu_4N][PF_6]$, v = 100 mV/s, Pt-working, Ag/AgCl-reference and Pt-counter electrode, $[Me_{10}Fc]/[Me_{10}Fc]^+$ as an internal standard; $Me_{10}Fc$ = decamethylferrocene).

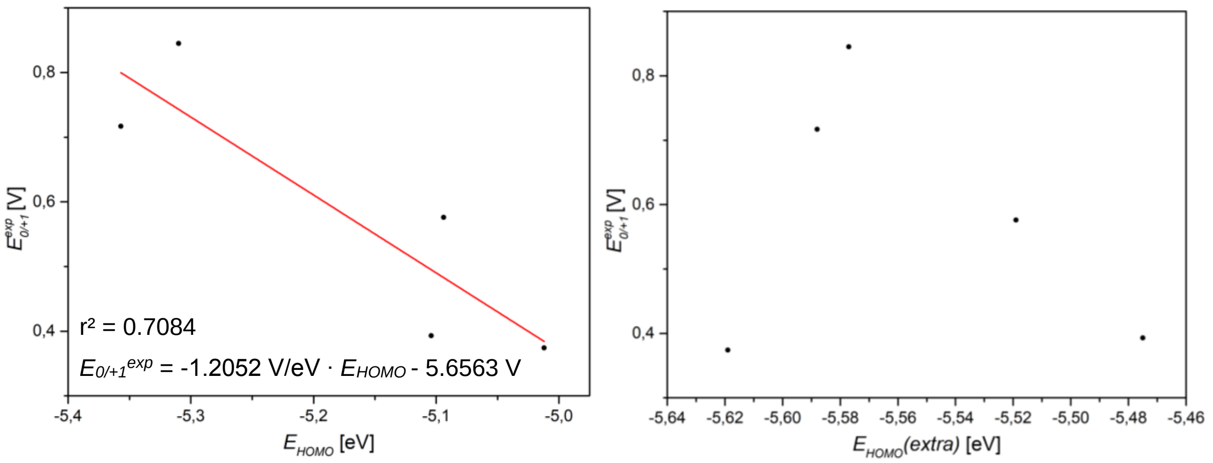

Figure 18. Measured first oxidation potential $E_{0/+1}^{exp}$ (0.1 M $[Bu_4N][PF_6]$, v = 100 mV/s, Pt-working, Ag/AgCl-reference and Pt-counter electrode, $[Me_{10}Fc]/[Me_{10}Fc]^+$ as an internal standard) vs. HOMO-energies E_{HOMO} (B3LYP/6-311G*, PCM CH_2Cl_2) of the compounds **1** and **2** in their intra (left) and extra conformation (right).

5 Absorption and Emission Spectra of Compounds 2

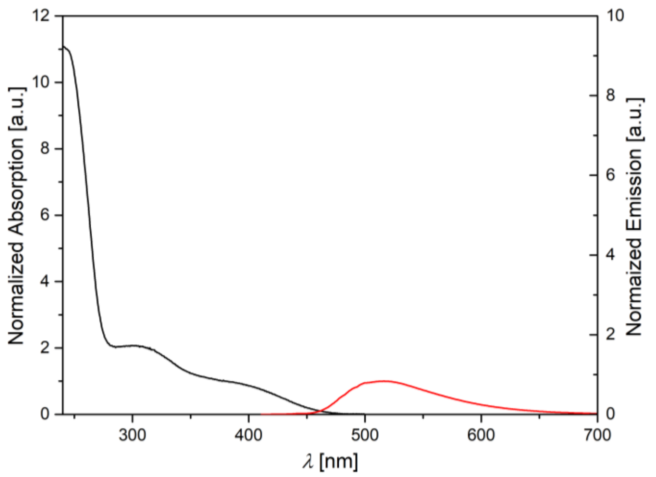

Figure 19. Absorption (black, CH_2Cl_2, T = 298 K, c(**2d**) = 10^{-5} M)) an emission spectrum (red, CH_2Cl_2, T = 298 K, c(**2d**) = 10^{-6} M) of **2d**.

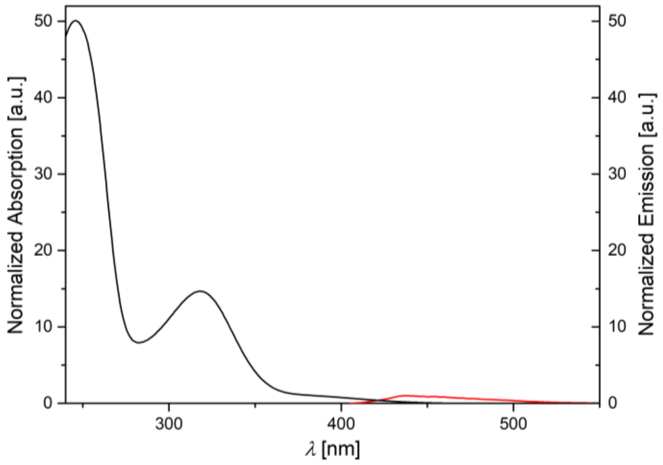

Figure 20. Absorption (black, CH_2Cl_2, T = 298 K, c(**2a**) = 10^{-5} M)) an emission spectrum (red, CH_2Cl_2, T = 298 K, c(**2a**) = 10^{-6} M) of **2a**.

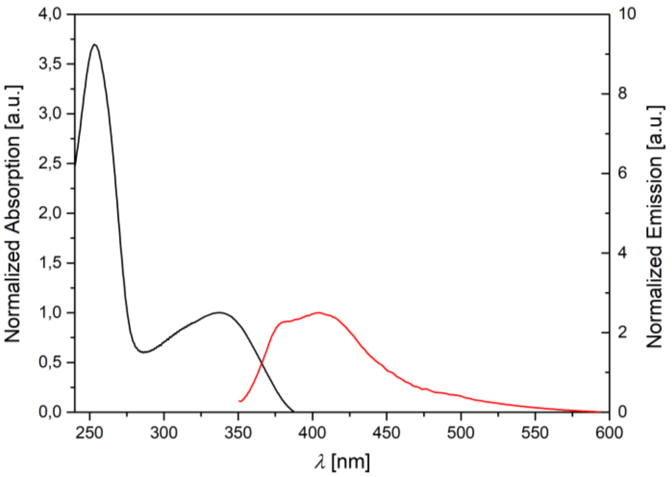

Figure 21. Absorption (black, CH_2Cl_2, T = 298 K, c(**2b**) = 10^{-5} M)) an emission spectrum (red, CH_2Cl_2, T = 298 K, c(**2b**) = 10^{-6} M) of **2b**.

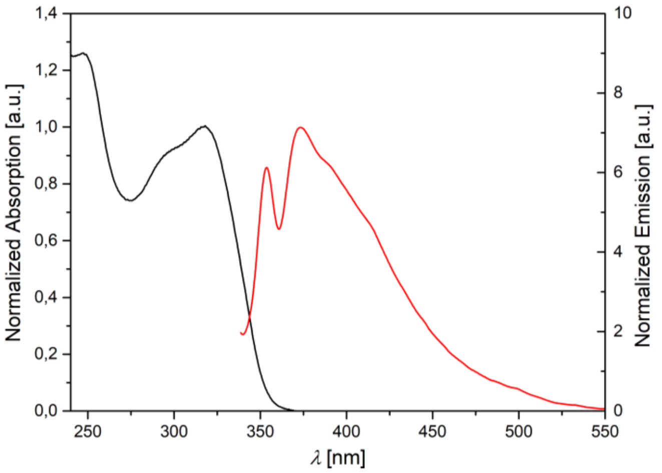

Figure 22. Absorption (black, CH_2Cl_2, T = 298 K, c(**2c**) = 10^{-5} M)) an emission spectrum (red, CH_2Cl_2, T = 298 K, c(**2c**) = 10^{-6} M) of **2c**.

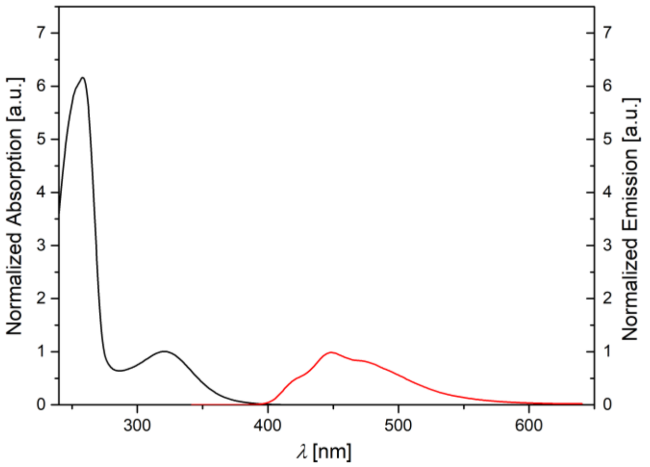

Figure 23. Absorption (black, CH_2Cl_2, T = 298 K, c(**1**) = 10^{-5} M)) an emission spectrum (red, CH_2Cl_2, T = 298 K, c(**1**) = 10^{-6} M) of **1**.

6 Data of Quantum Chemical Calculations

6.1 Computed xyz-coordinates, excitations of compounds 1 and 2 and selected properties derived from the DFT calculations

The ground state geometries of both the intra and the extra conformation of compounds **1** and **2** were optimized using the Gaussian09 program package,[5] the B3LYP functional[6] and the 6-311G* basis set.[7] The ground state geometries of the radical cations were optimized using the Gaussian09 program package,[5] the uB3LYP functional[6] and the 6-311G* basis set.[7] Excitation energies and the excited state geometry (S_1) of **2d** were calculated with TDDFT[8] methods implemented in the Gaussian09 program package using the same functional and basis set, that were used for the ground state optimizations. All optimized geometries were confirmed as minima by analytical frequency analyses. The polarizable continuum model (PCM) with dichloromethane as a solvent was applied for the calculations each.[9]

For the calculation of redox potentials (see chapter 6.2) the optimized ground state geometries of the intra conformations and the radical cations were reoptimized using the Gaussian09 program package,[5] the uB3LYP functional [6] and the 6-311G* basis set.[7] The reoptimizations were performed in the gas phase and all optimized geometries were confirmed as minima by analytical frequency analyses again. The SMD solvation model with dichloromethane as a solvent was applied afterwards to determine the solvation enthalpies.[10]

Bond orders (Wiberg, Tables 3 and 4) and HOMO compositions (Mulliken, Figure 24) were extracted from the Gaussian09 calculation outputs by the help of the Multiwfn software.[11]

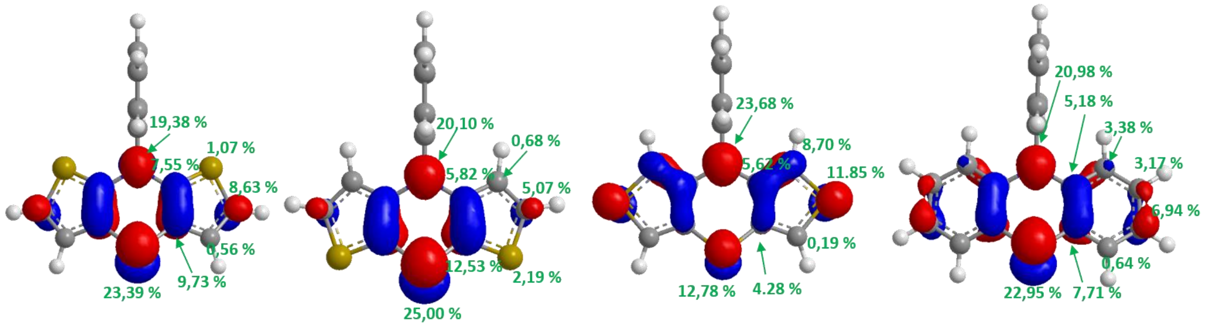

2d **2a** **2c** **1**

Figure 24. HOMO composition of dithienothiazines **2a**, **2c** and **2d** and phenothiazine **1** (B3LYP/6-311G*).[11]

DFT-calculated properties derived from the geometry optimizations of compounds **1** and **2** (*S*,*N*-folding angles ϑ, free enthalpies of switching from extra to intra conformation $\Delta G_{extra\rightarrow intra}$ and the HOMO- and LUMO-energies E_{HOMO} and E_{LUMO}) are listed in table 5.

Table 3. Change of the Wiberg bond-orders from neutral ground-state to the oxidized species (radical cation) $\Delta(D_0\text{-}S_0)$ in the dithienothiazine core of compounds **2a**, **2c** and **2d** (uB3LYP/6-311G*).[11]

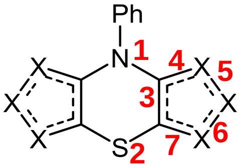

		1	2	3	4	5	6	7
**2d**	D_0	1.26	1.26	1.40	1.29	1.32	1.70	1.30
	S_0	1.17	1.16	1.56	1.27	1.31	1.73	1.28
	$\Delta(D_0\text{-}S_0)$ [%]	7.69	8.62	-10.26	1.57	0.76	-1.73	1.56
**2a**	D_0	1.22	1.29	1.40	1.25	1.72	1.33	1.31
	S_0	1.14	1.19	1.55	1.26	1.71	1.34	1.28
	$\Delta(D_0\text{-}S_0)$ [%]	7.02	8.40	-9.68	-0.79	0.58	-0.75	2.34
**2c**	D_0	1.24	1.25	1.16	1.48	1.43	1.34	1.64
	S_0	1.16	1.19	1.20	1.61	1.31	1.34	1.66
	$\Delta(D_0\text{-}S_0)$ [%]	6.90	5.04	-3.33	-8.07	9.16	0.00	-1.20

Table 4. Change of the Wiberg bond-orders from neutral ground-state to the oxidized species (radical cation) $\Delta(D_0\text{-}S_0)$ in the phenothiazine core of compound **1** (uB3LYP/6-311G*).[11]

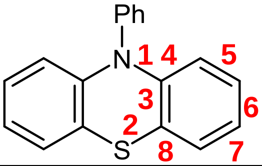

		1	2	3	4	5	6	7	8
**1**	D_0	1.22	1.27	1.28	1.33	1.56	1.40	1.55	1.40
	S_0	1.15	1.18	1.35	1.41	1.49	1.49	1.48	1.47
	$\Delta(D_0\text{-}S_0)$ [%]	6.09	7.63	-5.19	-5.67	4.70	-6.04	4.73	-4.76

Table 5. DFT-calculated properties derived from the geometry optimizations of compounds **1** and **2**: *S,N*-folding angles of the neutral intra-conformation $\vartheta_{intra}(S_0)$ and radical cations ϑ_{ox}, free enthalpies of switching from extra to intra conformation $\Delta G_{extra\rightarrow intra}$ and the HOMO- and LUMO-energies of the intra conformations $E_{HOMO,intra}$ and $E_{LUMO,intra}$.

	$E_{HOMO,intra}$ [a] [eV]	$E_{LUMO,intra}$ [a] [eV]	$\vartheta_{intra}(S_0)$ [a] [°]	ϑ_{ox} [b] [°]	$\Delta G_{extra\rightarrow intra}$ [a] [kcal/mol]
**2d**	-5.012	-0.935	144	180	-1.57
**2a**	-5.104	-1.020	144	180	-1.01
**2b**	-5.094	-0.878	155	180	-2.86
**2c**	-5.310	-0.760	158	180	-4.14
**1**	-5.357	-0.949	148	180	-1.89

a: B3LYP/6-311G*, PCM CH_2Cl_2; b: uB3LYP/6-311G*, PCM CH_2Cl_2

6.1.1.1 Computed xyz coordinates of compound 2a (B3LYP/6-311G* PCM CH_2Cl_2)

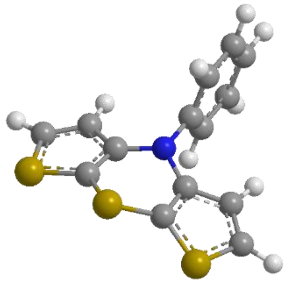

Figure 29. Optimized ground state geometry of **2a** (B3LYP/6-311G* PCM CH_2Cl_2).

C	-1.63447	1.30254	0.19100
C	-0.27447	1.20694	0.01879
N	0.42941	-0.00017	0.22396
C	-0.27534	-1.20704	0.01898
C	-1.63539	-1.30156	0.19115
S	-2.64737	0.00089	0.85197
C	0.29650	-2.43894	-0.43755
C	-0.63325	-3.42170	-0.60521
S	-2.24027	-2.87481	-0.24590
S	-2.23822	2.87595	-0.24677
C	-0.63098	3.42157	-0.60635
C	0.29816	2.43827	-0.43831
C	1.86667	-0.00054	0.17205
C	2.54046	-0.00238	-1.05223
C	3.93389	-0.00260	-1.07881
C	4.65636	-0.00094	0.11436
C	3.98347	0.00095	1.33532
C	2.58959	0.00115	1.36512
H	1.35190	-2.58106	-0.62534
H	-0.47552	-4.44065	-0.92567
H	-0.47253	4.44033	-0.92710
H	1.35365	2.57964	-0.62615
H	1.97260	-0.00354	-1.97651

H	4.45408	-0.00404	-2.03081
H	5.74101	-0.00111	0.09182
H	4.54189	0.00226	2.26536
H	2.05366	0.00256	2.30786

SCF Done: E(RB3LYP) = -1788.38174364 A.U. after 1 cycles

Zero-point correction= 0.190464 (Hartree/Particle)

Thermal correction to Energy= 0.205201

Thermal correction to Enthalpy= 0.206145

Thermal correction to Gibbs Free Energy= 0.146791

Sum of electronic and zero-point Energies= -1788.191280

Sum of electronic and thermal Energies= -1788.176543

Sum of electronic and thermal Enthalpies= -1788.175599

Sum of electronic and thermal Free Energies= -1788.234953

6.1.1.2 Computed excitations of compound 2a (B3LYP/6-311G* PCM CH_2Cl_2)

Excited State 1: Singlet-A 3.1379 eV 395.11 nm f=0.0044 <S**2>=0.000
74 -> 75 0.69268
This state for optimization and/or second-order correction.
Total Energy, E(TD-HF/TD-KS) = -1788.26642628
Copying the excited state density for this state as the 1-particle RhoCI density.

Excited State 2: Singlet-A 3.5682 eV 347.47 nm f=0.0001 <S**2>=0.000
74 -> 76 0.69625

Excited State 3: Singlet-A 3.8072 eV 325.66 nm f=0.0009 <S**2>=0.000
74 -> 75 0.11397
74 -> 78 0.67015
74 -> 79 -0.14571

Excited State 4: Singlet-A 3.8300 eV 323.72 nm f=0.0771 <S**2>=0.000
72 -> 75 -0.10163
74 -> 77 0.69486

Excited State 5: Singlet-A 4.1314 eV 300.10 nm f=0.0001 <S**2>=0.000

74 -> 78 0.16351
74 -> 79 0.67781

Excited State 6: Singlet-A 4.2713 eV 290.27 nm f=0.0021 <S**2>=0.000
74 -> 80 0.70165

Excited State 7: Singlet-A 4.7280 eV 262.24 nm f=0.0101 <S**2>=0.000
73 -> 75 -0.29436
74 -> 81 0.61951

Excited State 8: Singlet-A 4.8178 eV 257.35 nm f=0.0009 <S**2>=0.000
74 -> 82 0.69322

Excited State 9: Singlet-A 4.8679 eV 254.70 nm f=0.1784 <S**2>=0.000
73 -> 75 0.62235
74 -> 81 0.28946

Excited State 10: Singlet-A 5.1738 eV 239.64 nm f=0.0104 <S**2>=0.000
73 -> 76 0.69497

6.1.1.3 Computed xyz coordinates of extra conformation of compound 2a (B3LYP/6-311G* PCM CH_2Cl_2)

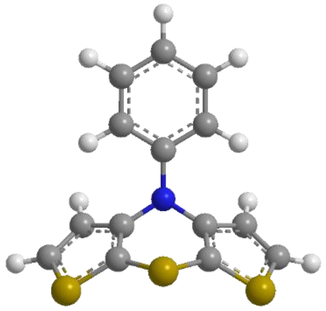

Figure 30. Optimized ground state geometry of extra conformation of **2a** (B3LYP/6-311G* PCM CH_2Cl_2).

C	1.38451	1.30534	-0.41027
C	0.28958	1.19299	0.40891
N	-0.48834	-0.00001	0.43083
C	0.28962	-1.19299	0.40891
C	1.38454	-1.30533	-0.41027

S	1.91495	0.00002	-1.48903
C	0.13539	-2.32338	1.27415
C	1.09480	-3.26926	1.07303
S	2.25632	-2.78562	-0.12848
S	2.25623	2.78568	-0.12849
C	1.09471	3.26929	1.07301
C	0.13533	2.32338	1.27415
C	-1.86249	-0.00002	0.09101
C	-2.56452	1.20530	-0.08597
C	-3.92151	1.19731	-0.39530
C	-4.61775	-0.00004	-0.54642
C	-3.92147	-1.19740	-0.39535
C	-2.56449	-1.20537	-0.08604
H	-0.65751	-2.41855	2.00432
H	1.20094	-4.22992	1.55473
H	1.20081	4.22996	1.55470
H	-0.65753	2.41851	2.00437
H	-2.05200	2.15404	0.00006
H	-4.43334	2.14559	-0.52643
H	-5.67535	-0.00007	-0.78633
H	-4.43330	-2.14567	-0.52653
H	-2.05193	-2.15408	-0.00006

SCF Done: E(RB3LYP) = -1788.38113365   A.U. after   1 cycles

Zero-point correction= 0.190644 (Hartree/Particle)

Thermal correction to Energy= 0.205153

Thermal correction to Enthalpy= 0.206098

Thermal correction to Gibbs Free Energy= 0.147799

Sum of electronic and zero-point Energies= -1788.190489

Sum of electronic and thermal Energies= -1788.175980

Sum of electronic and thermal Enthalpies= -1788.175036

Sum of electronic and thermal Free Energies= -1788.233335

6.1.1.4 Computed xyz coordinates of radical cation 2a^{+} of compound 2a (uB3LYP/6-311G* PCM CH_2Cl_2)

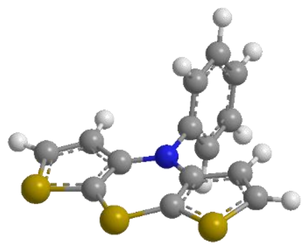

Figure 31: Optimized ground state geometry of radical cation of **2a** (uB3LYP/6-311G* PCM CH_2Cl_2).

C	1.66255	1.30872	0.00001
C	0.26820	1.20627	-0.00034
N	-0.40648	-0.00001	-0.00033
C	0.26821	-1.20627	-0.00035
C	1.66257	-1.30871	0.00002
S	2.79880	0.00002	0.00051
C	-0.36174	-2.48930	-0.00055
C	0.54084	-3.50421	-0.00030
S	2.19016	-2.96230	-0.00002
S	2.19012	2.96233	0.00017
C	0.54078	3.50421	-0.00046
C	-0.36178	2.48929	-0.00055
C	-1.85940	-0.00001	-0.00010
C	-2.53758	-0.00012	1.21597
C	-3.93096	-0.00012	1.20963
C	-4.62559	-0.00002	0.00055
C	-3.93149	0.00009	-1.20886
C	-2.53813	0.00009	-1.21586
H	-1.43141	-2.63733	-0.00084
H	0.34212	-4.56553	-0.00034
H	0.34205	4.56553	-0.00061

H	-1.43145	2.63731	-0.00087
H	-1.98345	-0.00019	2.14762
H	-4.47070	-0.00021	2.14967
H	-5.70984	-0.00002	0.00075
H	-4.47171	0.00017	-2.14863
H	-1.98443	0.00017	-2.14778

SCF Done: E(UB3LYP) = -1788.21037634 A.U. after 1 cycles

Zero-point correction= 0.191724 (Hartree/Particle)

Thermal correction to Energy= 0.206203

Thermal correction to Enthalpy= 0.207148

Thermal correction to Gibbs Free Energy= 0.148077

Sum of electronic and zero-point Energies= -1788.018652

Sum of electronic and thermal Energies= -1788.004173

Sum of electronic and thermal Enthalpies= -1788.003229

Sum of electronic and thermal Free Energies= -1788.062299

6.1.1.5 Reoptimization of compound 2a (uB3LYP/6-311G*)

Intra conformation of compound **2a** in the gas phase (uB3LYP/6-311G*):

SCF Done: E(UB3LYP) = -1788.37535559 A.U. after 1 cycles
Zero-point correction= 0.190566 (Hartree/Particle)
Thermal correction to Energy= 0.205271
Thermal correction to Enthalpy= 0.206215
Thermal correction to Gibbs Free Energy= 0.146948
Sum of electronic and zero-point Energies= -1788.184790
Sum of electronic and thermal Energies= -1788.170085
Sum of electronic and thermal Enthalpies= -1788.169141
Sum of electronic and thermal Free Energies= -1788.228407

Intra conformation of compound **2a** in CH_2Cl_2 (uB3LYP/6-311G* SMD CH_2Cl_2):

SCF Done: E(UB3LYP) = -1788.39740489 A.U. after 13 cycles

Extra conformation of compound **2a** in the gas phase (uB3LYP/6-311G*):

SCF Done: E(UB3LYP) = -1788.37493912   A.U. after   1 cycles

Zero-point correction= 0.190773 (Hartree/Particle)

Thermal correction to Energy= 0.205259

Thermal correction to Enthalpy= 0.206203

Thermal correction to Gibbs Free Energy= 0.148018

Sum of electronic and zero-point Energies= -1788.184166

Sum of electronic and thermal Energies= -1788.169680

Sum of electronic and thermal Enthalpies= -1788.168736

Sum of electronic and thermal Free Energies= -1788.226921

Extra conformation of compound **2a** in CH_2Cl_2 (uB3LYP/6-311G* SMD CH_2Cl_2):

SCF Done: E(UB3LYP) = -1788.39632282   A.U. after   13 cycles

Radical cation of compound **2a** in the gas phase (uB3LYP/6-311G*):

SCF Done: E(UB3LYP) = -1788.15393212   A.U. after   1 cycles

Zero-point correction= 0.191715 (Hartree/Particle)

Thermal correction to Energy= 0.206191

Thermal correction to Enthalpy= 0.207135

Thermal correction to Gibbs Free Energy= 0.148095

Sum of electronic and zero-point Energies= -1787.962217

Sum of electronic and thermal Energies= -1787.947742

Sum of electronic and thermal Enthalpies= -1787.946797

Sum of electronic and thermal Free Energies= -1788.00583

Radical cation of compound **2a** in CH_2Cl_2 (uB3LYP/6-311G* SMD CH_2Cl_2):

SCF Done: E(UB3LYP) = -1788.22887656   A.U. after   18 cycles

6.1.2.1 Computed xyz coordinates of compound 2b (B3LYP/6-311G* PCM CH_2Cl_2)

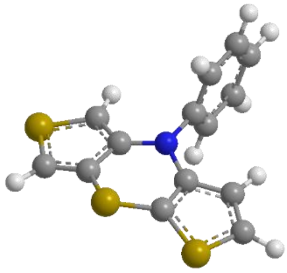

Figure 35. Optimized ground state geometry of **2b** (B3LYP/6-311G* PCM CH_2Cl_2).

C	-1.55959	1.52837	0.12714
C	-0.15402	1.21925	0.03834
N	0.34375	-0.08511	0.21155
C	-0.51435	-1.18520	0.01191
C	-1.88071	-1.10105	0.12569
S	-2.77204	0.33327	0.65490
C	-1.83709	2.84290	-0.09646
S	-0.39465	3.76023	-0.38181
C	0.59170	2.33585	-0.23336
C	-0.08483	-2.50570	-0.34561
C	-1.12132	-3.37742	-0.49277
S	-2.65949	-2.61694	-0.23484
C	1.76735	-0.28706	0.15581
C	2.43211	-0.32950	-1.07195
C	3.81136	-0.52552	-1.10706
C	4.52670	-0.67778	0.08081
C	3.86146	-0.63368	1.30526
C	2.48165	-0.43687	1.34415
H	-2.80066	3.32961	-0.07942
H	1.65984	2.40815	-0.35932
H	0.95101	-2.78617	-0.47795
H	-1.08053	-4.42592	-0.74659
H	1.86813	-0.21111	-1.99091
H	4.32610	-0.56092	-2.06135
H	5.60030	-0.83101	0.05143
H	4.41471	-0.75202	2.23080
H	1.95121	-0.39931	2.28924

SCF Done: E(RB3LYP) = -1788.38694516   A.U. after   1 cycles

Zero-point correction= 0.190446 (Hartree/Particle)

Thermal correction to Energy= 0.205151

Thermal correction to Enthalpy= 0.206096

Thermal correction to Gibbs Free Energy= 0.146731

Sum of electronic and zero-point Energies= -1788.196499

Sum of electronic and thermal Energies= -1788.181794

Sum of electronic and thermal Enthalpies= -1788.180850

Sum of electronic and thermal Free Energies= -1788.240214

6.1.2.2 Computed excitations of compound 2b (B3LYP/6-311G* PCM CH_2Cl_2)

Excited State 1: Singlet-A 3.5193 eV 352.30 nm f=0.0225 <S**2>=0.000
74 -> 75 0.64543
74 -> 76 0.26081
This state for optimization and/or second-order correction.
Total Energy, E(TD-HF/TD-KS) = -1788.25761242
Copying the excited state density for this state as the 1-particle RhoCI density.

Excited State 2: Singlet-A 3.6485 eV 339.82 nm f=0.0094 <S**2>=0.000
74 -> 75 -0.22516
74 -> 76 0.56576
74 -> 77 -0.33974

Excited State 3: Singlet-A 3.8036 eV 325.96 nm f=0.0893 <S**2>=0.000
74 -> 75 -0.11194
74 -> 76 0.31663
74 -> 77 0.61297

Excited State 4: Singlet-A 3.9500 eV 313.88 nm f=0.0304 <S**2>=0.000
74 -> 78 0.65400
74 -> 79 -0.21752

Excited State 5: Singlet-A 4.2506 eV 291.68 nm f=0.0211 <S**2>=0.000
74 -> 78 0.22920
74 -> 79 0.65209

Excited State 6: Singlet-A 4.5083 eV 275.01 nm f=0.0044 <S**2>=0.000
74 -> 80 0.69176

Excited State 7: Singlet-A 4.6127 eV 268.79 nm f=0.0167 <S**2>=0.000

73 -> 75 -0.26544
74 -> 81 0.62400

Excited State 8: Singlet-A 4.7044 eV 263.55 nm f=0.1263 <S**2>=0.000
73 -> 75 0.62680
73 -> 76 0.13530
74 -> 81 0.26327

Excited State 9: Singlet-A 4.8099 eV 257.77 nm f=0.0301 <S**2>=0.000
73 -> 75 -0.10351
73 -> 76 0.61610
73 -> 77 -0.28735

Excited State 10: Singlet-A 4.9775 eV 249.09 nm f=0.0332 <S**2>=0.000
73 -> 76 0.25422
73 -> 77 0.61329
73 -> 78 -0.21146

6.1.2.3 Computed xyz coordinates of extra conformation of compound 2b (B3LYP/6-311G* PCM CH_2Cl_2)

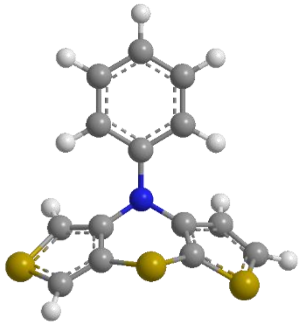

Figure 36. Optimized ground state geometry of extra conformation of **2b** (B3LYP/6-311G* PCM CH_2Cl_2).

C	-1.41050	1.35955	0.53658
C	-0.27218	1.21109	-0.33050
N	0.42983	-0.02382	-0.40908
C	-0.42098	-1.16484	-0.43803
C	-1.50473	-1.26053	0.39771
S	-1.94624	-0.00129	1.56201

C	-1.98605	2.59224	0.46999
S	-1.13886	3.61455	-0.64654
C	-0.03331	2.34542	-1.05609
C	-0.34332	-2.25242	-1.36601
C	-1.34994	-3.15396	-1.19724
S	-2.46236	-2.67209	0.05122
C	1.80551	-0.14639	-0.08862
C	2.64163	0.97820	0.01948
C	3.99161	0.83227	0.32598
C	4.55253	-0.42657	0.52961
C	3.72597	-1.54413	0.43700
C	2.37269	-1.41306	0.13969
H	-2.84805	2.96328	1.00306
H	0.72405	2.51286	-1.80598
H	0.43642	-2.35264	-2.10982
H	-1.51675	-4.08048	-1.72615
H	2.24141	1.97258	-0.11894
H	4.60774	1.72253	0.40514
H	5.60661	-0.53351	0.76114
H	4.12997	-2.53708	0.60794
H	1.75553	-2.30078	0.10282

SCF Done: E(RB3LYP) = -1788.38342091 A.U. after 1 cycles

Zero-point correction= 0.190634 (Hartree/Particle)

Thermal correction to Energy= 0.205098

Thermal correction to Enthalpy= 0.206042

Thermal correction to Gibbs Free Energy= 0.147764

Sum of electronic and zero-point Energies= -1788.192787

Sum of electronic and thermal Energies= -1788.178323

Sum of electronic and thermal Enthalpies= -1788.177379

Sum of electronic and thermal Free Energies= -1788.235657

6.1.2.4 Computed xyz coordinates of radical cation 2b$^+$ of compound 2b (uB3LYP/6-311G* PCM CH_2Cl_2)

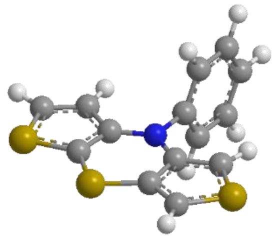

Figure 37. Optimized ground state geometry of radical cation of **2b** (uB3LYP/6-311G* PCM CH_2Cl_2).

C	1.52093	1.58371	-0.00005
C	0.12322	1.22004	0.00005
N	-0.32563	-0.09757	0.00008
C	0.53521	-1.16301	0.00002
C	1.93810	-1.03132	-0.00003
S	2.84505	0.43287	0.00022
C	1.71495	2.93745	-0.00015
S	0.21652	3.78043	-0.00013
C	-0.69408	2.32841	-0.00003
C	0.12807	-2.53546	0.00001
C	1.18443	-3.38513	-0.00001
S	2.72738	-2.57907	-0.00007
C	-1.75836	-0.34021	0.00006
C	-2.42731	-0.45110	1.21614
C	-3.80111	-0.68349	1.20924
C	-4.48563	-0.79971	-0.00003
C	-3.80098	-0.68383	-1.20925
C	-2.42717	-0.45145	-1.21605
H	2.64907	3.47781	-0.00024
H	-1.77126	2.35888	0.00002
H	-0.90237	-2.85823	0.00006
H	1.16371	-4.46476	-0.00004

H	-1.88178	-0.35859	2.14828
H	-4.33343	-0.77393	2.14916
H	-5.55452	-0.98156	-0.00005
H	-4.33317	-0.77453	-2.14923
H	-1.88155	-0.35919	-2.14816

SCF Done: E(UB3LYP) = -1788.20812116 A.U. after 1 cycles

Zero-point correction= 0.191468 (Hartree/Particle)

Thermal correction to Energy= 0.205931

Thermal correction to Enthalpy= 0.206875

Thermal correction to Gibbs Free Energy= 0.147770

Sum of electronic and zero-point Energies= -1788.016653

Sum of electronic and thermal Energies= -1788.002190

Sum of electronic and thermal Enthalpies= -1788.001246

Sum of electronic and thermal Free Energies= -1788.060351

6.1.2.5 Reoptimization of compound 2b (uB3LYP/6-311G*)

Intra conformation of compound **2b** in the gas phase (uB3LYP/6-311G*):

SCF Done: E(UB3LYP) = -1788.38068827 A.U. after 1 cycles
Zero-point correction= 0.190572 (Hartree/Particle)
Thermal correction to Energy= 0.205257
Thermal correction to Enthalpy= 0.206201
Thermal correction to Gibbs Free Energy= 0.146833
Sum of electronic and zero-point Energies= -1788.190116
Sum of electronic and thermal Energies= -1788.175431
Sum of electronic and thermal Enthalpies= -1788.174487
Sum of electronic and thermal Free Energies= -1788.233855

Intra conformation of compound **2b** in CH_2Cl_2 (uB3LYP/6-311G* SMD CH_2Cl_2):

SCF Done: E(UB3LYP) = -1788.40238287 A.U. after 13 cycles

Extra conformation of compound **2b** in the gas phase (uB3LYP/6-311G*):

SCF Done: E(UB3LYP) = -1788.37703184 A.U. after 1 cycles

Zero-point correction= 0.190735 (Hartree/Particle)

Thermal correction to Energy= 0.205188

Thermal correction to Enthalpy= 0.206132

Thermal correction to Gibbs Free Energy= 0.147890

Sum of electronic and zero-point Energies= -1788.186297

Sum of electronic and thermal Energies= -1788.171844

Sum of electronic and thermal Enthalpies= -1788.170900

Sum of electronic and thermal Free Energies= -1788.229142

Extra conformation of compound **2b** in CH_2Cl_2 (uB3LYP/6-311G* SMD CH_2Cl_2):

SCF Done: E(UB3LYP) = -1788.39877208 A.U. after 13 cycles

Radical cation of compound **2b** in the gas phase (uB3LYP/6-311G*):

SCF Done: E(UB3LYP) = -1788.15163876 A.U. after 1 cycles

Zero-point correction= 0.191341 (Hartree/Particle)

Thermal correction to Energy= 0.205854

Thermal correction to Enthalpy= 0.206798

Thermal correction to Gibbs Free Energy= 0.147335

Sum of electronic and zero-point Energies= -1787.960298

Sum of electronic and thermal Energies= -1787.945785

Sum of electronic and thermal Enthalpies= -1787.944840

Sum of electronic and thermal Free Energies= -1788.004303

Radical cation of compound **2b** in CH_2Cl_2 (uB3LYP/6-311G* SMD CH_2Cl_2):

SCF Done: E(UB3LYP) = -1788.22691472 A.U. after 19 cycles

6.1.3.1 Computed xyz coordinates of compound 2c (B3LYP/6-311G* PCM CH_2Cl_2)

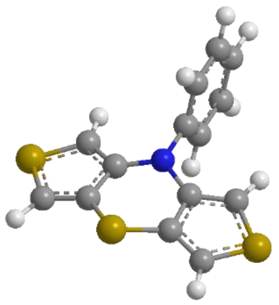

Figure 32. Optimized ground state geometry of **2c** (B3LYP/6-311G* PCM CH_2Cl_2).

C	-1.82222	-1.34089	0.11313
C	-0.39025	-1.22099	0.01252
N	0.28127	-0.00038	0.19775
C	-0.38820	1.22136	0.01257
C	-1.81993	1.34381	0.11334
S	-2.86642	0.00231	0.62245
C	-2.27108	-2.60988	-0.10261
S	-0.96484	-3.70772	-0.39782
C	0.20079	-2.42625	-0.26534
C	0.20496	2.42552	-0.26564
S	-0.95847	3.70909	-0.39798
C	-2.26656	2.61359	-0.10235
C	1.71989	-0.00148	0.15499
C	2.43664	-0.00175	1.35113
C	3.83039	-0.00259	1.32427
C	4.50600	-0.00325	0.10447
C	3.78716	-0.00292	-1.09080
C	2.39354	-0.00194	-1.06815
H	-3.29011	-2.96565	-0.08099
H	1.24910	-2.63928	-0.39809
H	1.25364	2.63655	-0.39865
H	-3.28495	2.97121	-0.08077
H	1.89818	-0.00129	2.29242
H	4.38683	-0.00281	2.25543
H	5.59070	-0.00398	0.08488
H	4.31014	-0.00335	-2.04124
H	1.82663	-0.00153	-1.99277

SCF Done: E(RB3LYP) = -1788.39151388 A.U. after 1 cycles

Zero-point correction= 0.190442 (Hartree/Particle)

Thermal correction to Energy= 0.205062

Thermal correction to Enthalpy= 0.206006

Thermal correction to Gibbs Free Energy= 0.147163

Sum of electronic and zero-point Energies= -1788.201071

Sum of electronic and thermal Energies= -1788.186452

Sum of electronic and thermal Enthalpies= -1788.185507

Sum of electronic and thermal Free Energies= -1788.244351

6.1.3.2 Computed excitations of compound 2c (B3LYP/6-311G* PCM CH_2Cl_2)

Excited State 1: Singlet-A 3.7819 eV 327.83 nm f=0.0125 <S**2>=0.000
74 -> 75 0.59834
74 -> 76 0.35385
This state for optimization and/or second-order correction.
Total Energy, E(TD-HF/TD-KS) = -1788.25253071
Copying the excited state density for this state as the 1-particle RhoCI density.

Excited State 2: Singlet-A 3.9191 eV 316.36 nm f=0.0537 <S**2>=0.000
74 -> 75 -0.34309
74 -> 76 0.59773
74 -> 78 -0.12096

Excited State 3: Singlet-A 4.1292 eV 300.26 nm f=0.0173 <S**2>=0.000
74 -> 77 0.69936

Excited State 4: Singlet-A 4.2243 eV 293.50 nm f=0.2299 <S**2>=0.000
74 -> 75 -0.10707
74 -> 78 0.67010
74 -> 80 -0.13592

Excited State 5: Singlet-A 4.5274 eV 273.85 nm f=0.0221 <S**2>=0.000
73 -> 75 0.21647
73 -> 80 0.21752
74 -> 78 0.13686
74 -> 80 0.61395

Excited State 6: Singlet-A 4.5688 eV 271.37 nm f=0.0003 <S**2>=0.000
74 -> 79 0.70038

Excited State   7:   Singlet-A   4.6114 eV 268.86 nm f=0.0163 <S**2>=0.000
      73 -> 75   0.60682
      73 -> 76   0.27473
      74 -> 80   -0.19628

Excited State   8:   Singlet-A   4.6973 eV 263.95 nm f=0.0194 <S**2>=0.000
      73 -> 75   -0.24911
      73 -> 76   0.64031

Excited State   9:   Singlet-A   4.7742 eV 259.69 nm f=0.0085 <S**2>=0.000
      73 -> 77   0.69637

Excited State 10:   Singlet-A   4.9228 eV 251.86 nm f=0.0238 <S**2>=0.000
      73 -> 78   0.67888
      73 -> 80   -0.10233

6.1.3.3 Computed xyz coordinates of extra conformation of compound 2c (B3LYP/6-311G* PCM CH_2Cl_2)

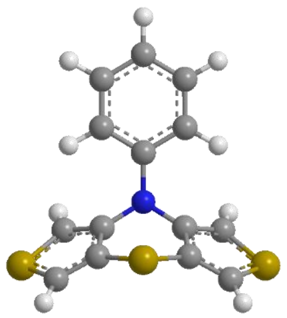

Figure 33. Optimized ground state geometry of extra conformation of **2c** (B3LYP/6-311G* PCM CH_2Cl_2).

C	1.51291	1.32019	0.54804
C	0.40986	1.18780	-0.36634
N	-0.37109	-0.00002	-0.40615
C	0.40992	-1.18778	-0.36634
C	1.51299	-1.32005	0.54804
S	1.92141	0.00008	1.67377
C	2.16200	2.51287	0.43712
S	1.42933	3.51337	-0.77721
C	0.27218	2.28612	-1.16832

C	0.27239	-2.28613	-1.16832
S	1.42967	-3.51325	-0.77719
C	2.16223	-2.51264	0.43714
C	-1.75806	-0.00005	-0.09643
C	-2.46522	-1.20396	0.06754
C	-3.82403	-1.19631	0.36941
C	-4.52204	-0.00019	0.51878
C	-3.82416	1.19596	0.36946
C	-2.46532	1.20377	0.06758
H	3.02126	2.86551	0.98689
H	-0.44983	2.45255	-1.95296
H	-0.44967	-2.45270	-1.95289
H	3.02156	-2.86517	0.98686
H	-1.95539	-2.15267	-0.02485
H	-4.33648	-2.14558	0.49105
H	-5.58191	-0.00027	0.74856
H	-4.33665	2.14521	0.49113
H	-1.95563	2.15255	-0.02482

SCF Done: E(RB3LYP) = -1788.38543013 A.U. after 1 cycles

Zero-point correction= 0.190631 (Hartree/Particle)

Thermal correction to Energy= 0.205051

Thermal correction to Enthalpy= 0.205996

Thermal correction to Gibbs Free Energy= 0.147678

Sum of electronic and zero-point Energies= -1788.194799

Sum of electronic and thermal Energies= -1788.180379

Sum of electronic and thermal Enthalpies= -1788.179435

Sum of electronic and thermal Free Energies= -1788.237752

6.1.3.4 Computed xyz coordinates of radical cation 2c⁺ of compound 2c (uB3LYP/6-311G* PCM CH_2Cl_2)

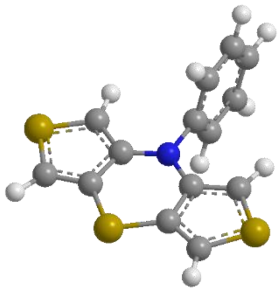

Figure 34. Optimized ground state geometry of radical cation of **2c** (uB3LYP/6-311G* PCM CH_2Cl_2).

C	-1.84393	-1.34236	0.00001
C	-0.40014	-1.21048	0.00001
N	0.26029	-0.00000	0.00001
C	-0.40013	1.21048	0.00001
C	-1.84393	1.34237	0.00001
S	-2.96813	0.00000	0.00030
C	-2.24184	-2.64913	-0.00013
S	-0.89155	-3.72214	-0.00015
C	0.23352	-2.44352	0.00000
C	0.23353	2.44352	-0.00000
S	-0.89154	3.72214	-0.00015
C	-2.24184	2.64914	-0.00013
C	1.71470	-0.00000	0.00002
C	2.39077	0.00001	1.21708
C	3.78399	0.00001	1.20954
C	4.47796	-0.00001	0.00006
C	3.78401	-0.00001	-1.20944
C	2.39081	-0.00001	-1.21704
H	-3.24754	-3.04022	-0.00024
H	1.29231	-2.64366	0.00007
H	1.29231	2.64366	0.00006
H	-3.24754	3.04023	-0.00024

H	1.83637	0.00001	2.14841
H	4.32398	0.00001	2.14935
H	5.56217	-0.00001	0.00007
H	4.32403	-0.00002	-2.14924
H	1.83645	-0.00002	-2.14839

SCF Done: E(UB3LYP) = -1788.20232791 A.U. after 1 cycles

Zero-point correction= 0.191003 (Hartree/Particle)

Thermal correction to Energy= 0.205500

Thermal correction to Enthalpy= 0.206444

Thermal correction to Gibbs Free Energy= 0.147283

Sum of electronic and zero-point Energies= -1788.011325

Sum of electronic and thermal Energies= -1787.996828

Sum of electronic and thermal Enthalpies= -1787.995884

Sum of electronic and thermal Free Energies= -1788.055045

6.1.3.5 Reoptimization of compound 2c (uB3LYP/6-311G*)

Intra conformation of compound **2c** in the gas phase (uB3LYP/6-311G*):

SCF Done: E(UB3LYP) = -1788.38535920 A.U. after 1 cycles
Zero-point correction= 0.190610 (Hartree/Particle)
Thermal correction to Energy= 0.205200
Thermal correction to Enthalpy= 0.206144
Thermal correction to Gibbs Free Energy= 0.147407
Sum of electronic and zero-point Energies= -1788.194750
Sum of electronic and thermal Energies= -1788.180159
Sum of electronic and thermal Enthalpies= 1788.179215
Sum of electronic and thermal Free Energies= -1788.237952

Intra conformation of compound **2c** in CH_2Cl_2 (uB3LYP/6-311G* SMD CH_2Cl_2):

SCF Done: E(UB3LYP) = -1788.40687300 A.U. after 13 cycles

Extra conformation of compound **2c** in the gas phase (uB3LYP/6-311G*):

SCF Done: E(UB3LYP) = -1788.37887704 A.U. after 1 cycles

Zero-point correction= 0.190740 (Hartree/Particle)

Thermal correction to Energy= 0.205148

Thermal correction to Enthalpy= 0.206092

Thermal correction to Gibbs Free Energy= 0.147855

Sum of electronic and zero-point Energies= -1788.188137

Sum of electronic and thermal Energies= -1788.173729

Sum of electronic and thermal Enthalpies= -1788.172785

Sum of electronic and thermal Free Energies= -1788.231022

Extra conformation of compound **2c** in CH_2Cl_2 (uB3LYP/6-311G* SMD CH_2Cl_2):

SCF Done: E(UB3LYP) = -1788.40070394 A.U. after 13 cycles

Radical cation of compound **2c** in the gas phase (uB3LYP/6-311G*):

SCF Done: E(UB3LYP) = -1788.14600330 A.U. after 1 cycles

Zero-point correction= 0.190951 (Hartree/Particle)

Thermal correction to Energy= 0.205458

Thermal correction to Enthalpy= 0.206402

Thermal correction to Gibbs Free Energy= 0.147069

Sum of electronic and zero-point Energies= -1787.955052

Sum of electronic and thermal Energies= -1787.940546

Sum of electronic and thermal Enthalpies= -1787.939602

Sum of electronic and thermal Free Energies= -1787.998934

Radical cation of compound **2c** in CH_2Cl_2 (uB3LYP/6-311G* SMD CH_2Cl_2):

SCF Done: E(UB3LYP) = -1788.22166994 A.U. after 18 cycles

6.1.4.1 Computed xyz coordinates of compound 2d (B3LYP/6-311G* PCM CH_2Cl_2)

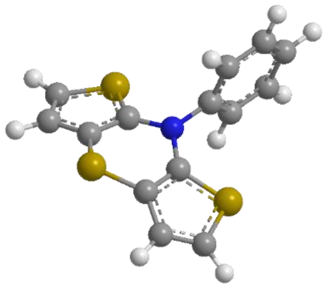

Figure 25. Optimized ground state geometry of **2d** (B3LYP/6-311G* PCM CH_2Cl_2).

C	-1.90116	-1.33370	0.22578
C	-0.55493	-1.19071	0.01390
N	0.16467	-0.00011	0.16614
C	-0.55436	1.19080	0.01364
C	-1.90052	1.33455	0.22545
S	-2.88058	0.00075	0.90939
S	0.14399	2.66274	-0.63408
C	-1.40452	3.46260	-0.56204
C	-2.38720	2.63274	-0.11475
C	-2.38853	-2.63172	-0.11407
C	-1.40631	-3.46222	-0.56116
S	0.14263	-2.66316	-0.63342
C	1.60230	-0.00034	0.21779
C	2.24356	0.00097	1.45743
C	3.63509	0.00082	1.51425
C	4.38337	-0.00067	0.33674
C	3.74056	-0.00197	-0.89994
C	2.34765	-0.00177	-0.96342
H	-1.47175	4.50594	-0.83097
H	-3.42071	2.93456	0.00114
H	-3.42218	-2.93299	0.00202
H	-1.47407	-4.50559	-0.82984
H	1.64801	0.00207	2.36347
H	4.13450	0.00185	2.47707
H	5.46714	-0.00081	0.38378
H	4.32060	-0.00311	-1.81637
H	1.83909	-0.00267	-1.92114

SCF Done: E(RB3LYP) = -1788.38186266   A.U. after   1 cycles

Zero-point correction= 0.189972 (Hartree/Particle)

Thermal correction to Energy= 0.204881

Thermal correction to Enthalpy= 0.205825

Thermal correction to Gibbs Free Energy= 0.145244

Sum of electronic and zero-point Energies= -1788.191890

Sum of electronic and thermal Energies= -1788.176982

Sum of electronic and thermal Enthalpies= -1788.176038

Sum of electronic and thermal Free Energies= -1788.236619

6.1.4.2 Computed excitations of compound 2d (B3LYP/6-311G* PCM CH_2Cl_2)

Excited State 1: Singlet-A 3.2372 eV 383.00 nm f=0.0609 <S**2>=0.000
74 -> 75 0.69419
This state for optimization and/or second-order correction.
Total Energy, E(TD-HF/TD-KS) = -1788.26289842
Copying the excited state density for this state as the 1-particle RhoCI density.

Excited State 2: Singlet-A 3.4536 eV 359.00 nm f=0.0023 <S**2>=0.000
74 -> 76 0.69883

Excited State 3: Singlet-A 3.6051 eV 343.91 nm f=0.0000 <S**2>=0.000
74 -> 77 0.70054

Excited State 4: Singlet-A 3.9338 eV 315.18 nm f=0.0290 <S**2>=0.000
74 -> 78 0.67032
74 -> 80 0.19148

Excited State 5: Singlet-A 4.1466 eV 299.00 nm f=0.0178 <S**2>=0.000
74 -> 79 0.69351

Excited State 6: Singlet-A 4.3283 eV 286.45 nm f=0.0109 <S**2>=0.000
74 -> 78 -0.18241
74 -> 80 0.67023

Excited State 7: Singlet-A 4.9021 eV 252.92 nm f=0.1317 <S**2>=0.000
73 -> 75 0.66069
74 -> 81 -0.16604
74 -> 84 0.10517

Excited State 8: Singlet-A 5.0217 eV 246.90 nm f=0.0383 <S**2>=0.000

72 -> 80 -0.11191
73 -> 75 0.12728
73 -> 76 0.17442
73 -> 77 0.11542
73 -> 79 -0.13802
74 -> 81 0.62464

Excited State 9: Singlet-A 5.0790 eV 244.11 nm f=0.0127 <S**2>=0.000
73 -> 76 0.67619
74 -> 81 -0.16458

Excited State 10: Singlet-A 5.1928 eV 238.76 nm f=0.0270 <S**2>=0.000
73 -> 75 -0.11010
73 -> 77 0.68145
74 -> 81 -0.11468

6.1.4.3 Computed xyz coordinates of S_1 of compound 2d (B3LYP/6-311G* PCM CH_2Cl_2)

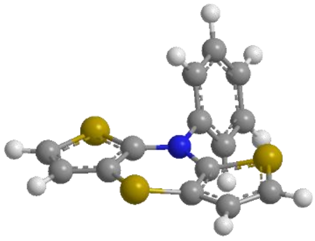

Figure 26. Optimized S_1 geometry of **2d** (B3LYP/6-311G* PCM CH_2Cl_2).

C	1.95631	1.32829	-0.15993
C	0.53983	1.19471	-0.16805
N	-0.13176	-0.00293	-0.04547
C	0.54906	-1.19066	0.01480
C	1.96504	-1.32735	0.02752
S	3.03600	0.00844	0.26029
S	-0.23889	-2.76455	-0.18906
C	1.34121	-3.55336	-0.21828
C	2.37859	-2.67920	-0.09049
C	2.36027	2.68884	-0.08852
C	1.32210	3.56285	0.03042

S	-0.26163	2.78058	0.09801
C	-1.57412	-0.00606	0.00331
C	-2.22181	-0.42494	1.16557
C	-3.61410	-0.43007	1.20277
C	-4.34724	-0.01969	0.08994
C	-3.68866	0.39745	-1.06682
C	-2.29725	0.40468	-1.11668
H	1.38207	-4.62493	-0.34312
H	3.41763	-2.98564	-0.08923
H	3.39717	3.00360	-0.10638
H	1.36611	4.64048	0.08709
H	-1.63977	-0.73092	2.02698
H	-4.12397	-0.74973	2.10479
H	-5.43124	-0.02527	0.12363
H	-4.25697	0.71217	-1.93501
H	-1.77174	0.71631	-2.01168

Excited State 1: Singlet-?Sym 2.1926 eV 565.47 nm f=0.0597 <S**2>=0.000

74 -> 75 0.70408

This state for optimization and/or second-order correction.

Total Energy, E(TD-HF/TD-KS) = -1788.28415837

SCF Done: E(RB3LYP) = -1788.36473410 A.U. after 7 cycles

Zero-point correction= 0.187239 (Hartree/Particle)

Thermal correction to Energy= 0.202844

Thermal correction to Enthalpy= 0.203788

Thermal correction to Gibbs Free Energy= 0.142779

Sum of electronic and zero-point Energies= -1788.096920

Sum of electronic and thermal Energies= -1788.081315

Sum of electronic and thermal Enthalpies= -1788.080371

Sum of electronic and thermal Free Energies= -1788.141380

6.1.4.4 Computed excitations of S_1 (emission of S_1) of compound 2d (B3LYP/6-311G* PCM CH_2Cl_2)

Excited State 1: Singlet-A 2.2118 eV 560.55 nm f=0.0436 <S**2>=0.000

74 -> 75 0.70329

This state for optimization and/or second-order correction.

Total Energy, E(TD-HF/TD-KS) = -1788.28345089

Copying the excited state density for this state as the 1-particle RhoCI density.

6.1.4.5 Computed xyz coordinates of extra conformation of compound 2d (B3LYP/6-311G* PCM CH_2Cl_2)

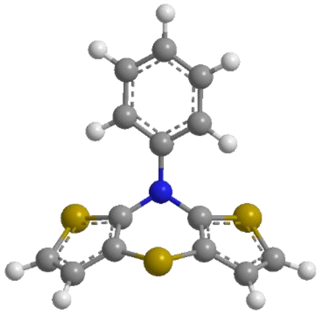

Figure 27. Optimized ground state geometry of extra conformation of **2d** (B3LYP/6-311G* PCM CH_2Cl_2).

C	1.47465	1.42957	0.55167
C	0.44201	1.16428	-0.30572
N	-0.26676	-0.05381	-0.36010
C	0.57757	-1.17569	-0.19247
C	1.63149	-1.21857	0.68232
S	2.01832	0.18789	1.71420
S	0.58485	-2.59169	-1.23302
C	1.95088	-3.24913	-0.38605
C	2.41221	-2.40910	0.58265
C	2.08392	2.70316	0.33491
C	1.50556	3.38013	-0.69663
S	0.23693	2.46965	-1.46162
C	-1.65173	-0.09464	0.00072
C	-2.27332	1.03097	0.55897

C	-3.62741	1.00087	0.88200
C	-4.38698	-0.14688	0.66623
C	-3.76822	-1.26963	0.12033
C	-2.41742	-1.24814	-0.21621
H	2.31959	-4.22864	-0.65279
H	3.26966	-2.62311	1.20761
H	2.90686	3.08894	0.92263
H	1.74087	4.36950	-1.06044
H	-1.69748	1.92572	0.75742
H	-4.08421	1.88375	1.31753
H	-5.44111	-0.16692	0.92043
H	-4.33911	-2.17560	-0.05575
H	-1.96868	-2.13386	-0.64531

SCF Done: E(RB3LYP) = -1788.38140110   A.U. after   1 cycles

Zero-point correction= 0.190430 (Hartree/Particle)

Thermal correction to Energy= 0.204998

Thermal correction to Enthalpy= 0.205943

Thermal correction to Gibbs Free Energy= 0.147288

Sum of electronic and zero-point Energies= -1788.190971

Sum of electronic and thermal Energies= -1788.176403

Sum of electronic and thermal Enthalpies= -1788.175458

Sum of electronic and thermal Free Energies= -1788.234114

6.1.4.6 Computed excitations of extra conformation of compound 2d (B3LYP/6-311G* PCM CH_2Cl_2)

Excited State   1:   Singlet-A   3.8539 eV 321.71 nm f=0.0327 <S**2>=0.000

74 -> 75   0.37034

74 -> 76   0.59063

This state for optimization and/or second-order correction.

Total Energy, E(TD-HF/TD-KS) = -1788.23977339

Copying the excited state density for this state as the 1-particle RhoCI density.

Excited State 2: Singlet-A 3.8866 eV 319.01 nm f=0.0691 <S**2>=0.000

74 -> 75 0.58877

74 -> 76 -0.36995

Excited State 3: Singlet-A 4.4049 eV 281.47 nm f=0.0089 <S**2>=0.000

73 -> 76 0.10036

74 -> 77 0.65314

74 -> 78 0.10490

74 -> 79 -0.16946

Excited State 4: Singlet-A 4.6266 eV 267.98 nm f=0.0835 <S**2>=0.000

73 -> 76 0.11720

74 -> 77 -0.20487

74 -> 78 0.50221

74 -> 79 -0.37785

74 -> 80 0.14738

Excited State 5: Singlet-A 4.6396 eV 267.23 nm f=0.0389 <S**2>=0.000

73 -> 75 -0.31344

73 -> 76 -0.12247

74 -> 78 0.35554

74 -> 79 0.46292

74 -> 80 0.15870

Excited State 6: Singlet-A 4.7587 eV 260.54 nm f=0.0712 <S**2>=0.000

72 -> 76 -0.11597

73 -> 75 0.59178

74 -> 78 0.24413

74 -> 79 0.20730

Excited State 7: Singlet-A 4.8419 eV 256.06 nm f=0.0630 <S**2>=0.000

73 -> 76 0.65579

74 -> 79 0.19874

Excited State 8: Singlet-A 4.9861 eV 248.66 nm f=0.0576 <S**2>=0.000

73 -> 75 0.13628

74 -> 78 -0.17689

74 -> 80 0.63864

Excited State 9: Singlet-A 5.0616 eV 244.95 nm f=0.0722 <S**2>=0.000

72 -> 75 0.68116

Excited State 10: Singlet-A 5.2380 eV 236.70 nm f=0.0380 <S**2>=0.000

71 -> 75 -0.18872

71 -> 78 -0.11184

72 -> 76 -0.22884

73 -> 77 0.57445

6.1.4.7 Computed xyz coordinates of radical cation 2d$^+$ of compound 2d (uB3LYP/6-311G* PCM CH_2Cl_2)

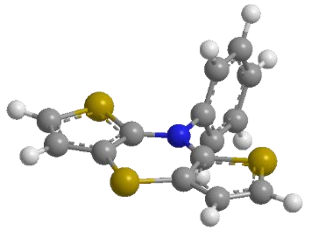

Figure 28. Optimized ground state geometry of radical cation of **2d** (uB3LYP/6-311G* PCM CH_2Cl_2).

C	-0.56144	1.18921	0.00009
C	-1.94859	1.33421	0.00008
S	-3.07397	0.00000	0.00001
C	-1.94859	-1.33421	-0.00007
C	-0.56144	-1.18921	-0.00009
N	0.12513	-0.00000	-0.00000

C	-2.35174	-2.69817	-0.00017
C	-1.28820	-3.54822	-0.00022
S	0.24745	-2.73177	-0.00016
S	0.24745	2.73177	0.00015
C	-1.28820	3.54822	0.00022
C	-2.35174	2.69817	0.00017
C	1.57977	-0.00000	0.00000
C	2.25517	-0.00083	1.21867
C	3.64774	-0.00084	1.21029
C	4.34046	-0.00000	0.00001
C	3.64774	0.00084	-1.21028
C	2.25517	0.00083	-1.21866
H	-3.38317	-3.02397	-0.00025
H	-1.30150	-4.62782	-0.00046
H	-1.30150	4.62782	0.00045
H	-3.38317	3.02397	0.00026
H	1.70017	-0.00137	2.14955
H	4.18848	-0.00151	2.14951
H	5.42462	0.00000	0.00001
H	4.18848	0.00151	-2.14950
H	1.70018	0.00137	-2.14955

SCF Done: E(UB3LYP) = -1788.21155791 A.U. after 1 cycles

Zero-point correction= 0.191376 (Hartree/Particle)

Thermal correction to Energy= 0.205915

Thermal correction to Enthalpy= 0.206859

Thermal correction to Gibbs Free Energy= 0.147561

Sum of electronic and zero-point Energies= -1788.020182

Sum of electronic and thermal Energies= -1788.005643

Sum of electronic and thermal Enthalpies= -1788.004699

Sum of electronic and thermal Free Energies= -1788.063997

6.1.4.8 Reoptimization of compound 2d (uB3LYP/6-311G*)

Intra conformation of compound **2d** in the gas phase (uB3LYP/6-311G*):

SCF Done: E(UB3LYP) = -1788.37510152 A.U. after 1 cycles

Zero-point correction= 0.190055 (Hartree/Particle)

Thermal correction to Energy= 0.204961

Thermal correction to Enthalpy= 0.205905

Thermal correction to Gibbs Free Energy= 0.145136

Sum of electronic and zero-point Energies= -1788.185047

Sum of electronic and thermal Energies= -1788.170141

Sum of electronic and thermal Enthalpies= -1788.169197

Sum of electronic and thermal Free Energies= -1788.229966

Intra conformation of compound **2d** in CH_2Cl_2 (uB3LYP/6-311G* SMD CH_2Cl_2):

SCF Done: E(UB3LYP) = -1788.39792497 A.U. after 13 cycles

Extra conformation of compound **2d** in the gas phase (uB3LYP/6-311G*):

SCF Done: E(UB3LYP) = -1788.37524185 A.U. after 1 cycles

Zero-point correction= 0.190467 (Hartree/Particle)

Thermal correction to Energy= 0.205047

Thermal correction to Enthalpy= 0.205991

Thermal correction to Gibbs Free Energy= 0.147102

Sum of electronic and zero-point Energies= -1788.184775

Sum of electronic and thermal Energies= -1788.170195

Sum of electronic and thermal Enthalpies= -1788.169251

Sum of electronic and thermal Free Energies= -1788.228139

Extra conformation of compound **2d** in CH_2Cl_2 (uB3LYP/6-311G* SMD CH_2Cl_2):

SCF Done: E(UB3LYP) = -1788.39697559 A.U. after 13 cycles

Radical cation of compound **2d** in the gas phase (uB3LYP/6-311G*):

SCF Done: E(UB3LYP) = -1788.15575888 A.U. after 1 cycles

Zero-point correction= 0.191255 (Hartree/Particle)

Thermal correction to Energy= 0.205812

Thermal correction to Enthalpy= 0.206756

Thermal correction to Gibbs Free Energy= 0.147528
Sum of electronic and zero-point Energies= -1787.964504
Sum of electronic and thermal Energies= -1787.949947
Sum of electronic and thermal Enthalpies= -1787.949003
Sum of electronic and thermal Free Energies= -1788.008231

Radical cation of compound **2d** in CH_2Cl_2 (uB3LYP/6-311G* SMD CH_2Cl_2):

SCF Done: E(UB3LYP) = -1788.23106757 A.U. after 17 cycles

6.1.5.1 Computed xyz coordinates of compound 1 (B3LYP/6-311G* PCM CH_2Cl_2)

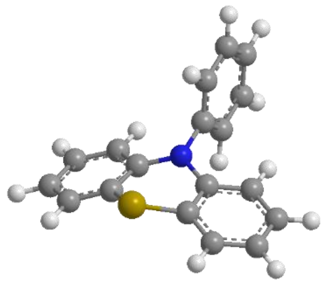

Figure 38. Optimized ground state geometry of **1** (B3LYP/6-311G* PCM CH_2Cl_2).

C	-1.79337	-1.35382	0.18137
C	-0.41726	-1.23540	-0.08761
N	0.25073	-0.00001	0.11397
C	-0.41724	1.23540	-0.08761
C	-1.79335	1.35385	0.18137
S	-2.66168	0.00002	0.95043
C	0.25898	2.37509	-0.54768
C	-0.41323	3.58068	-0.73737
C	-1.78115	3.67518	-0.50141
C	-2.46839	2.55050	-0.05032
C	-2.46844	-2.55045	-0.05032
C	-1.78122	-3.67516	-0.50140
C	-0.41330	-3.58067	-0.73737
C	0.25894	-2.37510	-0.54767
C	1.69187	-0.00002	0.15687

C	2.32447	-0.00001	1.39981
C	3.71672	-0.00002	1.47377
C	4.47893	-0.00003	0.30666
C	3.84675	-0.00003	-0.93668
C	2.45532	-0.00002	-1.01351
H	1.32035	2.32583	-0.74959
H	0.14361	4.44496	-1.08368
H	-2.30981	4.60836	-0.66093
H	-3.53391	2.60251	0.14779
H	-3.53396	-2.60244	0.14779
H	-2.30990	-4.60832	-0.66093
H	0.14353	-4.44498	-1.08367
H	1.32031	-2.32587	-0.74958
H	1.72059	-0.00001	2.30062
H	4.20388	-0.00002	2.44304
H	5.56224	-0.00004	0.36483
H	4.43571	-0.00004	-1.84770
H	1.95899	-0.00002	-1.97794

SCF Done: E(RB3LYP) = -1146.86633720 A.U. after 1 cycles

Zero-point correction= 0.257429 (Hartree/Particle)

Thermal correction to Energy= 0.272493

Thermal correction to Enthalpy= 0.273438

Thermal correction to Gibbs Free Energy= 0.214229

Sum of electronic and zero-point Energies= -1146.608908

Sum of electronic and thermal Energies= -1146.593844

Sum of electronic and thermal Enthalpies= -1146.592900

Sum of electronic and thermal Free Energies= -1146.652108

6.1.5.2 Computed excitations of compound 1 (B3LYP/6-311G* PCM CH_2Cl_2)

Excited State 1: Singlet-A 3.5764 eV 346.67 nm f=0.0020 <S**2>=0.000

72 -> 73 0.68941

72 -> 76 -0.11259

This state for optimization and/or second-order correction.

Total Energy, E(TD-HF/TD-KS) = -1146.73490644

Copying the excited state density for this state as the 1-particle RhoCI density.

Excited State 2: Singlet-A 3.9114 eV 316.98 nm f=0.0060 <S**2>=0.000

72 -> 74 0.66769

72 -> 76 -0.18158

Excited State 3: Singlet-A 4.0512 eV 306.04 nm f=0.0340 <S**2>=0.000

71 -> 73 -0.11785

72 -> 74 0.19956

72 -> 76 0.65578

Excited State 4: Singlet-A 4.0682 eV 304.76 nm f=0.0280 <S**2>=0.000

72 -> 75 0.68971

Excited State 5: Singlet-A 4.3064 eV 287.91 nm f=0.0647 <S**2>=0.000

71 -> 73 0.15342

71 -> 76 -0.11557

72 -> 77 0.66888

Excited State 6: Singlet-A 4.8647 eV 254.86 nm f=0.4817 <S**2>=0.000

71 -> 73 0.63595

72 -> 76 0.13088

72 -> 77 -0.16230

72 -> 79 0.14033

Excited State 7: Singlet-A 4.9571 eV 250.12 nm f=0.0939 <S**2>=0.000

70 -> 73 0.11606

71 -> 75 0.15176

72 -> 78 0.66047

Excited State 8: Singlet-A 4.9814 eV 248.89 nm f=0.0010 <S**2>=0.000

71 -> 73 -0.17084

71 -> 74 0.21204

71 -> 76 -0.17017

72 -> 79 0.61673

Excited State 9: Singlet-A 5.1136 eV 242.46 nm f=0.0439 <S**2>=0.000

71 -> 74 0.65182

72 -> 79 -0.23231

Excited State 10: Singlet-A 5.2240 eV 237.34 nm f=0.0450 <S**2>=0.000

71 -> 74 0.11747

71 -> 76 0.65402

72 -> 79 0.15669

6.1.5.3 Computed xyz coordinates of extra conformation of compound 1 (B3LYP/6-311G* PCM CH_2Cl_2)

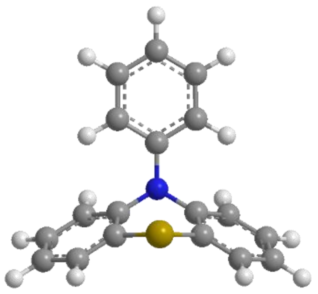

Figure 39. Optimized ground state geometry of extra conformation of **1** (B3LYP/6-311G* PCM CH_2Cl_2).

C	1.48026	-1.34557	-0.52325
C	0.45906	-1.20236	0.42582
N	-0.31350	-0.00006	0.44182
C	0.45880	1.20237	0.42589

C	1.48003	1.34581	-0.52311
S	1.78509	0.00021	-1.66228
C	0.25639	2.21509	1.36718
C	1.01353	3.38239	1.31675
C	2.00720	3.53162	0.34861
C	2.25654	2.50520	-0.55818
C	2.25693	-2.50483	-0.55844
C	2.00779	-3.53136	0.34830
C	1.01422	-3.38232	1.31655
C	0.25690	-2.21512	1.36709
C	-1.68693	-0.00019	0.09146
C	-2.38976	1.20415	-0.09677
C	-3.74004	1.19606	-0.43412
C	-4.43412	-0.00028	-0.60081
C	-3.74016	-1.19654	-0.43342
C	-2.38983	-1.20454	-0.09605
H	-0.50543	2.08483	2.12755
H	0.83640	4.16902	2.04202
H	2.60546	4.43563	0.31445
H	3.05415	2.59816	-1.28723
H	3.05451	-2.59767	-1.28754
H	2.60618	-4.43528	0.31402
H	0.83726	-4.16900	2.04181
H	-0.50485	-2.08499	2.12757
H	-1.88329	2.15401	0.00676
H	-4.24883	2.14510	-0.57243
H	-5.48690	-0.00029	-0.86125
H	-4.24897	-2.14565	-0.57113
H	-1.88350	-2.15441	0.00806

SCF Done: E(RB3LYP) = -1146.86333621   A.U. after   1 cycles

Zero-point correction= 0.257302 (Hartree/Particle)

Thermal correction to Energy= 0.272334

Thermal correction to Enthalpy= 0.273278

Thermal correction to Gibbs Free Energy= 0.214246

Sum of electronic and zero-point Energies= -1146.606034

Sum of electronic and thermal Energies= -1146.591002

Sum of electronic and thermal Enthalpies= -1146.590058

Sum of electronic and thermal Free Energies= -1146.649090

6.1.5.4 Computed xyz coordinates of radical cation 1+ of compound 1 (uB3LYP/6-311G* PCM CH2Cl2)

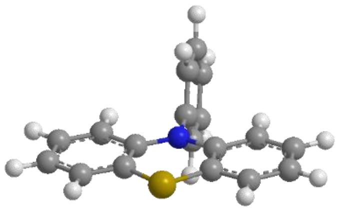

Figure 40. Optimized ground state geometry of **1** (uB3LYP/6-311G* PCM CH_2Cl_2).

C	-1.82928	-1.36070	-0.00007
C	-0.41488	-1.23827	-0.00003
N	0.22657	-0.00001	0.00001
C	-0.41482	1.23828	-0.00004
C	-1.82920	1.36079	-0.00001
S	-2.91181	0.00007	0.00005
C	0.34754	2.42904	-0.00012
C	-0.26671	3.66346	-0.00014
C	-1.66647	3.77127	-0.00008
C	-2.43624	2.62690	-0.00002
C	-2.43639	-2.62678	-0.00014
C	-1.66668	-3.77120	-0.00014
C	-0.26691	-3.66346	-0.00005
C	0.34741	-2.42908	-0.00000

C	1.68385	-0.00004	0.00006
C	2.36260	-0.00006	1.21529
C	3.75600	-0.00008	1.20906
C	4.45104	-0.00009	0.00017
C	3.75610	-0.00008	-1.20877
C	2.36270	-0.00005	-1.21511
H	1.42558	2.37367	-0.00018
H	0.34562	4.55725	-0.00020
H	-2.14207	4.74442	-0.00009
H	-3.51828	2.69356	0.00000
H	-3.51843	-2.69338	-0.00020
H	-2.14233	-4.74431	-0.00019
H	0.34536	-4.55728	-0.00003
H	1.42545	-2.37377	0.00006
H	1.80898	-0.00005	2.14730
H	4.29538	-0.00010	2.14931
H	5.53526	-0.00012	0.00022
H	4.29555	-0.00008	-2.14898
H	1.80915	-0.00003	-2.14716

SCF Done: E(UB3LYP) = -1146.68068878   A.U. after   1 cycles

Zero-point correction= 0.258680 (Hartree/Particle)

Thermal correction to Energy= 0.273666

Thermal correction to Enthalpy= 0.274610

Thermal correction to Gibbs Free Energy= 0.215260

Sum of electronic and zero-point Energies= -1146.422008

Sum of electronic and thermal Energies= -1146.407023

Sum of electronic and thermal Enthalpies= -1146.406079

Sum of electronic and thermal Free Energies= -1146.465429

6.1.5.5 Reoptimization of compound 1 (uB3LYP/6-311G*)

Intra conformation of compound **1** in the gas phase (uB3LYP/6-311G*):

SCF Done: E(UB3LYP) = -1146.85995451 A.U. after 1 cycles

Zero-point correction= 0.257455 (Hartree/Particle)

Thermal correction to Energy= 0.272510

Thermal correction to Enthalpy= 0.273454

Thermal correction to Gibbs Free Energy= 0.214283

Sum of electronic and zero-point Energies= -1146.602499

Sum of electronic and thermal Energies= -1146.587445

Sum of electronic and thermal Enthalpies= -1146.586500

Sum of electronic and thermal Free Energies= -1146.645671

Intra conformation of compound **1** in CH_2Cl_2 (uB3LYP/6-311G* SMD CH_2Cl_2):

SCF Done: E(UB3LYP) = -1146.88550803 A.U. after 13 cycles

Extra conformation of compound **1** in the gas phase (uB3LYP/6-311G*):

SCF Done: E(UB3LYP) = -1146.85695873 A.U. after 1 cycles

Zero-point correction= 0.257345 (Hartree/Particle)

Thermal correction to Energy= 0.272359

Thermal correction to Enthalpy= 0.273303

Thermal correction to Gibbs Free Energy= 0.214383

Sum of electronic and zero-point Energies= -1146.599613

Sum of electronic and thermal Energies= -1146.584599

Sum of electronic and thermal Enthalpies= -1146.583655

Sum of electronic and thermal Free Energies= -1146.642576

Extra conformation of compound **1** in CH_2Cl_2 (uB3LYP/6-311G* SMD CH_2Cl_2):

SCF Done: E(UB3LYP) = -1146.88245909 A.U. after 13 cycles

Radical cation of compound **1** in the gas phase (uB3LYP/6-311G*):

SCF Done: E(UB3LYP) = -1146.62706133 A.U. after 1 cycles

Zero-point correction= 0.258526 (Hartree/Particle)

Thermal correction to Energy= 0.273567

Thermal correction to Enthalpy= 0.274511

Thermal correction to Gibbs Free Energy= 0.214761

Sum of electronic and zero-point Energies= -1146.368535

Sum of electronic and thermal Energies= -1146.353495

Sum of electronic and thermal Enthalpies= -1146.352550

Sum of electronic and thermal Free Energies= -1146.412300

Radical cation of compound **1** in CH_2Cl_2 (uB3LYP/6-311G* SMD CH_2Cl_2):

SCF Done: E(UB3LYP) = -1146.70557160 A.U. after 17 cycles

6.1.6.1 Computed xyz coordinates of compound 2e (B3LYP/6-311G* PCM CH_2Cl_2)

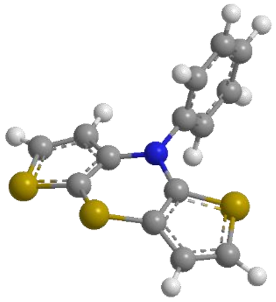

Figure 43. Optimized ground state geometry of **2e** (B3LYP/6-311G* PCM CH_2Cl_2).

C	2.05050	-0.78842	0.19107
C	0.72091	-1.06143	-0.01696
N	-0.28267	-0.08549	0.19445
C	0.09047	1.25138	0.04332
C	1.35509	1.75472	0.21189
S	2.67087	0.73248	0.86940
S	-0.99607	2.48907	-0.56097
C	0.28416	3.67512	-0.53170
C	1.46765	3.13604	-0.12875
S	3.05933	-2.13879	-0.24280
C	1.66525	-3.08951	-0.64961
C	0.50386	-2.39153	-0.49920
C	-1.67398	-0.44953	0.19150
C	-2.35004	-0.66319	-1.01242
C	-3.70071	-1.00778	-0.99604
C	-4.37462	-1.13697	0.21771
C	-3.69778	-0.92226	1.41809

C	-2.34723	-0.57968	1.40684
H	0.06026	4.69931	-0.78912
H	2.38710	3.70160	-0.04226
H	1.79252	-4.10927	-0.98068
H	-0.47196	-2.80530	-0.71373
H	-1.81892	-0.56023	-1.95241
H	-4.22466	-1.17436	-1.93110
H	-5.42577	-1.40494	0.22806
H	-4.21979	-1.02293	2.36358
H	-1.80733	-0.41135	2.33200

SCF Done: E(RB3LYP) = -1788.38209752 A.U. after 1 cycles

Zero-point correction= 0.190206 (Hartree/Particle)

Thermal correction to Energy= 0.205024

Thermal correction to Enthalpy= 0.205968

Thermal correction to Gibbs Free Energy= 0.146165

Sum of electronic and zero-point Energies= -1788.191892

Sum of electronic and thermal Energies= -1788.177073

Sum of electronic and thermal Enthalpies= -1788.176129

Sum of electronic and thermal Free Energies= -1788.235933

6.1.6.2 Computed xyz coordinates of radical cation 2e^{+} of compound 2e (uB3LYP/6-311G* PCM CH_2Cl_2)

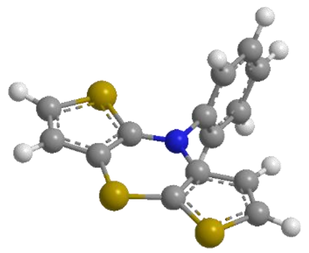

Figure 44. Optimized ground state geometry of radical cation of **2e** (uB3LYP/6-311G* PCM CH_2Cl_2).

C	-2.10316	-0.72281	0.00003
C	-0.74228	-1.03592	-0.00003

N	0.25592	-0.07293	-0.00005
C	-0.05695	1.25968	-0.00001
C	-1.34138	1.80702	0.00003
S	-2.80694	0.86334	0.00002
S	1.16307	2.50453	-0.00004
C	-0.06967	3.73317	-0.00000
C	-1.33389	3.22903	0.00003
S	-3.08622	-2.15211	0.00002
C	-1.66774	-3.15335	-0.00006
C	-0.50857	-2.44491	-0.00007
C	1.65160	-0.48042	-0.00001
C	2.30023	-0.67478	1.21733
C	3.63625	-1.06879	1.20975
C	4.30145	-1.26530	0.00005
C	3.63633	-1.06875	-1.20967
C	2.30030	-0.67474	-1.21731
H	0.23172	4.76994	0.00003
H	-2.22825	3.83762	0.00008
H	-1.78788	-4.22635	-0.00007
H	0.47281	-2.89568	-0.00009
H	1.76850	-0.52113	2.14920
H	4.15402	-1.22325	2.14937
H	5.34103	-1.57316	0.00009
H	4.15414	-1.22317	-2.14928
H	1.76863	-0.52106	-2.14921

SCF Done: E(UB3LYP) = -1788.21126114   A.U. after   1 cycles

Zero-point correction= 0.191446 (Hartree/Particle)

Thermal correction to Energy= 0.205963

Thermal correction to Enthalpy= 0.206908

Thermal correction to Gibbs Free Energy= 0.147814

Sum of electronic and zero-point Energies= -1788.019815

Sum of electronic and thermal Energies= -1788.005298

Sum of electronic and thermal Enthalpies= -1788.004353

Sum of electronic and thermal Free Energies= -1788.063447

6.1.6.3 Reoptimization of compound 2e (uB3LYP/6-311G*)

Intra conformation of compound **2e** in the gas phase (uB3LYP/6-311G*):

SCF Done: E(UB3LYP) = -1788.37547801 A.U. after 1 cycles
Zero-point correction= 0.190261 (Hartree/Particle)
Thermal correction to Energy= 0.205075
Thermal correction to Enthalpy= 0.206019
Thermal correction to Gibbs Free Energy= 0.146055
Sum of electronic and zero-point Energies= -1788.185217
Sum of electronic and thermal Energies= -1788.170403
Sum of electronic and thermal Enthalpies= -1788.169459
Sum of electronic and thermal Free Energies= -1788.229423

Intra conformation of compound **2e** in CH_2Cl_2 (uB3LYP/6-311G* SMD CH_2Cl_2):

SCF Done: E(UB3LYP) = -1788.39792198 A.U. after 14 cycles

Radical cation of compound **2e** in the gas phase (uB3LYP/6-311G*):

SCF Done: E(UB3LYP) = -1788.15514272 A.U. after 1 cycles
Zero-point correction= 0.191406 (Hartree/Particle)
Thermal correction to Energy= 0.205941
Thermal correction to Enthalpy= 0.206885
Thermal correction to Gibbs Free Energy= 0.147613
Sum of electronic and zero-point Energies= -1787.963737
Sum of electronic and thermal Energies= -1787.949202
Sum of electronic and thermal Enthalpies= -1787.948258
Sum of electronic and thermal Free Energies= -1788.007530

Radical cation of compound **2e** in CH_2Cl_2 (uB3LYP/6-311G* SMD CH_2Cl_2):
SCF Done: E(UB3LYP) = -1788.23037540 A.U. after 18 cycles

6.1.7.1 Computed xyz coordinates of compound 2f (B3LYP/6-311G* PCM CH_2Cl_2)

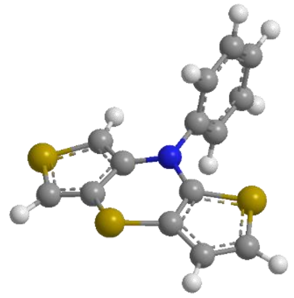

Figure 41. Optimized ground state geometry of **2f** (B3LYP/6-311G* PCM CH_2Cl_2).

C	2.18278	-0.70727	0.12766
C	0.77898	-1.01075	0.00606
N	-0.21644	-0.02885	0.17968
C	0.12728	1.31392	0.02375
C	1.38240	1.85257	0.13921
S	2.78493	0.88356	0.66015
C	2.97971	-1.79059	-0.08904
S	2.05024	-3.21966	-0.40732
C	0.56199	-2.33092	-0.28243
S	-1.03777	2.53675	-0.45022
C	0.20076	3.76668	-0.44254
C	1.42288	3.25356	-0.13470
C	-1.60198	-0.41676	0.16858
C	-2.25843	-0.65831	-1.04024
C	-3.60160	-1.03084	-1.03378
C	-4.28480	-1.16230	0.17468
C	-3.62533	-0.92194	1.37990
C	-2.28236	-0.55072	1.37909
H	4.05760	-1.83898	-0.05188
H	-0.37972	-2.83505	-0.42849
H	-0.07029	4.78991	-0.65346
H	2.32710	3.84740	-0.08178
H	-1.71836	-0.55403	-1.97485
H	-4.11277	-1.21725	-1.97214
H	-5.33022	-1.45201	0.17718
H	-4.15500	-1.02427	2.32090
H	-1.75545	-0.36212	2.30782

SCF Done: E(RB3LYP) = -1788.38728250 A.U. after 1 cycles

Zero-point correction= 0.190179 (Hartree/Particle)

Thermal correction to Energy= 0.204969

Thermal correction to Enthalpy= 0.205913

Thermal correction to Gibbs Free Energy= 0.146212

Sum of electronic and zero-point Energies= -1788.197103

Sum of electronic and thermal Energies= -1788.182313

Sum of electronic and thermal Enthalpies= -1788.181369

Sum of electronic and thermal Free Energies= -1788.241071

6.1.7.2 Computed xyz coordinates of radical cation 2f^{+} of compound 2f (uB3LYP/6-311G* PCM CH_2Cl_2)

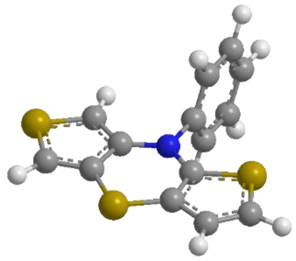

Figure 42. Optimized ground state geometry radical cation of **2f** (uB3LYP/6-311G* PCM CH_2Cl_2).

C	2.19913	-0.70269	0.00007
C	0.78646	-0.99895	-0.00004
N	-0.20065	-0.01099	-0.00002
C	0.11782	1.30771	-0.00009
C	1.41468	1.85344	0.00000
S	2.87345	0.91900	-0.00006
C	2.96781	-1.83332	0.00012
S	1.99509	-3.25366	0.00004
C	0.53758	-2.35190	-0.00004
S	-1.10298	2.55888	-0.00007
C	0.13907	3.78086	0.00004
C	1.40290	3.27364	0.00006
C	-1.59860	-0.41187	-0.00002

C	-2.24694	-0.60474	-1.21772
C	-3.58481	-0.99222	-1.20978
C	-4.25085	-1.18508	0.00007
C	-3.58482	-0.99196	1.20986
C	-2.24694	-0.60449	1.21770
H	4.04450	-1.90808	0.00014
H	-0.41656	-2.85299	-0.00017
H	-0.15673	4.81944	-0.00003
H	2.29685	3.88279	0.00013
H	-1.71428	-0.45382	-2.14943
H	-4.10346	-1.14409	-2.14931
H	-5.29204	-1.48741	0.00008
H	-4.10345	-1.14362	2.14945
H	-1.71426	-0.45337	2.14937

SCF Done: E(UB3LYP) = -1788.20952914   A.U. after   1 cycles

Zero-point correction= 0.191196 (Hartree/Particle)

Thermal correction to Energy= 0.205734

Thermal correction to Enthalpy= 0.206679

Thermal correction to Gibbs Free Energy= 0.147360

Sum of electronic and zero-point Energies= -1788.018333

Sum of electronic and thermal Energies= -1788.003795

Sum of electronic and thermal Enthalpies= -1788.002851

Sum of electronic and thermal Free Energies= -1788.062169

6.1.7.3 Reoptimization of compound 2f (uB3LYP/6-311G*)

Intra conformation of compound **2f** in the gas phase (uB3LYP/6-311G*):

SCF Done: E(UB3LYP) = -1788.38072441   A.U. after   1 cycles

Zero-point correction= 0.190287 (Hartree/Particle)

Thermal correction to Energy= 0.205059

Thermal correction to Enthalpy= 0.206003

Thermal correction to Gibbs Free Energy= 0.146321

Sum of electronic and zero-point Energies= -1788.190437
Sum of electronic and thermal Energies= -1788.175666
Sum of electronic and thermal Enthalpies= -1788.174722
Sum of electronic and thermal Free Energies= -1788.234403

Intra conformation of compound **2f** in CH_2Cl_2 (uB3LYP/6-311G* SMD CH_2Cl_2):

SCF Done: E(UB3LYP) = -1788.40300796 A.U. after 13 cycles

Radical cation of compound **2f** in the gas phase (uB3LYP/6-311G*):

SCF Done: E(UB3LYP) = -1788.15341717 A.U. after 1 cycles
Zero-point correction= 0.191177 (Hartree/Particle)
Thermal correction to Energy= 0.205698
Thermal correction to Enthalpy= 0.206643
Thermal correction to Gibbs Free Energy= 0.147418
Sum of electronic and zero-point Energies= -1787.962240
Sum of electronic and thermal Energies= -1787.947719
Sum of electronic and thermal Enthalpies= -1787.946775
Sum of electronic and thermal Free Energies= -1788.006000

Radical cation of compound **2f** in CH_2Cl_2 (uB3LYP/6-311G* SMD CH_2Cl_2):

SCF Done: E(UB3LYP) = -1788.22904361 A.U. after 19 cycles

6.2 DFT calculation of the redox potentials of compounds 1 and 2

The calculation of the redox potentials (Table 6) was carried out by adapting a literature procedure (equation 1) based on DFT methods.[12]

$$E_{redox} = \frac{\Delta G_{redox}(solv)}{-F} = \frac{\Delta G_{redox}(gas)+\Delta G_{solv}(ox)-\Delta G_{solv}(red)}{-F} \qquad \text{(equation 1)}$$

E_{redox}	redox potential vs. vacuum
ΔG_{redox}(solv/gas)	free enthalpy of oxidation in solution/gas phase
ΔG_{solv}(ox/red)	free enthalpy of solvation of the oxidized/reduced compound
F	Faraday constant (23.06 kcal $mol^{-1}\cdot V^{-1}$)

ΔG_{solv}(gas), ΔG_{solv}(solv) and ΔG_{redox}(gas) were calculated from the values of the free enthalpies obtained from the geometry optimizations given in chapter 6.1 (uB3LYP/6-311G*). The SMD solvation model[10] with dichloromethane as a solvent was applied to determine the solvation enthalpies, since all experimental determined oxidation potentials were measured in dichloromethane solutions.

Table 6. Calculated ΔG_{solv}, ΔG_{redox} and $E_{redox}^{0/+1}$ of intra conformations of compounds **1** and **2** (uB3LYP/6-311G* SMD CH_2Cl_2).

Compound (intra conf.)	ΔG_{redox}(gas) [kcal/mol]	ΔG_{solv}(red) [kcal/mol]	ΔG_{solv}(ox) [kcal/mol]	ΔG_{redox}(solv) [kcal/mol]	$E_{redox}^{0/+1}$ vs. vacuum [V]
**1**	146.3447	-16.0244	-49.2330	113.1361	4.9093
**2a**	139.5717	-13.8269	-46.9969	106.4017	4.6171
**2b**	143.9494	-13.6044	-47.2048	110.3491	4.7884
**2c**	149.8857	-13.4911	-47.4498	115.9270	5.0304
**2d**	139.0475	-14.3124	-47.2253	106.1346	4.6055
**2e**	139.1470	-14.0744	-47.1777	106.0438	4.6016
**2f**	143.2299	-13.9738	-47.4246	109.7792	4.7637

For a linear correlation of the measured first oxidation potential $E_{0/+1}^{exp}$ with the calculated first oxidation potential $E_{0/+1}^{cal}$ of the compounds **1** and **2** in their intra conformations in the gas-phase, see figure 45.

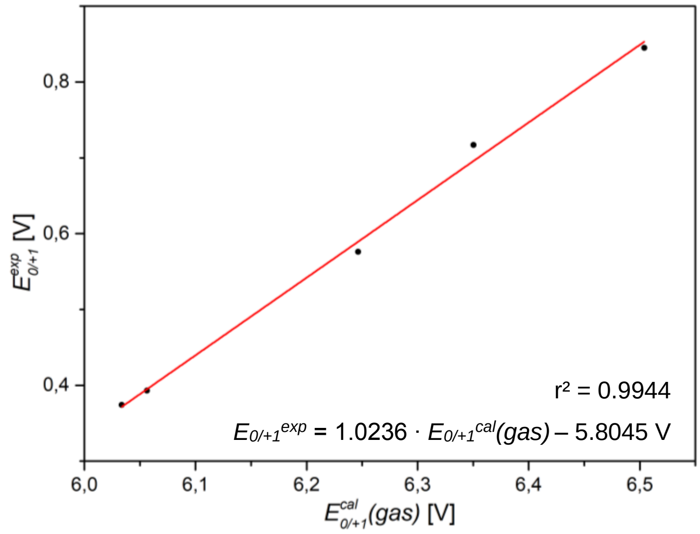

Figure 45. Linear correlation of the measured first oxidation potential $E_{0/+1}^{exp}$ (0.1 M $[Bu_4N][PF_6]$, v = 100 mV/s, Pt-working, Ag/AgCl-reference and Pt-counter electrode, $[Me_{10}Fc]/[Me_{10}Fc]^+$ as an internal standard) and the calculated first oxidation potential $E_{0/+1}^{cal}$ (vs. vacuum, uB3LYP/6-311G*) of the compounds **1** and **2** in their intra conformations in the gas-phase.

The redox potentials were also calculated for the extra conformers revealing a poorer correlation with the experimental results (Table 7, Figure 46), which indicates that the redox behavior of the dithienothiazines **2** is dominated by the intra conformations respectively.

Table 7. Calculated ΔG_{solv}, ΔG_{redox} and $E_{redox}^{0/+1}$ of extra conformations of compounds **1** and **2** (uB3LYP/6-311G* SMD CH_2Cl_2).

Compound (extra conf.)	ΔG_{redox}(gas) [kcal/mol]	ΔG_{solv}(red) [kcal/mol]	ΔG_{solv}(ox), [kcal/mol]	ΔG_{redox}(solv) [kcal/mol]	$E_{redox}^{0/+1}$ vs. vacuum [V]
**1**	144.4034	-15.9910	-49.2330	111.1614	4.8236
**2a**	138.6395	-13.4095	-46.9969	105.0521	4.5585
**2b**	140.9936	-13.6331	-47.2048	107.4220	4.6614
**2c**	145.5398	-13.6875	-47.4498	111.7775	4.8504
**2d**	137.9027	-13.6290	-47.2253	104.3064	4.5262

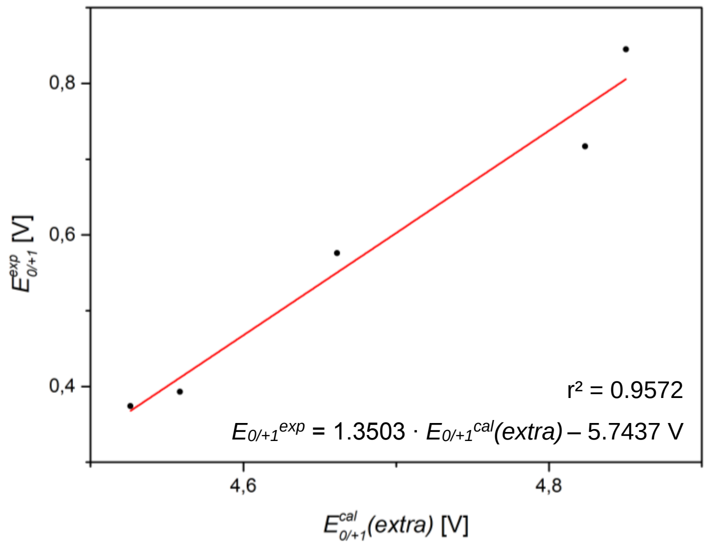

Figure 46. Linear correlation of the measured first oxidation potential $E_{0/+1}^{exp}$ (0.1 M $[Bu_4N][PF_6]$, v = 100 mV/s, Pt-working, Ag/AgCl-reference and Pt-counter electrode, $[Me_{10}Fc]/[Me_{10}Fc]^+$ as an internal standard) and the calculated first oxidation potential $E_{0/+1}^{cal}$ (vs. vacuum, uB3LYP/6-311G*, SMD CH_2Cl_2) of the compounds **1** and **2** in their <u>extra</u> conformations.

From the calculated differences of the free enthalpies of the *extra* and *intra* conformers $\Delta G_{extra \rightarrow intra}$ (see chapter 6.1) we calculated the equilibrium mole fraction of the *extra* and *intra* conformers assuming a Boltzmann distribution. Given the mole fractions, we calculated the effective redox potentials consisting of the oxidation potentials of both conformers further suggesting, that the *intra* conformers dominate the electrochemical properties at least in CV experiments (Figure 47).

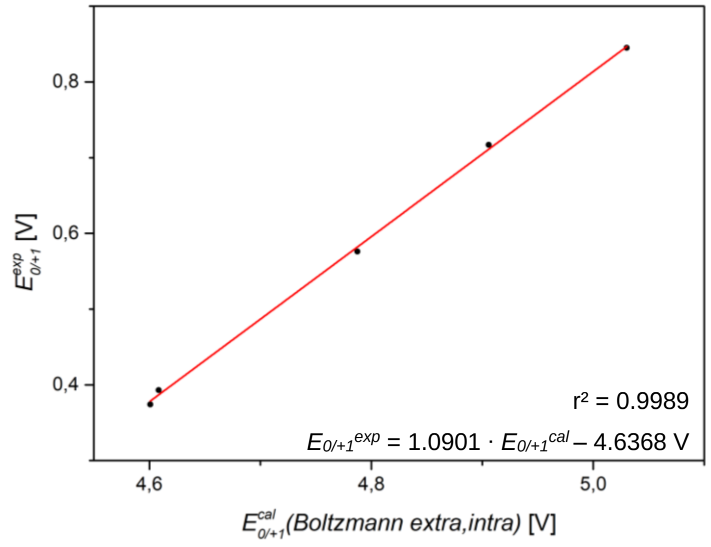

Figure 47. Linear correlation of the measured first oxidation potential $E_{0/+1}^{exp}$ (0.1 M $[Bu_4N][PF_6]$, v = 100 mV/s, Pt-working, Ag/AgCl-reference and Pt-counter electrode, $[Me_{10}Fc]/[Me_{10}Fc]^+$ as an internal standard) and the calculated first oxidation potential $E_{0/+1}^{cal}$ (vs. vacuum, uB3LYP/6-311G*, SMD CH_2Cl_2) of the compounds **1** and **2** in their <u>intra and extra</u> conformations in a Boltzmann distribution.

7 References

[1] C.-T. Li, F.-L. Wu, C.-J. Liang, K.-C. Ho, J. T. Lin, *Effective suppression of interfacial charge recombination by a 12-crown-4 substituent on a double-anchored organic sensitizer and rotating disk electrochemical evidence J. Mater. Chem. A* **2017**,*5,*7586–7594. DOI: 10.1039/c6ta11091f

[2] C. Dostert, C. Wansrath, W. Frank, T. J. J. Müller, *4H-Dithieno[2,3-b:30,20-e][1,4]thiazines – synthesis and electronic properties of a novel class of electron rich redox system Chem. Commun,* ***2012****, 48,* 7271–7273. DOI: 10.1039/c2cc32731g

[3] P. Zanello, in: *Ferrocenes* A. Togni (Ed.), T. Hayashi (Ed.), Wiley VHC, Weinheim **1995**, 317-430.

[4] Literature known compound; for the synthesis via single-step transformations, see: J. De Jong, *The Synthesis, Oxidation, and Electronic Spectra of Four Dithienothiophenes* J. Org. Chem. **1971**, *36*, 1645-1648. DOI: 10.1021/jo00811a016

[5] Gaussian 09, Revision A.02, M. J. Frisch, G. W. Trucks, H. B. Schlegel, G. E. Scuseria, M. A. Robb, J. R. Cheeseman, G. Scalmani, V. Barone, B. Mennucci, G. A. Petersson, H. Nakatsuji, M. Caricato, X. Li, H. P. Hratchian, A. F. Izmaylov, J. Bloino, G. Zheng, J. L. Sonnenberg, M. Hada, M. Ehara, K. Toyota, R. Fukuda, J. Hasegawa, M. Ishida, T. Nakajima, Y. Honda, O. Kitao, H. Nakai, T. Vreven, J. A. Montgomery, Jr., J. E. Peralta, F. Ogliaro, M. Bearpark, J. J. Heyd, E. Brothers, K. N. Kudin, V. N. Staroverov, R. Kobayashi, J. Normand, K. Raghavachari, A. Rendell, J. C. Burant, S. S. Iyengar, J. Tomasi, M. Cossi, N. Rega, J. M. Millam, M. Klene, J. E. Knox, J. B. Cross, V. Bakken, C. Adamo, J. Jaramillo, R. Gomperts, R. E. Stratmann, O. Yazyev, A. J. Austin, R. Cammi, C. Pomelli, J. W. Ochterski, R. L. Martin, K. Morokuma, V. G. Zakrzewski, G. A. Voth, P. Salvador, J. J. Dannenberg, S. Dapprich, A. D. Daniels, O. Farkas, J. B. Foresman, J. V. Ortiz, J. Cioslowski, and D. J. Fox, Gaussian, Inc., Wallingford CT, 2009.

[6] (a) A. D. Becke, *Density‐functional thermochemistry. III. The role of exact exchange*. *J. Chem. Phys.* **1993**, *98*, 5648-5652. DOI: 10.1063/1.464913; (b) A. D. Becke, *A new mixing of Hartree–Fock and local density‐functional theories*. *J. Chem. Phys.* **1993**, *98*, 1372-1377, DOI: 10.1063/1.464304.

[7] (a) R. Krishnan, J. S. Binkley, R. Seeger, J. A. Pople, *Self‐consistent molecular orbital methods. XX. A basis set for correlated wave functions*. *J. Chem. Phys.* **1980**, *72*, 650-654. DOI: 10.1063/1.438955; (b) A. D. McLean, G. S. Chandler, *Contracted Gaussian*

basis sets for molecular calculations. I. Second row atoms, Z=11–18. J. Chem. Phys. **1980**, *72*, 5639-5648. DOI: 10.1063/1.438980.

[8] (a) Bauernschmitt R, Ahlrichs R., *Treatment of electronic excitations within the adiabatic approximation of time dependent density functional theory Chem Phys Lett* **1996**, *256*, 454-464. DOI: 10.1016/0009-2614(96)00440-X; (b) M. E. Casida, C. Jamorski, K. C. Casida, D. R. Salahub, *Molecular excitation energies to high-lying bound states from time-dependent density-functional response theory: Characterization and correction of the time-dependent local density approximation ionization threshold J Chem Phys* **1998**, *108*, 4439-4449. DOI: 10.1063/1.475855; (c) R. E. Stratmann, G. E. Scuseria, M. J. Frisch, *An efficient implementation of time-dependent density-functional theory for the calculation of excitation energies of large molecules J Chem Phys* **1998**, *109*, 8218-8224. DOI: 10.1063/1.477483

[9] G. Scalmani, M. J. Frisch, *Continuous surface charge polarizable continuum models of solvation. I. General formalism J. Chem. Phys.* **2010**, *132*, 114110-114115. DOI 10.1063/1.3359469

[10] A. V. Marenich, C. J. Cramer, D. G. Truhlar, *Universal solvation model based on solute electron density and a continuum model of the solvent defined by the bulk dielectric constant and atomic surface tensions J. Phys. Chem. B* **2009**, *113*, 6378-6396. DOI: 10.1021/jp810292n

[11] T. Lu, F. Chen, *Multiwfn: a multifunctional wavefunction analyzer. J. Comput. Chem.* **2012**, *33*, 580-592. DOI: 10.1002/jcc.22885

[12] J. Li, C. L. Fischer, J. L. Chen, D. *Bashford, Calculation of Redox Potentials and pK_a Values of Hydrated Transition Metal Cations by a Combined Density Functional and Continuum Dielectric Theory Inorg. Chem.* **1996**, *35,* 4694-4702. DOI: 10.1021/ic951428f
